# Supplementary material for: ORF3c is expressed in SARS‐CoV‐2‐infected cells and inhibits innate sensing by targeting MAVS
Source: EMBO Rep. 2023 Oct 23;24(12):e57137. doi: 10.15252/embr.202357137 (PMC10702836; doi:10.15252/embr.202357137)
Supplement: Supplementary file 1 — Expanded View Figures PDF [file EMBR-24-e57137-s006.pdf]

Expanded View Figures

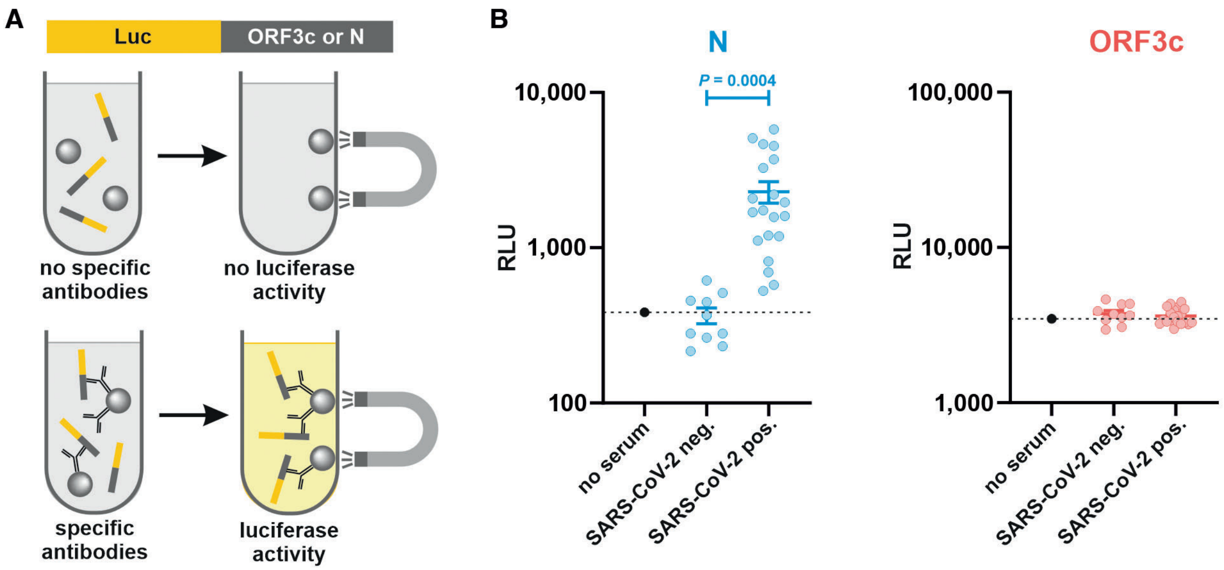

Figure EV1. Detection of SARS-CoV-2-specific antibodies via Luciferase Immunoprecipitation System (LIPS).

A Principle of the LIPS assay: HEK293T cells are transfected with expression plasmids for a viral protein of interest fused to Renilla luciferase. Subsequently, transfected cells are lysed and incubated with serum samples and magnetic beads. Antibodies against viral proteins of interest will cross-link the luciferase-containing proteins with beads and allow magnet-assisted pull-down of both beads and luciferase activity.

B LIPS-mediated quantification of antibodies against SARS-CoV-2 N (left panel, n = 10) and ORF3c (right panel, n = 20) in sera from SARS-CoV-2 naïve and convalescent sera (RLU, relative light units). Each dot represents one independent serum sample. Data are shown as mean ± s.e.m. Differences in antibody levels between SARS-CoV-2 naïve and convalescent sera were determined by unpaired, one-tailed student's t-test.

Source data are available online for this figure.

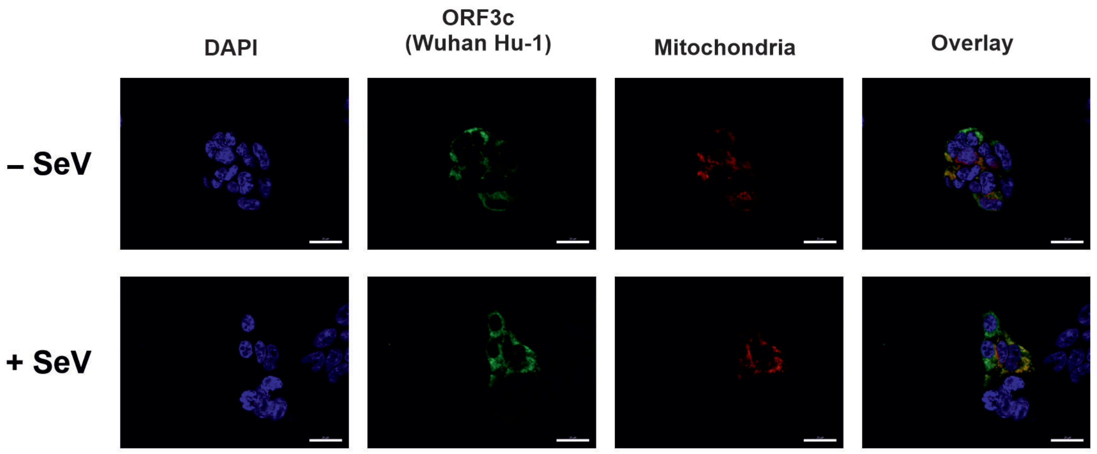

Figure EV2. ORF3c co-localizes with mitochondria.

Representative images of HEK293T cells transfected with the expression plasmid dsRedMito coding for a mitochondria marker, as well as expression plasmids for Wuhan Hu-1 ORF3c or an empty vector control. One day post-transfection, cells were infected with SeV for 6 h and subsequently stained for ORF3c (anti-HA, green) and nuclei (DAPI, blue) (scale bar = 20 μm).

Source data are available online for this figure.

© 2023 The Authors

Figure EV3. ORF3c affects IRF3 activation and translocation but not total protein levels.

A HEK293T cells were transfected with either Wuhan Hu-1 ORF3c or an empty vector control and stimulated with SeV 6 h p.t. Cells were harvested at the indicated time points, and (phosphorylated) IRF3 was analyzed by western blotting. Data are representative of three biological replicates (n = 3).

B Representative images of HEK293T cells transfected with expression plasmids for Wuhan Hu-1 ORF3c or an empty vector control. One day post-transfection, cells were treated with SeV for 6 h and subsequently stained for ORF3c (anti-HA, green), IRF3 (endogenous, red) and nuclei (DAPI, blue) (scale bar = 20 μm). Weighted colocalization coefficient of IRF3 with DAPI was determined as a marker for IRF3 translocation into the nucleus (n = 28–32 cells in each condition analyzed). Data are shown as individual cells with the median and quartiles indicated as dotted lines (right panels).

C HEK293T cells were transfected with increasing amounts of expression plasmids for Wuhan Hu-1 ORF3c and IRES eGFP. Transfection with expression plasmids for NSP1 from the rotaviruses NCDV (reduces IRF3 expression) or OSU (inactive against IRF3) (both co-expressing YFP) served as controls. One day post-transfection cells were fixed, permeabilized and stained against IRF3 (endogenous, AF555) before FACS analysis. Numbers in gates indicate MFI of IRF3-AF555 (left). Levels of IRF3 expression are shown as MFI of AF555 in GFP-positive over GFP-negative cells. Data are shown as bars (min to max) with median of three biological replicates (n = 3) and were analyzed by one-way ANOVA with Dunnett's multiple-comparison test.

Source data are available online for this figure.

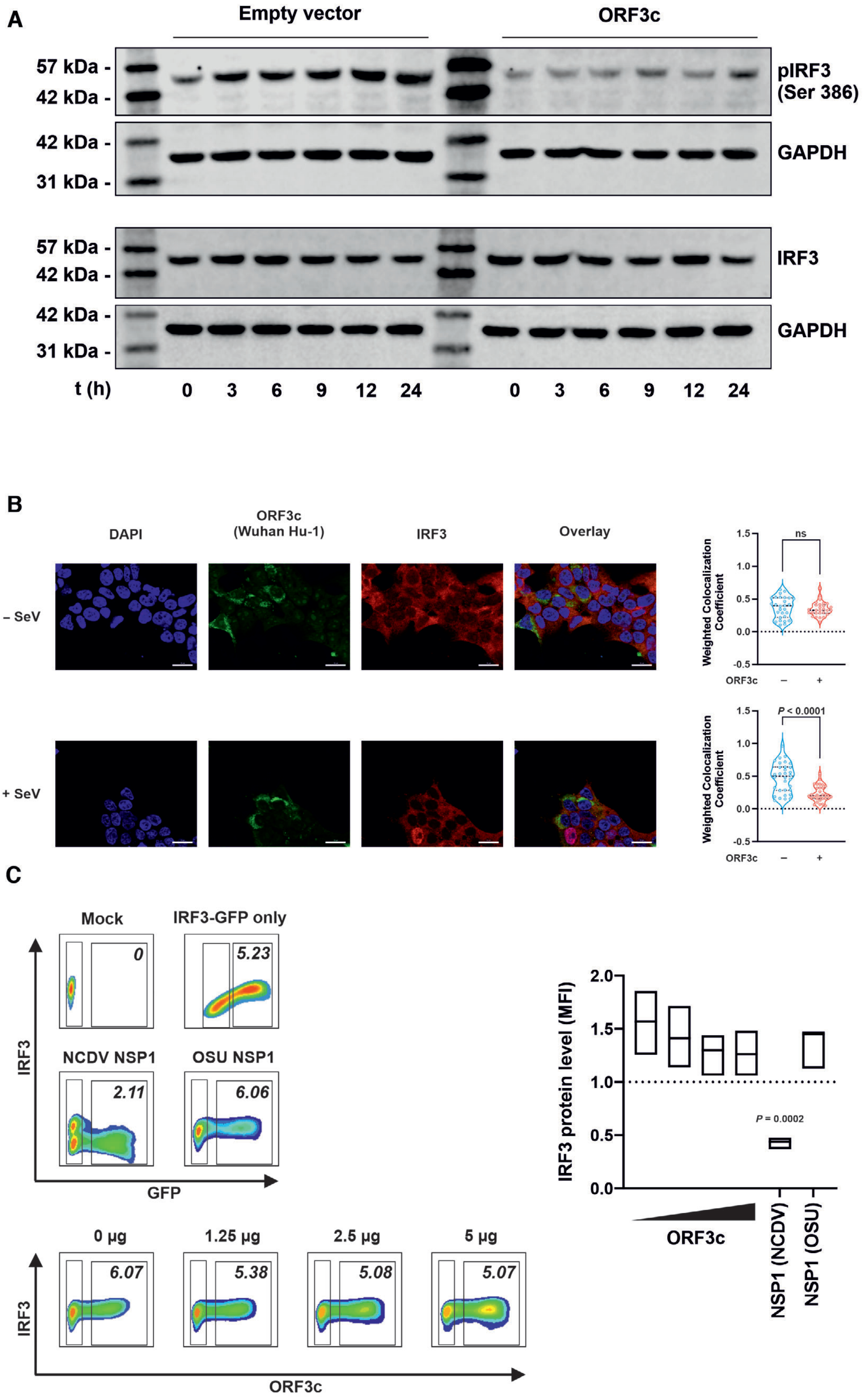

Figure EV3.

 © 2023 The Authors

Figure EV4. Targeting of MAVS by different ORF3c orthologs and mutants.

A HEK293T cells were co-transfected with expression plasmids for Flag-tagged MAVS and expression plasmids for the indicated HA-tagged SARS-CoV-2 ORF3c variants. One day post-transfection, cells were lysed. Cell lysates were analyzed by Western blotting, either directly ("input") or upon pull-down using a Flag-specific antibody ("IP"). Data are representative of two biological replicates ($n = 2$). The red arrow indicates a faint ORF3c band.

B HEK293T cells were co-transfected with expression plasmids for blue fluorescent protein (BFP) and expression plasmids for the indicated HA-tagged SARS-CoV-2 ORF3c variants. One day post-transfection, cells were permeabilized, stained for the HA-tag, and analyzed by flow cytometry. BFP-positive (i.e. transfected) cells were gated, and the percentage of HA-ORF3c-positive (i.e. PE-positive) cells was determined. Exemplary primary data of one out of three biological replicates are shown.

C HEK293T cells were transfected with plasmids expressing BFP, the indicated ORF3c orthologs, the C-terminal part of YFP fused to RIG-I and/or the N-terminal part of YFP fused to MAVS. Twenty-four hours later, cells were fixed, and YFP fluorescence was detected by flow cytometry as a reporter for MAVS-RIG-I interaction. Data are shown as mean and s.d. of three biological replicates ($n = 3$) and were analyzed by one-way ANOVA with Dunnett's multiple-comparison test.

Source data are available online for this figure.

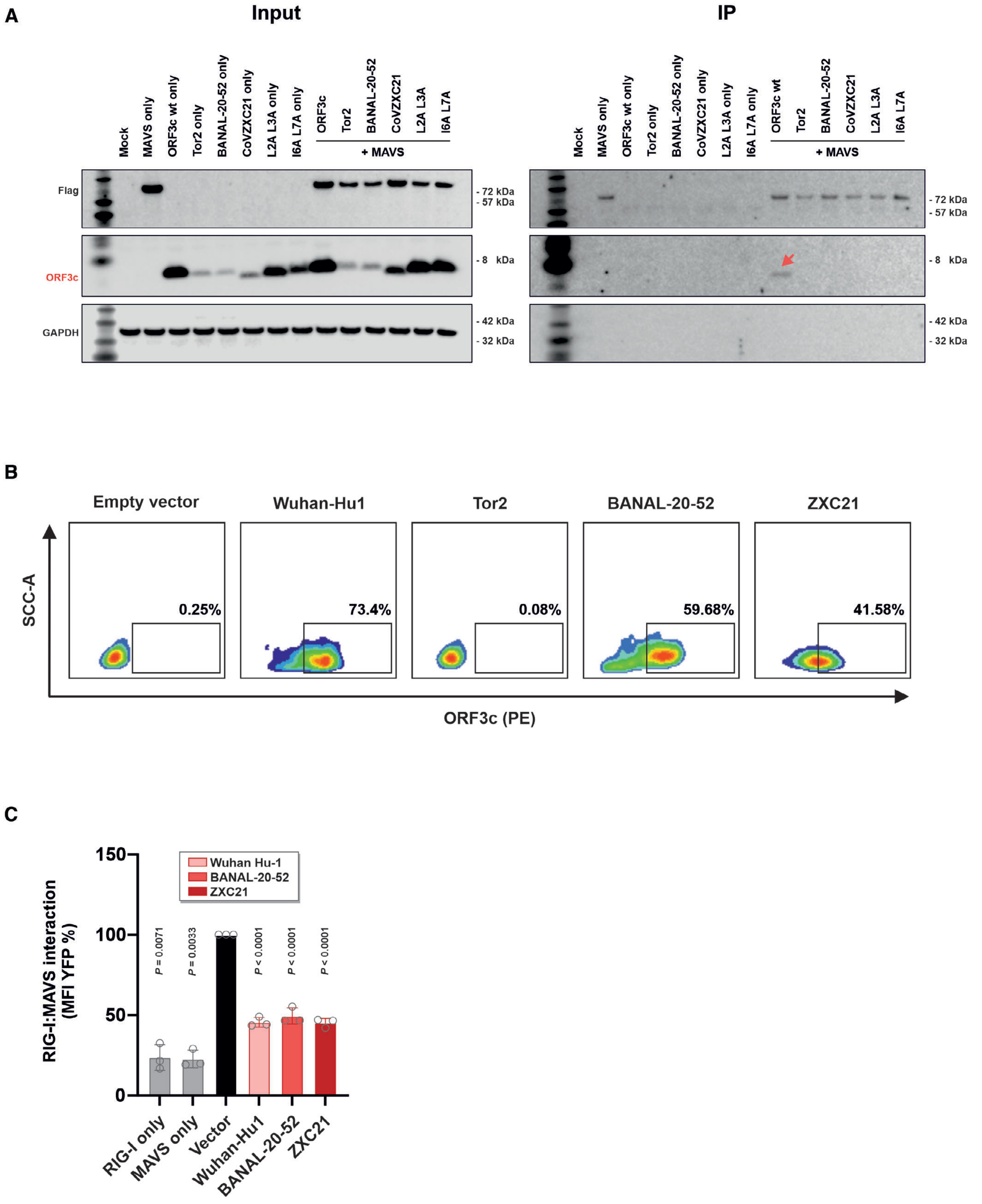

Figure EV4.

 © 2023 The Authors

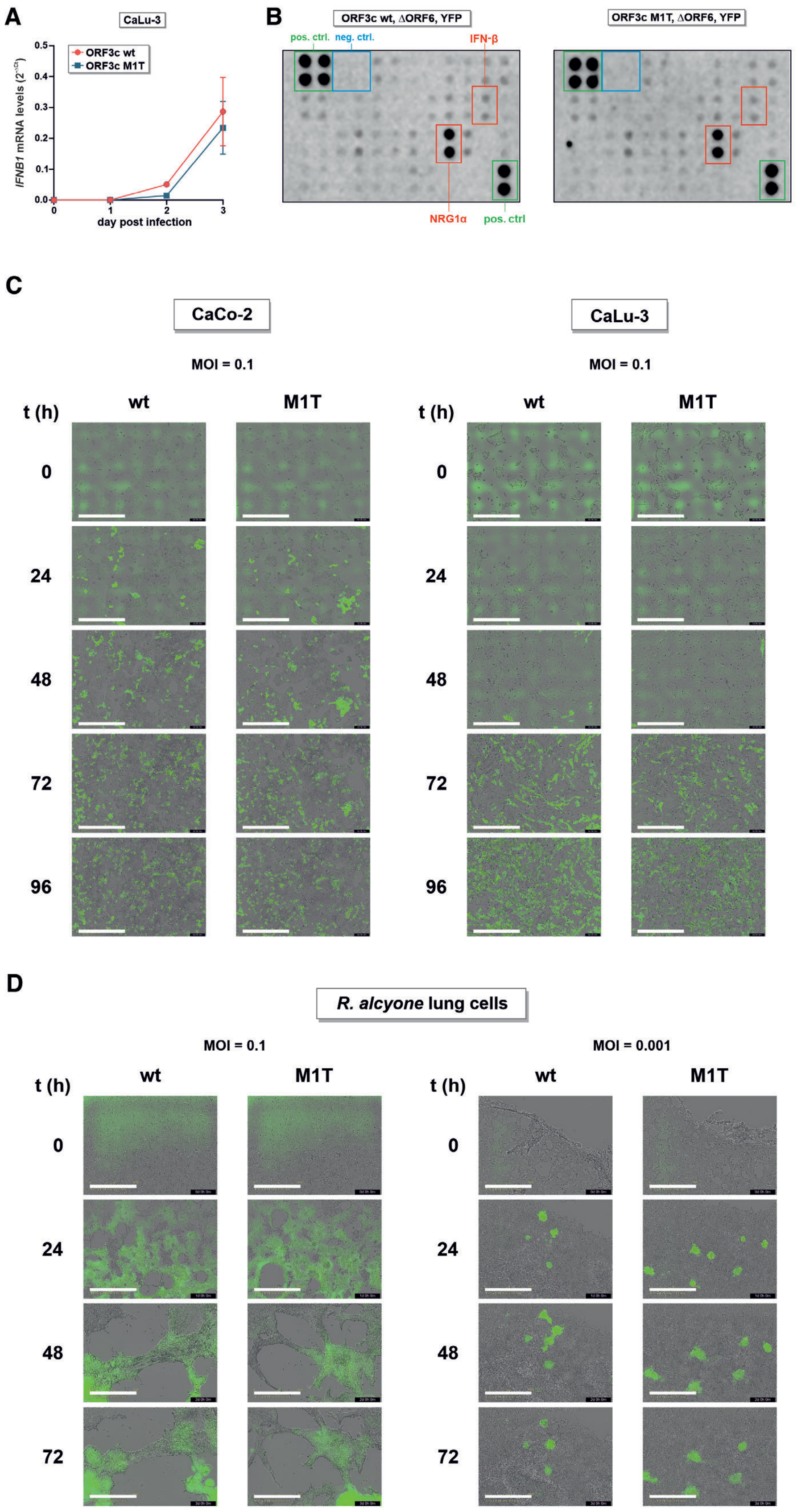

Figure EV5. Cytokine expression and release from cells infected with wild-type or ORF3c-deficient SARS-CoV-2.

A *IFNB1* mRNA levels of the infected CaLu-3 cells in Fig 5A were determined by qPCR. Mean values of four biological replicates ± s.e.m. are shown. *IFNB1* mRNA levels were not or hardly detectable in the respective experiment in CaCo-2 cells (see source data).

B Ninety-six hours post-infection, supernatants of one of the CaLu-3 experiment shown in Fig 5C were harvested and cytokine amounts were quantified using a membrane-based array. This approach enables a semi-quantitative analysis of cytokine release. The signal intensity of each dot indicates the amount of a specific cytokine in the culture supernatant (in duplicates). Positive and negative controls are highlighted in green and blue, respectively. Selected cytokines are highlighted in red. Data are representative of two biological replicates ($n = 2$).

C CaCo-2 and CaLu-3 cells were infected with SARS-CoV-2 ΔORF6-YFP or SARS-CoV-2 ΔORF6-YFP ΔORF3c and analyzed as described in Fig 5C. The images show virus-infected cells (green) over the indicated time points post-infection from one randomly chosen well of one of the replicates of Fig 5C (scale bar = 600 µm).

D *R. alcyone* lung cells stably expressing human ACE2 were infected with SARS-CoV-2 ΔORF6-YFP or SARS-CoV-2 ΔORF6-YFP ΔORF3c and analyzed as described in Fig 5D. The images show virus-infected cells (green) over the indicated time points post-infection from one randomly chosen well of one of the replicates of Fig 5D (scale bar = 600 µm).

Source data are available online for this figure.

© 2023 The Authors
