# Supplementary material for: ORF3c is expressed in SARS‐CoV‐2‐infected cells and inhibits innate sensing by targeting MAVS
Source: EMBO Rep. 2023 Oct 23;24(12):e57137. doi: 10.15252/embr.202357137 (PMC10702836; doi:10.15252/embr.202357137)
Supplement: Supplementary file 2 — PDF+ [file EMBR-24-e57137-s008.pdf]

Article

SOURCE DATA TRANSPARENT PROCESS OPEN ACCESS

EMBO reports

ORF3c is expressed in SARS-CoV-2-infected cells and inhibits innate sensing by targeting MAVS

Martin Müller[1], Alexandra Herrmann[2], Shigeru Fujita[3,4], Keiya Uriu[3,4], Carolin Kruth[1], Adam Strange[3], Jan E Kolberg[1], Markus Schneider[1], Jumpei Ito[3], Marcel A Müller[5], Christian Drosten[5], Armin Ensser[2], The Genotype to Phenotype Japan (G2P-Japan) Consortium, Kei Sato[3,5,6,7,8,9] & Daniel Sauter[1,3,*]

Abstract

Most SARS-CoV-2 proteins are translated from subgenomic RNAs (sgRNAs). While the majority of these sgRNAs are monocistronic, some viral mRNAs encode more than one protein. One example is the *ORF3a* sgRNA that also encodes ORF3c, an enigmatic 41-amino-acid peptide. Here, we show that ORF3c is expressed in SARS-CoV-2-infected cells and suppresses RIG-I- and MDA5-mediated IFN-β induction. ORF3c interacts with the signaling adaptor MAVS, induces its C-terminal cleavage, and inhibits the interaction of RIG-I with MAVS. The immunosuppressive activity of ORF3c is conserved among members of the subgenus sarbecovirus, including SARS-CoV and coronaviruses isolated from bats. Notably, however, the SARS-CoV-2 delta and kappa variants harbor premature stop codons in ORF3c, demonstrating that this reading frame is not essential for efficient viral replication *in vivo* and is likely compensated by other viral proteins. In agreement with this, disruption of ORF3c does not significantly affect SARS-CoV-2 replication in CaCo-2, CaLu-3, or *Rhinolophus alcyone* cells. In summary, we here identify ORF3c as an immune evasion factor of SARS-CoV-2 that suppresses innate sensing in infected cells.

Keywords cryptic open reading frames; IFN antagonist; immune evasion; ORF3c; SARS-CoV-2

Subject Categories Immunology; Microbiology, Virology & Host Pathogen Interaction; Signal Transduction

DOI 10.15252/embr.202357137 | Received 8 March 2023 | Revised 5 October 2023 | Accepted 6 October 2023

EMBO Reports (2023) e57137

Introduction

Since the emergence of the COVID-19 pandemic, all canonical proteins of SARS-CoV-2 have been extensively characterized for their expression, structure, and function. In addition to its prototypical genes, however, SARS-CoV-2 harbors several smaller open reading frames (ORFs) that frequently overlap with other ORFs and may also contribute to efficient viral replication. For example, the ORF3b peptide encoded by *ORF3a* subgenomic RNA (sgRNA) was shown to suppress the induction of type I interferon (IFN) (Konno *et al*, 2020). Intriguingly, naturally occurring variants of ORF3b differ in their immunosuppressive activity and may be responsible for phenotypic differences between SARS-CoV and SARS-CoV-2 (Konno *et al*, 2020). Moreover, several short upstream ORFs (uORFs) have been suggested to regulate translation of downstream genes such as *ORF7b* (Finkel *et al*, 2021). Thus, non-canonical ORFs of SARS-CoV-2 may also be important determinants of viral immune evasion, spread, and/or pathogenicity.

Nevertheless, most of the cryptic ORFs of SARS-CoV-2 remain poorly characterized, and several open questions remain: Do they encode proteins or are they merely a result of selection pressures acting on overlapping reading frames? Do these ORFs exert any regulatory activity, for example, by modulating translation of downstream ORFs via leaky scanning or ribosomal re-initiation? Do they code for functional proteins that contribute to efficient immune evasion and/or replication of SARS-CoV-2? Are the respective peptides or proteins immunogenic?

One interesting cryptic open reading frame is *ORF3c*, located at nt 25457-25579 of the Wuhan-Hu-1 reference genome. This ORF was independently described by different groups and has received

1 Institute for Medical Virology and Epidemiology of Viral Diseases, University Hospital Tübingen, Tübingen, Germany
2 Institute for Clinical and Molecular Virology, University Hospital, Friedrich-Alexander-Universität Erlangen-Nürnberg, Erlangen, Germany
3 Division of Systems Virology, Department of Microbiology and Immunology, The Institute of Medical Science, The University of Tokyo, Tokyo, Japan
4 Graduate School of Medicine, The University of Tokyo, Tokyo, Japan
5 Institute of Virology, Charité – Universitätsmedizin Berlin, Corporate Member of Freie Universität Berlin, Humboldt-Universität zu Berlin, and Berlin Institute of Health, Berlin, Germany
6 International Research Center for Infectious Diseases, The Institute of Medical Science, The University of Tokyo, Tokyo, Japan
7 International Vaccine Design Center, The Institute of Medical Science, The University of Tokyo, Tokyo, Japan
8 Graduate School of Frontier Sciences, The University of Tokyo, Chiba, Japan
9 CREST, Japan Science and Technology Agency, Saitama, Japan
*Corresponding author. Tel: +49 7071 29 80177; E-mail: daniel.sauter@med.uni-tuebingen.de

© 2023 The Authors. Published under the terms of the CC BY 4.0 license.

several alternative names: ORF3c (Firth, 2020; Jungreis *et al*, 2021b), ORF3h (for hypothetical) (Cagliani *et al*, 2020), 3a.iORF1 (Finkel *et al*, 2021), and ORF3b (Pavesi, 2020). Following the homology-based nomenclature proposed by Jungreis and colleagues (Jungreis *et al*, 2021a), we will refer to this open reading frame as *ORF3c* hereafter. Like *ORF3b*, *ORF3c* is one of several open reading frames overlapping with *ORF3a*. *In silico* analyses suggested that the respective ORF3c protein may harbor a transmembrane domain (Firth, 2020) and act as a viroporin (Cagliani *et al*, 2020). Nevertheless, its expression in infected cells, as well as its exact function and relevance for viral replication, have remained unclear.

Here, we show that SARS-CoV-2 ORF3c encodes a stable 41-amino-acid peptide that is expressed in virally infected cells and suppresses the induction of IFN-β expression. Mechanistic analyses revealed that it inhibits innate sensing induced by RIG-I and MDA5, and interacts with the downstream adaptor protein MAVS. Furthermore, it inhibits the interaction of RIG-I with MAVS and induces proteolytic cleavage of MAVS. In line with a relevant function *in vivo*, ORF3c orthologs from different sarbecoviruses share this immunosuppressive activity. However, we also identify SARS-CoV-2 lineages that spread efficiently in the human population despite premature stop codons in their *ORF3c* genes. Furthermore, disruption of *ORF3c* did not affect SARS-CoV-2 replication in CaCo-2, CaLu-3 cells, or bat lung cells. Thus, our findings identify ORF3c as an immune evasion factor that inhibits innate sensing cascades, but is not essential for efficient viral replication.

Results

SARS-CoV-2 *ORF3c* encodes a peptide suppressing IFN-β promoter activation

The *ORF3a* gene of the SARS-CoV-2 reference genome Wuhan-Hu-1 overlaps with several shorter open reading frames that have the potential to encode for peptides of at least 10 amino acids in length (Fig 1A). Translation initiation downstream of the start codon of *ORF3a* may be enabled via non-canonical translation mechanisms, such as leaky scanning, ribosomal shunting, and/or re-initiation (Firth & Brierley, 2012). In line with this, the start codon of ORF3c is part of a strong Kozak sequence, and *in silico* analyses predict ORF3c expression from the ORF3a sgRNA via leaky scanning (Gleason *et al*, 2022) (Fig 1B). Indeed, ribosome profiling and HLA-II immunopeptidome studies suggested that *ORF3c* is translated in SARS-CoV-2-infected cells (Finkel *et al*, 2021; preprint: Weingarten-Gabbay *et al*, 2023). The same study also found evidence for translation of *ORF3d-2* (Finkel *et al*, 2021), which is in agreement with the detection of ORF3d-specific antibodies in sera from previously SARS-CoV-2-infected individuals (Hachim *et al*, 2022). In contrast, we found no evidence of antibodies against ORF3c in SARS-CoV-2 convalescent sera (Fig EV1).

To characterize the stability and potential activity of cryptic ORF3 peptides, we generated expression vectors for the individual peptides harboring a C-terminal HA-tag (without codon optimization). Apart from the *ORF3a* construct, *ORF3c* and *ORF3d/ORF3d-2* code for stable proteins that are readily detectable in transfected cells (Fig 1C). The remaining peptides were not (ORF3b-2, ORF3b-3, and ORF3b-4) or only poorly (ORF3b and ORF3e) detectable.

Since several accessory proteins of SARS-CoV-2 (e.g., ORF3b and ORF6) have been shown to suppress the induction of interferons (Konno *et al*, 2020; Miorin *et al*, 2020; Kimura *et al*, 2021), we hypothesized that some of the cryptic ORF3 peptides may exert similar immune-modulatory activities. Indeed, a luciferase reporter assay revealed that ORF3c significantly suppresses the activation of the IFN-β promoter in response to a constitutively active mutant of the pattern recognition receptor RIG-I (Fig 1D). Notably, ORF3c was also more active than the previously described IFN antagonist ORF3b, which suppressed IFN-β promoter activity only at higher concentrations or upon codon optimization (Konno *et al*, 2020). To test whether ORF3c is able to suppress immune activation upon viral infection, we monitored endogenous *IFNB1* expression upon infection with Sendai virus (SeV), a potent inducer of RIG-I-mediated type I IFN expression (Strahle *et al*, 2006). As expected, SeV induced *IFNB1* expression in a dose-dependent manner (Fig 1E). However, *IFNB1* mRNA levels were reduced by about 50% in the presence of SARS-CoV-2 ORF3c.

Figure 1. Open reading frames in the *ORF3* gene locus and inhibition of IFN-β induction by SARS-CoV-2 ORF3c.

A Genome organization of SARS-CoV-2 is illustrated on top; overlapping ORFs in the ORF3 locus are shown at the bottom. Vertical gray lines indicate internal ATG codons. Experimentally confirmed translation initiation sites (Finkel *et al*, 2021) are highlighted by gray triangles.

B Kozak sequences of the *ORF3c* initiation codon and upstream ATG codons in *ORF3a* are shown. ATG context was determined using TIS predictor (https://www.tispredictor.com/) (Gleason *et al*, 2022).

C Western blot analysis of HEK293T cells transfected with two different concentrations of expression plasmids for the indicated ORF3 proteins and peptides. After 24 h, ORF3a to ORF3e were detected via a C-terminal HA-tag. GAPDH served as loading control. Data are representative of two biological replicates ($n = 2$).

D HEK293T cells were co-transfected with the indicated ORF3 expression plasmids, a reporter plasmid expressing firefly luciferase under the control of the *IFNB1* promoter, and a construct expressing *Gaussia* luciferase under the control of a minimal promoter. To induce immune signaling, half of the samples were additionally co-transfected with an expression plasmid for the CARD domain of RIG-I. One day post-transfection, firefly luciferase activity was determined and normalized to *Gaussia* luciferase activity. Data are shown as mean and s.d. of three biological replicates ($n = 3$) and were analyzed by two-way ANOVA with Sidak's multiple comparison test.

E HEK293T cells were transfected with an expression plasmid for SARS-CoV-2 ORF3c or an empty vector control. Twenty-four hours post-transfection, cells were infected with increasing amounts of Sendai virus (SeV) for an additional 8 h. Cells were lysed to perform either RNA extraction and subsequent qPCR for IFN-β (left panel) or Western blot analysis (right panel). Data on the left are shown as mean and s.d. of three biological replicates ($n = 3$) and were analyzed by two-way ANOVA with Sidak's multiple-comparison test.

F CaCo-2 cells were infected with SARS-CoV-2 wt or SARS-CoV-2 encoding HA-tagged ORF3c at an MOI of 2. 24 and 48 h post-infection, cells were harvested for Western blot analysis. ORF3c expression was detected via the HA-tag. SARS-CoV-2 Nucleocapsid and GAPDH served as controls. Data are representative of two biological replicates ($n = 2$).

Source data are available online for this figure.

▶

 © 2023 The Authors

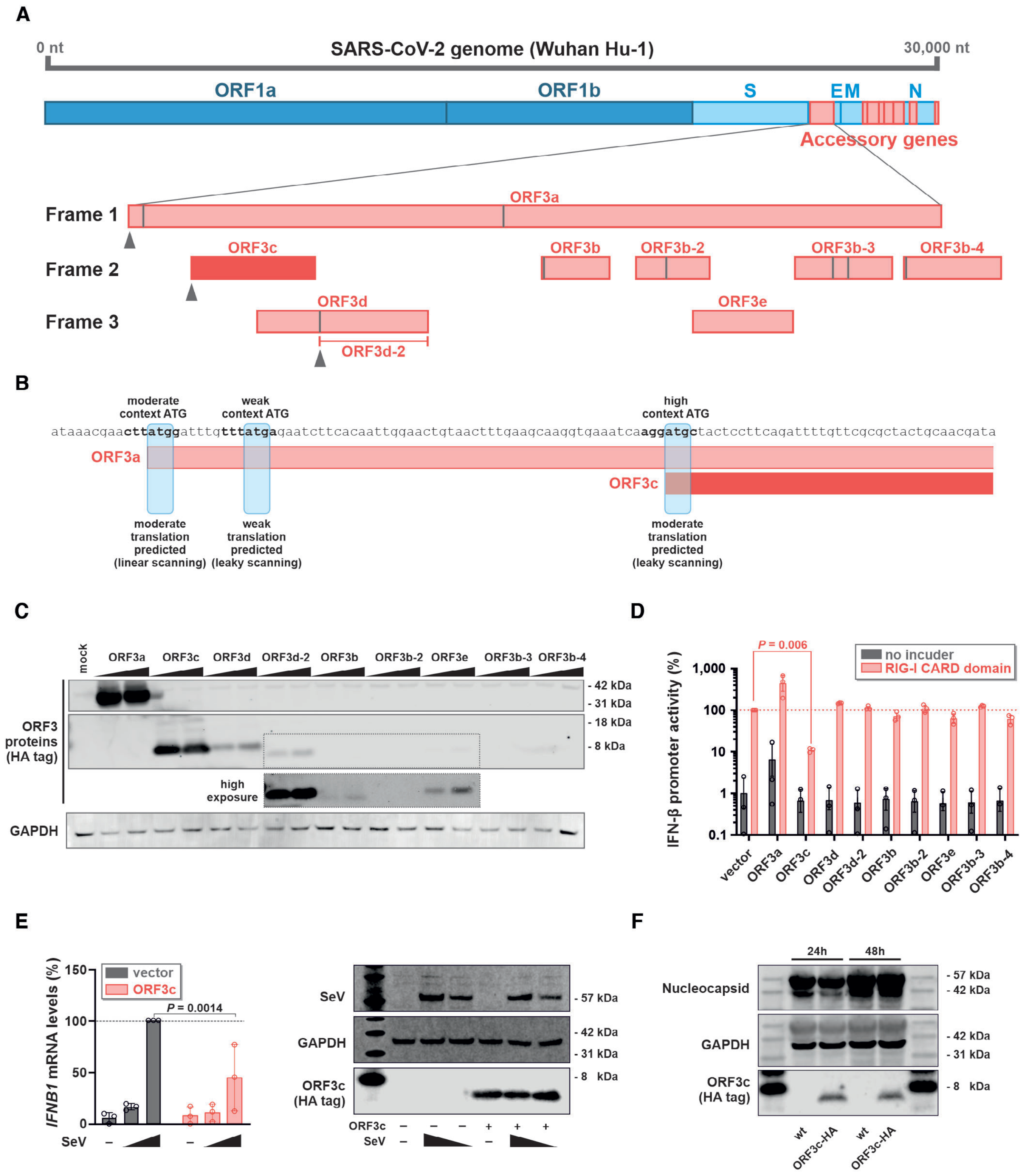

Figure 1.

To definitely prove that ORF3c is produced in SARS-CoV-2-infected cells, we monitored its expression at different time points post-infection via Western blot. Due to the lack of ORF3c-specific antibodies, we used circular polymerase extension reaction (CPER) (Torii *et al*, 2021) to generate a SARS-CoV-2 variant expressing C-terminally HA-tagged ORF3c. Although addition of the HA-tag also

resulted in the insertion of 10 additional amino acids within ORF3a, the rescued virus grew to high titers, similar to the wt virus. Most importantly, ORF3c-HA was readily detectable in CaCo-2 cells, 24 h and 48 h post-infection (Fig 1F). To our knowledge, this is the first direct detection of ORF3c in SARS-CoV-2-infected cells. Together, these findings show that SARS-CoV-2 *ORF3c* encodes a stable peptide that suppresses the production of IFN-β upon viral sensing.

ORF3c targets MAVS and suppresses both RIG-I- and MDA5-mediated immune activation

To elucidate the mechanisms underlying the inhibitory activity of ORF3, we analyzed different steps of the innate RNA sensing cascade culminating in the induction of IFN-β expression (Fig 2A). The *IFNB1* promoter harbors binding sites for both IRF3 and NF-κB. As expected, disruption of the NF-κB-binding site reduced responsiveness to RIG-I-mediated activation (Fig 2B). However, ORF3c still dose dependently reduced promoter activation (Fig 2B), demonstrating that ORF3c does not selectively target NF-κB activation. Next, we activated the sensing cascade at different steps via overexpressing MDA5, MAVS, or a constitutively active mutant of IRF3 in HEK293T cells. While ORF3c suppressed MDA5-mediated immune activation (Fig 2C, left panel), it failed to efficiently suppress *IFNB1* promoter activation in response to MAVS or IRF3 overexpression (Fig 2C, middle and right panels). Together, these findings suggest that ORF3c targets immune activation upstream or at the level of the signaling adaptor MAVS. In line with this, SARS-CoV-2 ORF3c weakly co-immunoprecipitated with MAVS, while we found no evidence for an interaction with RIG-I, MDA5, or TBK1 (Fig 2D). The modest co-immunoprecipitation of ORF3c was also observed when a MAVS mutant lacking its CARD domain was used for pull-down (Fig 2E), demonstrating that this domain is dispensable for the interaction.

MAVS localizes to the outer membrane of mitochondria and mitochondria-associated endoplasmic reticulum membranes (MAMs) (Seth *et al*, 2005; Horner *et al*, 2015). In line with an interaction of ORF3c and MAVS, ORF3c partially co-localizes with a mitochondrial marker (mitochondria-targeted DsRed2) (Fig EV2) (Mozzi *et al*, 2023). Immunofluorescence microscopy revealed no evidence for subcellular re-localization upon SeV infection (Fig EV2). We hypothesized that the interaction of ORF3c with MAVS may also affect the binding of RIG-I to MAVS. Indeed, an established bimolecular fluorophore complementation assay (Sanchez-Aparicio *et al*, 2017) showed evidence for reduced RIG-I:MAVS interaction in the presence of SARS-CoV-2 ORF3c (Fig 2F). Intriguingly, we also noted that increasing amounts of ORF3c resulted in the emergence of a C-terminal MAVS fragment of about 9 kDa (Fig 2G).

Since MAVS-mediated immune signaling culminates in the activation of IRF3 (Fig 2A), ORF3c-mediated targeting is predicted to prevent the activation of this transcription factor. Indeed, ORF3c reduced Ser386 phosphorylation (Fig EV3A) and nuclear translocation (Fig EV3B) of IRF3 in Sendai virus-infected cells, without significantly affecting total cellular levels of IRF3 (Fig EV3C). These findings demonstrate that ORF3c suppresses IRF3-mediated IFN-β expression by binding to MAVS, preventing its interaction with RIG-I, and/or inducing its C-terminal cleavage.

The immunosuppressive activity of ORF3c is conserved among sarbecoviruses

In silico analyses of the ORF3 locus revealed that essentially all sarbecoviruses harbor an *ORF3c* gene encoding a 40- or 41-amino-acid peptide (Firth, 2020). In contrast, the remaining *ORF3* genes are only poorly conserved or vary substantially in their length (Fig 3A). The absence of *ORF3c* from other subgenera of beta-coronaviruses suggests that this open reading frame emerged after the divergence of sarbeco- and hibecoviruses. To test whether the immunosuppressive activity of ORF3c is also conserved, we characterized several orthologs representing human and bat isolates of the SARS-CoV- and SARS-CoV-2-like clusters (Fig 3B). Titration experiments revealed that all ORF3c peptides tested significantly suppress IFN-β promoter activation (Fig 3C). However, ORF3c of SARS-CoV-2

Figure 2. ORF3c interacts with MAVS and inhibits IFN-β induction independently of the pattern recognition receptor.

- A Cartoon illustrating IRF3- and NF-κB-mediated activation of the *IFNB1* promoter upon RIG-I- or MDA5-mediated sensing.
- B HEK293T cells were co-transfected with increasing amounts of an expression plasmid for SARS-CoV-2 ORF3c, a construct expressing *Gaussia* luciferase under the control of a minimal promoter, and a reporter plasmid expressing firefly luciferase under the control of the *IFNB1* promoter (left panel) or a mutant thereof lacking the NF-κB-binding site (right panel). Immune signaling was induced by co-transfecting an expression plasmid for the CARD domain of RIG-I. One day post transfection, firefly luciferase activity was determined and normalized to *Gaussia* luciferase activity. Data are shown as mean and s.d. of three biological replicates ($n = 3$) and were analyzed by one-way ANOVA with Dunnett's multiple-comparison test.
- C HEK293T cells were transfected and analyzed essentially as described in (B). Immune signaling was induced by co-transfecting expression plasmids for MDA5 (left panel), MAVS (central panel) or a constitutively active mutant of IRF3 (right panel). Data are shown as mean and s.d. of three biological replicates ($n = 3$) and were analyzed by one-way ANOVA with Dunnett's multiple-comparison test.
- D, E HEK293T cells were co-transfected with expression plasmids for (D) Flag-tagged RIG-I, MDA5, MAVS, TBK1, (E) MAVS or a mutant thereof lacking its CARD domain (MAVSΔCARD) and an expression plasmid for HA-tagged SARS-CoV-2 ORF3c. One day post-transfection, cells were lysed. Cell lysates were analyzed by Western blotting, either directly ("input") or upon pull-down using a Flag-specific antibody ("IP"). Red arrows indicate faint ORF3c bands. Data are representative of three biological replicates ($n = 3$).
- F HEK293T cells were transfected with plasmids expressing BFP, ORF3c, the C-terminal part of YFP fused to RIG-I and/or the N-terminal part of YFP fused to MAVS. Twenty-four hours later, cells were fixed, and YFP fluorescence was detected by flow cytometry as a reporter for MAVS:RIG-I interaction. Data are shown as mean and s.d. of three biological replicates ($n = 3$) and were analyzed by one-way ANOVA with Dunnett's multiple-comparison test. Exemplary flow cytometry data are shown on the right.
- G HEK293T cells were transfected with increasing amounts of an expression plasmid for SARS-CoV-2 ORF3c. One day post-transfection, cells were lysed for Western blotting. ORF3c was detected via an HA-tag, and MAVS was detected with antiserum specific for the C-terminal part of MAVS. GAPDH served as loading control. Data are representative of three biological replicates ($n = 3$). MAVS bands were quantified and the ratio of the 9 kDa fragment to total MAVS was calculated. Data are presented as mean (±s.e.m.) of three biological replicates ($n = 3$) and were analyzed by one-way ANOVA with Dunnett's multiple-comparison test.

Source data are available online for this figure.

 © 2023 The Authors

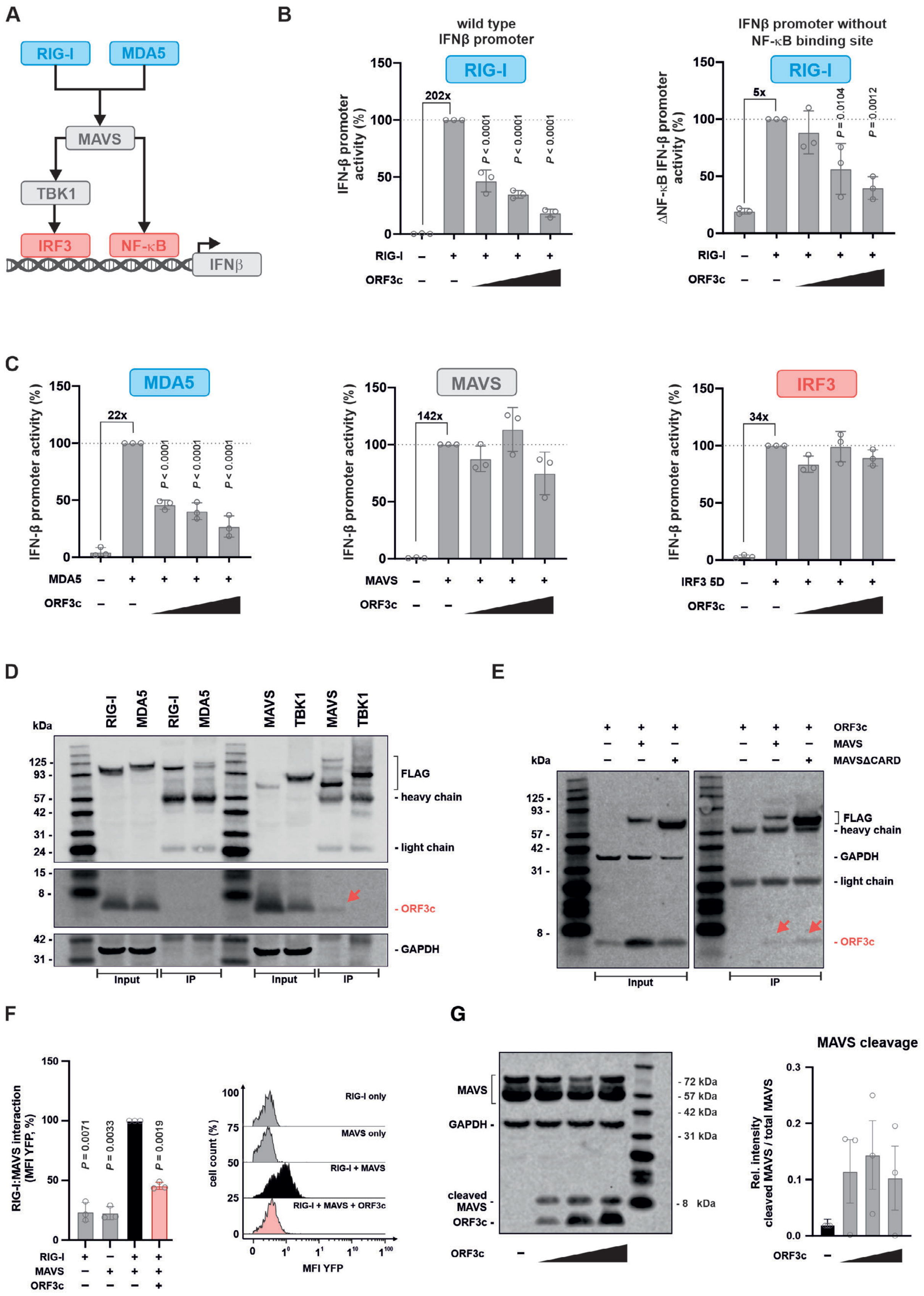
A
RIG-I
MDA5
MAVS
TBK1
IRF3
NF-κB
IFNβ
B
wild type IFNβ promoter
IFNβ promoter without NF-κB binding site
RIG-I
IFN-β promoter activity (%)
ΔNF-κB IFN-β promoter activity (%)
202x
5x
P < 0.0001
P = 0.0104
P = 0.0012
RIG-I
ORF3c
C
MDA5
MAVS
IRF3
22x
142x
34x
IRF3 5D
D
kDa
FLAG
heavy chain
light chain
ORF3c
GAPDH
Input
IP
E
MAVSΔCARD
F
RIG-I:MAVS interaction (MFI YFP, %)
P = 0.0071
P = 0.0033
P = 0.0019
cell count (%)
RIG-I only
MAVS only
RIG-I + MAVS
RIG-I + MAVS + ORF3c
MFI YFP
G
72 kDa
57 kDa
42 kDa
31 kDa
8 kDa
cleaved MAVS
MAVS cleavage
Rel. intensity cleaved MAVS / total MAVS

Figure 2.

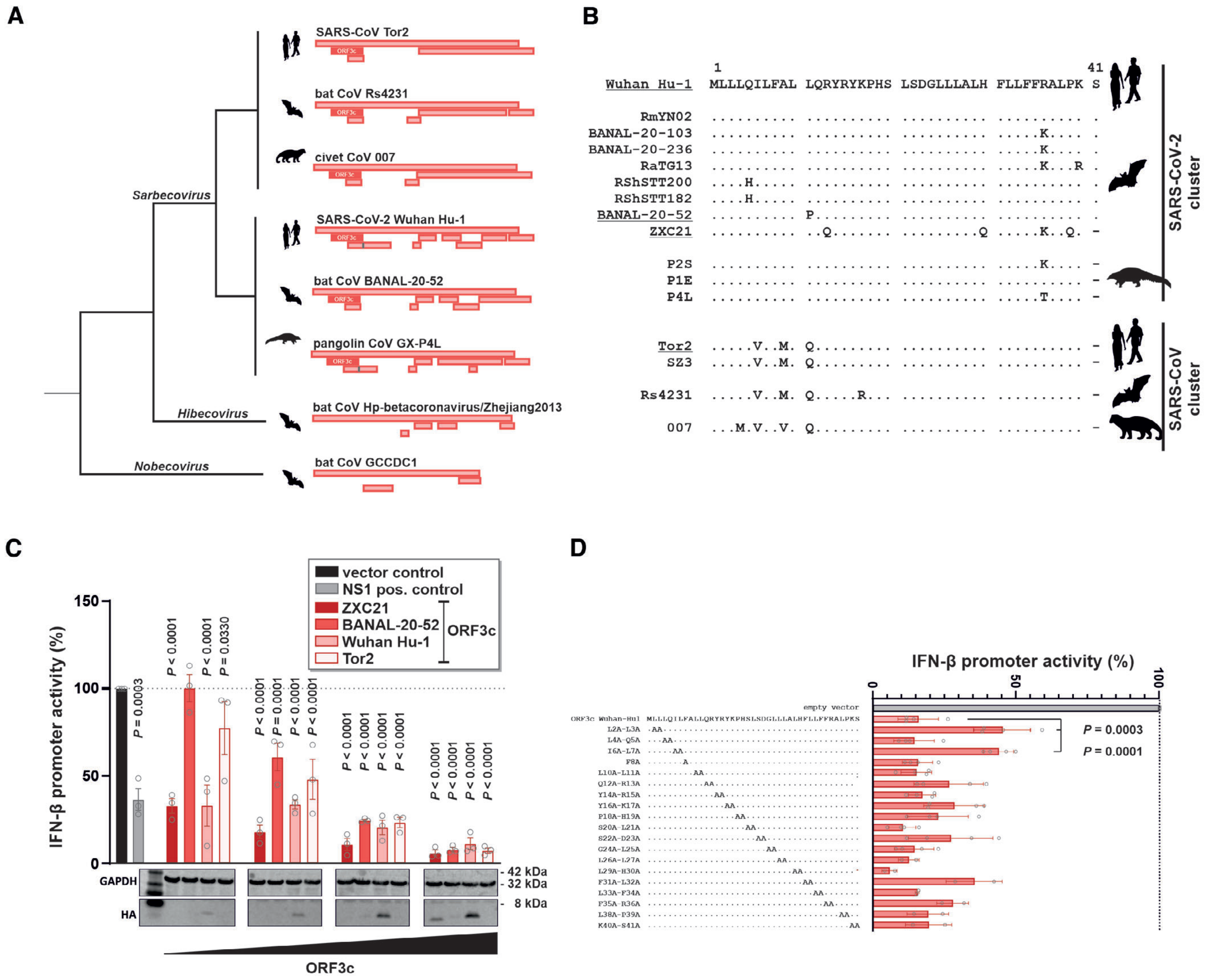

Figure 3. Conservation of ORF3c and its immunosuppressive activity in sarbecoviruses.

A Simplified cartoon illustrating the *ORF3* locus of randomly selected members of the *Sarbeco-*, *Hibeco-* and *Nobecovirus* genera. Open reading frames with a length of at least 30 nucleotides are indicated as rectangles. ORF3c is highlighted in dark red.

B Alignment of ORF3c amino acid sequences of the indicated viral isolates. Members of the SARS-CoV-2 cluster are shown on top, and members of the SARS-CoV cluster are at the bottom. For the underlined ORF3c sequences, expression plasmids were generated and analyzed for their ability to inhibit *IFNB1* promoter activation in (C).

C HEK293T cells were co-transfected with increasing amounts of the indicated ORF3c expression plasmids, a reporter plasmid expressing firefly luciferase under the control of the *IFNB1* promoter and a construct expressing *Gaussia* luciferase under the control of a minimal promoter. An expression plasmid for Influenza A virus non-structural protein 1 (NS1, gray) served as positive control. Immune signaling was induced by co-transfecting an expression plasmid for the CARD domain of RIG-I. One day post-transfection, firefly luciferase activity was determined and normalized to *Gaussia* luciferase activity (top panel). ORF3c expression was monitored by Western blotting (bottom panel). Data are shown as mean and s.d. of three biological replicates ($n = 3$) and were analyzed by two-way ANOVA with Sidak's multiple-comparison test.

D HEK293T cells were co-transfected with expression plasmids and analyzed essentially as described in (C). The respective alanine mutations are indicated in the alignment on the left. Data are shown as mean and s.d. of between two and four biological replicates ($n = 2$ or 4) and were analyzed by one-way ANOVA with Dunnett's multiple-comparison test.

Source data are available online for this figure.

Wuhan Hu-1 and a closely related bat coronavirus (ZXC21) suppressed IFN-β promoter activation more efficiently than ORF3c of SARS-CoV Tor2 and the SARS-CoV-2-like bat coronavirus BANAL-20-52, possibly because of differences in overall ORF3c protein levels (Fig 3C). We also analyzed these four ORF3c orthologs for their ability to interact with MAVS (Fig EV4A). While we confirmed the interaction of SARS-CoV-2 Wuhan Hu-1 ORF3c with MAVS, the less efficient detection of the remaining three ORF3c orthologs prevents a direct comparison and any meaningful conclusions. Notably, the HA-tagged ORF3c orthologs of Wuhan Hu-1,

 © 2023 The Authors

BANAL-20-52, and ZXC21, but not Tor2, were also detectable by flow cytometry in transfected HEK293T cells (Fig EV4B). We therefore tested Wuhan Hu-1, BANAL-20-52 and ZXC21 ORF3c for their ability to interfere with RIG-I:MAVS interaction using the fluorophore complementation assay described above (Fig 2F). In line with a conserved mode of action, all three ORF3c orthologs suppressed RIG-I:MAVS interaction to a similar extent (Fig EV4C).

To further map residues in ORF3c involved in its immunosuppressive activity, we performed an alanine scan using the Wuhan Hu-1 ortholog (Fig 3D). While all ORF3c mutants tested still reduced IFN-β promoter activity, the double mutations L2A/L3A and I6A/L7A significantly reduced the immunosuppressive effect of SARS-CoV-2 ORF3c. Notably, these mutants also lost their ability to interact with ORF3c, although they were efficiently expressed (Fig EV4A). In summary, these results identify residues in the N-terminus of ORF3c that contribute to its inhibitory activity and demonstrate that ORF3c orthologs from different sarbecoviruses suppress the induction of IFN-β, albeit to different degrees.

A natural R36I polymorphism does not affect the immunosuppressive activity of ORF3c

Since the emergence of SARS-CoV-2 in 2019, several mutations have occurred throughout the viral genome. One notable mutation is G25563T (Fig 4A), which is found in the beta, eta, iota, and mu variants and has been associated with increased transmission fitness (Oulas *et al*, 2021). Although G25563T simultaneously introduces non-synonymous mutations in ORF3a (Q57H), ORF3c (R36I), and ORF3d (E31*) (Fig 4A), previous studies focused only on possible phenotypic consequences of the Q57H change in ORF3a. Notably, however, the R36I mutation in ORF3c is predicted to result in a conformational change (Fig 4B) and a transmembrane domain in the C-terminal half of ORF3c (Fig 4C). We therefore analyzed whether the R36I change may affect the subcellular localization and/or immune-suppressive activity of SARS-CoV-2 ORF3c. Immunofluorescence microscopy revealed that ORF3c of the Wuhan-Hu-1 reference strain and an R36I mutant thereof are similarly distributed throughout the cytoplasm (Fig 4D). Moreover, both ORF3c variants dose dependently suppressed RIG-I-mediated IFN-β promoter activation to a similar extent (Fig 4E). Thus, the G25563T polymorphism of the SARS-CoV-2 beta, eta, iota and mu variants does not seem to alter the IFN-suppressive activity of ORF3c.

Some SARS-CoV-2 variants harbor premature stop codons in *ORF3c*

To better understand the relevance of an intact *ORF3c* gene for viral spread, we screened the GISAID SARS-CoV-2 sequence repository (Khare *et al*, 2021) for PANGO (sub)lineages harboring a premature ORF3c stop codon in at least 20% of the isolates. We identified two mutations fulfilling these criteria: the first one (C25469T) introduces a premature stop codon (Q5*) in ORF3c and an S26L change in ORF3a (Fig 4F). It is present in about 44% of B.1.617 isolates and in almost all sequences of the B.1.617.1 (delta) and B.1.617.2 (kappa) sublineages (Fig 4G). The second mutation (del25498-25530) represents an in-frame deletion in ORF3a and can be found in about 81% of all B.1.630 isolates. This deletion results in the loss of the initiation codon of ORF3d and a premature stop codon (Y14*) in ORF3c. The presence of premature ORF3c stop codons in a substantial fraction of B.1.617 and B.1.630 lineages suggests that ORF3c is dispensable for efficient IFN-β suppression *in vivo* and/or may be compensated by changes elsewhere in the genome. Notably, however, the analysis of GISAID SARS-CoV-2 sequences also revealed that a subset (~3%) of B.1.617.2 viruses had acquired an additional point mutation that reverted the stop codon at position 5 to a tyrosine (*5Y), thereby reconstituting ORF3c.

ORF3c is dispensable for efficient SARS-CoV-2 replication

To assess the importance of *ORF3c* for efficient viral replication, we generated SARS-CoV-2 Wuhan Hu-1 variants harboring inactivating mutations in this gene. Introducing premature stop codons into *ORF3c* is not possible without simultaneously introducing non-synonymous mutations in ORF3a and/or ORF3d. We therefore mutated the start codon of ORF3c to threonine (M1T), which resulted in a silent mutation in the overlapping *ORF3a* gene (D22D) (Fig 5A, left panel). After rescue and validating successful introduction of the mutation, we infected CaCo-2 and CaLu-3 cells with an MOI of 0.1. Quantification of SARS-CoV-2 RNA copies in the culture supernatants over a period of 3 days revealed that ORF3c M1T replicated as efficiently as wild-type SARS-CoV-2 (Fig 5A, right panels). Furthermore, *IFNB1* mRNA levels were very low or undetectable in infected CaCo-2 cells, and no significant changes in *IFNB1* induction were observed between CaLu-3 cells infected with wild-type and ORF3c-deficient SARS-CoV-2 (Fig EV5A). In a parallel experiment,

◀ **Figure 4. Characterization of naturally occurring variants of SARS-CoV-2 ORF3c.**

A Cartoon illustrating non-synonymous changes in ORF3a, c and d as a result of the naturally occurring polymorphism G25563T.
B Secondary structure of Wuhan Hu-1 ORF3c (red) and the respective R36I variant thereof (blue) as predicted using PEP-FOLD 3 (Camproux *et al*, 2004).
C The presence of transmembrane domains in Wuhan Hu-1 ORF3c (left panel) and the respective R36I variant thereof (right panel) was predicted using TMHMM – 2.0 (Krogh *et al*, 2001).
D Representative images of HEK293T cells transfected with expression plasmids for Wuhan Hu-1 ORF3c or ORF3c R36I. One day post-transfection, cells were stained for ORF3c (anti-HA, green) and nuclei (DAPI, blue) (scale bar = 20 μm).
E HEK293T cells were co-transfected with increasing amounts of the indicated ORF3c expression plasmids, a reporter plasmid expressing firefly luciferase under the control of the *IFNB1* promoter and a construct expressing *Gaussia* luciferase under the control of a minimal promoter. Immune signaling was induced by co-transfecting an expression plasmid for the CARD domain of RIG-I. One day post-transfection, firefly luciferase activity was determined and normalized to *Gaussia* luciferase activity. Data are shown as mean and s.d. of three biological replicates ($n = 3$) and were analyzed by two-way ANOVA with Sidak's multiple-comparison test.
F Mutations introducing premature stop codons in ORF3c that can be found in at least 20% of the samples of at least one PANGO (sub)lineage.
G Frequency of the mutations shown in (F) in the PANGO (sub)lineages B.1.617.1 (delta), B.1.617.2 (kappa), B.1.617 and B.1.630.

Source data are available online for this figure.

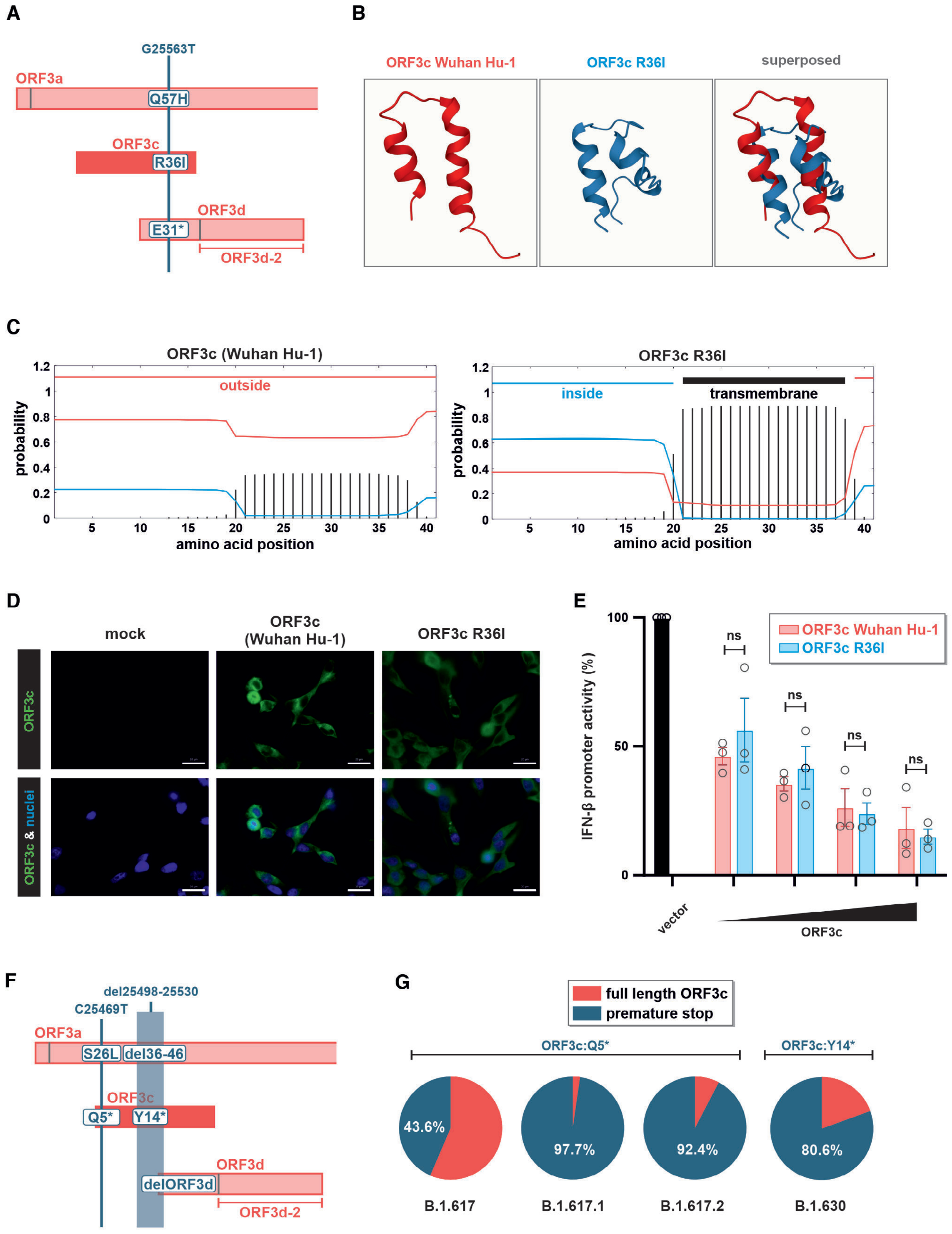

Figure 4.

© 2023 The Authors

we introduced a premature stop codon (Q5*) in ORF3c, mimicking the mutation that can naturally be found in the SARS-CoV-2 delta and kappa variants (Figs 4F and 5B, left panel). Since the respective nucleotide change introduces a S26L mutation in ORF3a, we simultaneously disrupted *ORF3a* by introducing a premature stop codon (R6*) downstream of a methionine and potential alternative start codon at position 5. While loss of ORF3a markedly reduced replicative fitness in CaCo-2 cells, the virus still replicated efficiently in CaLu-3 cells (Fig 5B, right panels). As observed for the ORF3c M1T mutant, introduction of ORF3c Q5* did not significantly affect the replicative fitness of the virus.

Since several (accessory) proteins of SARS-CoV-2 have been shown to suppress the induction of IFN-β and/or IFN-β-mediated immune activation (Xia *et al*, 2020; Min *et al*, 2021), we hypothesized that the immunosuppressive effect of ORF3c may be masked by other viral factors. One potent suppressor of IRF3-mediated IFN-β induction is ORF6 (Li *et al*, 2020; Kimura *et al*, 2021). We therefore generated a BAC clone of SARS-CoV-2, in which *ORF6* was replaced by a *YFP* reporter gene, and introduced the ORF3c M1T mutation described above (Fig 5C, left panel). This clone is based on the B.1 variant, and therefore additionally harbors the ORF3c R36I mutation described above. The viral spread was monitored by live-cell imaging and quantification of YFP-expressing (i.e. infected) cells. Again, disruption of *ORF3c* did not result in impaired viral spread, and the ORF3c M1T virus replicated as efficiently as its ORF3c wild-type counterpart (Fig 5C, right panels, Fig EV5C). Furthermore, release of 40 different cytokines (including IFN-β) was not markedly affected by the loss of ORF3c (Fig EV5B). Finally, we also performed experiments in bat cells to identify potential species-specific effects. We took advantage of an immortalized lung cell line derived from *Rhinolophus alcyone*, stably expressing human ACE2. Similar to the results obtained in human cells, loss of ORF3c did not significantly affect infection rates in these bat cells (Figs 5D and EV5D).

Thus, while ORF3c and its immunosuppressive activity are conserved among sarbecoviruses, the respective gene is dispensable for infection and/or replication in CaCo-2, CaLu-3, and *R. alcyone* lung cells.

Discussion

Several lines of evidence suggested that the *ORF3c* gene of SARS-CoV-2 codes for a peptide that plays a role in viral replication: (i) the respective open reading frame is conserved in different sarbecoviruses and shows synonymous site conservation (Firth, 2020; Nelson *et al*, 2020; Jungreis *et al*, 2021b) (Fig 3), (ii) upstream ATGs do not show a strong initiation context and may allow ORF3c translation via leaky scanning (Firth, 2020) (Fig 1), (iii) ribosomal profiling and the HLA-II immunopeptidome suggested that ORF3c is translated in SARS-CoV-2-infected cells (Finkel *et al*, 2021; preprint: Weingarten-Gabbay *et al*, 2023), (iv) ORF3c shows a high density of $CD8^+$ T-cell epitopes (Nelson *et al*, 2020), and (v) *in silico* analyses predict a conserved transmembrane domain in ORF3c and a potential role as viroporin (Cagliani *et al*, 2020; Firth, 2020). Still, the expression of ORF3c in infected cells, its exact function and its contribution to efficient viral replication have remained unclear.

Here, we identify ORF3c as an immune evasion factor of SARS-CoV-2 and other sarbecoviruses that inhibits IRF3-mediated induction of IFN-β upon activation of innate sensing cascades. The generation of a replication-competent SARS-CoV-2 variant encoding HA-tagged ORF3c allowed us to demonstrate the presence of a stable ORF3c peptide in virus-infected cells. Using luciferase reporter assays, we show that ORF3c suppresses activation of the *IFNB1* promoter by the pattern recognition receptors (PRRs) and RNA sensors RIG-I and MDA5. Importantly, ORF3c also significantly reduced the expression of endogenous IFN-β in response to Sendai virus infection, a known inducer of RIG-I sensing (Strahle *et al*, 2006). Since ORF3c inhibits RIG-I- and MDA5-mediated immune activation, it most likely targets a factor further downstream in the signaling cascade. In line with this, co-immunoprecipitation experiments revealed an interaction of ORF3c with the mitochondrial signaling adaptor MAVS. We found no evidence of an interaction with other components of the sensing cascade (i.e., RIG-I, MDA5, or TBK1). Notably, ORF3c failed to prevent *IFNB1* promoter activation if MAVS itself was used as an activator. This lack of inhibition is not merely the result of a saturation effect since MAVS induced the *IFNB1* promoter less efficiently than RIG-I (Fig 2). MAVS is targeted by proteins from different viruses. For example, Influenza A Virus PB1-F2 inhibits innate sensing by binding to MAVS (Varga *et al*, 2011) and decreasing the mitochondrial membrane potential (Varga *et al*, 2012). Another example is the NS3/4A serine protease of Hepatitis C virus (HCV), which cleaves MAVS, thereby inhibiting downstream immune activation (Anggakusuma *et al*, 2016). Similarly, SARS-CoV-2 ORF10 was recently shown to induce the degradation of MAVS via mitophagy (Li *et al*, 2022). Further mechanistic analyses revealed that ORF3c induces the proteolytic processing of MAVS,

◀ **Figure 5. Disruption of ORF3c does not affect SARS-CoV-2 infection and/or replication in CaCo-2, CaLu-3 or *R. alcyone* cells.**

A CPER was used to disrupt the start codon of ORF3c (M1T) in SARS-CoV-2 without affecting the amino acid sequence of ORF3a (left panel). CaCo-2 and CaLu-3 cells were infected with ORF3c wild-type (red) or ORF3c-mutated (blue) SARS-CoV-2 at an MOI of 0.1. Viral replication was monitored over 72 h by determining viral RNA copies in the culture supernatants (right panels). Data are shown as mean and s.d. of four (*n* = 4, CaCo-2) or eight (*n* = 8, CaLu-3) biological replicates.

B CPER was used to introduce a premature stop codon in ORF3c (Q5stop). To avoid any bias by simultaneously changing the protein sequence of ORF3a (S26L), a premature stop codon was also inserted in ORF3a (R6*) (left panel). Viral replication (right panels) was monitored in CaCo-2 and CaLu-3 cells as described in (A). Data are shown as mean and s.d. of eight (*n* = 8) biological replicates.

C A SARS-CoV-2 BAC clone harboring a disrupted ORF3c (M1T) and expressing YFP instead of ORF6 was generated (left panel). CaCo-2 and CaLu-3 cells were infected with SARS-CoV-2 ΔORF6-YFP (red) or SARS-CoV-2 ΔORF6-YFP ΔORF3c (blue) at an MOI of 0.1. Cells were placed in a live cell imaging device, and the area of YFP-positive cells over the total area of cells was quantified every 4 h for 96 h. Data are shown as mean and s.d. of three (*n* = 3) biological replicates.

D *R. alcyone* lung cells stably expressing human ACE2 were infected with SARS-CoV-2 ΔORF6-YFP (red) or SARS-CoV-2 ΔORF6-YFP ΔORF3c (blue) at an MOI of 0.1 (left panel) or 0.001 (right panel). Cells were placed in a live cell imaging device, and the area of YFP-positive cells over the total area of cells was quantified every 4 h. Data are shown as mean and s.d. of two (*n* = 2, MOI 0.001) to three (*n* = 3, MOI 0.1) biological replicates.

Source data are available online for this figure.

© 2023 The Authors

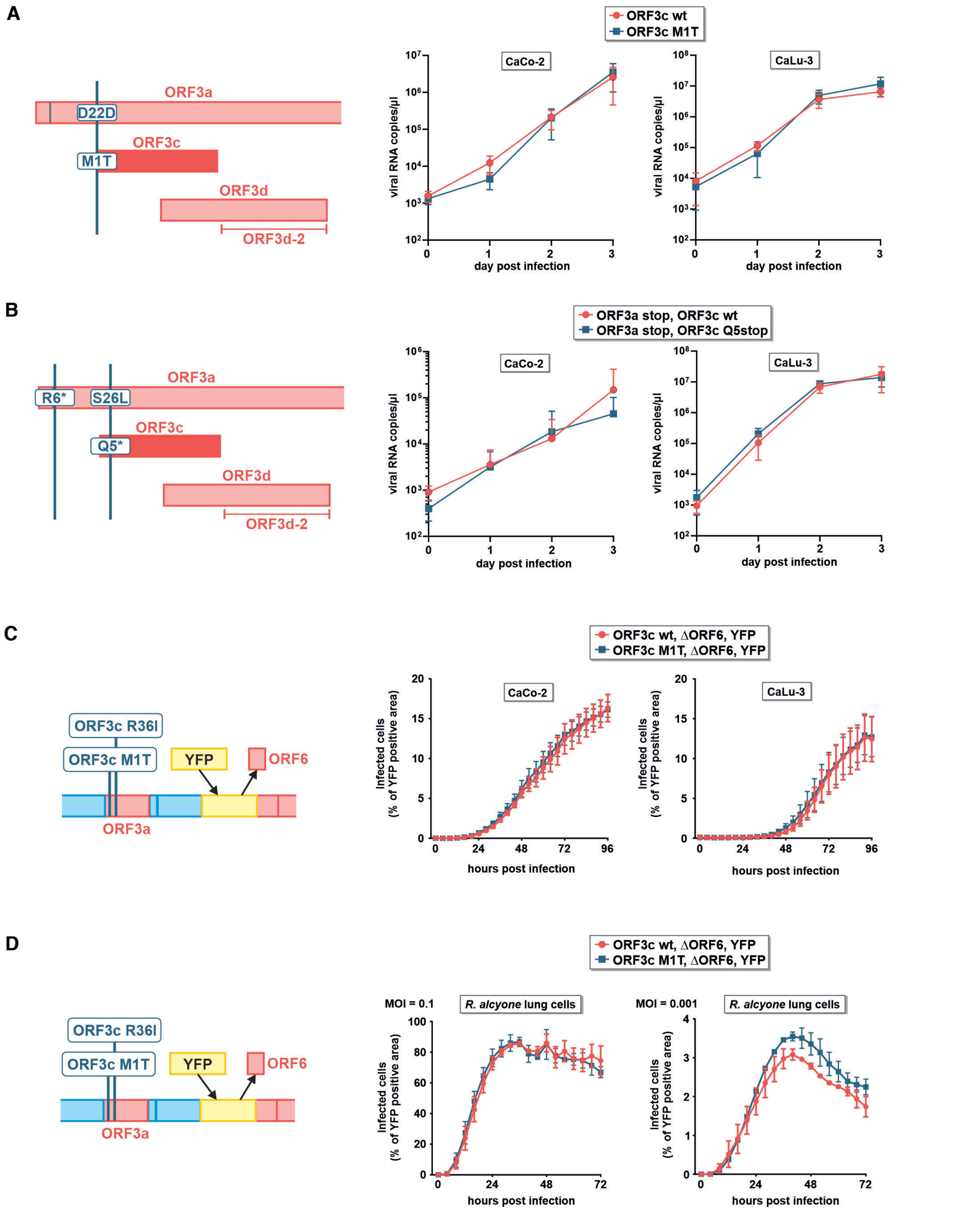

Figure 5.

© 2023 The Authors

resulting in a C-terminal 9 kDa fragment of this signaling protein (Fig 2G). This suggests that ORF3c may inactivate MAVS by removing its C-terminal transmembrane domain. Furthermore, ORF3c reduced the interaction of MAVS with RIG-I (Fig 2F). Thus, one possible mode of action is a competitive binding of ORF3c and RIG-I (and possible MDA5) to MAVS. In the presence of ORF3c, the CARD domain of MAVS may not be accessible and thus not be bound and activated by active RIG-I or MDA5. Notably, however, co-immunoprecipitation experiments showed that the CARD domain of MAVS is dispensable for an interaction of ORF3c with MAVS (Fig 2E). A competitive binding of ORF3c and RIG-I to MAVS may also explain why ORF3c fails to suppress IFN-β promoter activation when MAVS overexpression is used as an inducer (Fig 2C). We hypothesize that exogenous MAVS may saturate the inhibitory activity of ORF3c and compensate for endogenous MAVS that is bound and blocked by ORF3c.

In line with the relevant role of ORF3c in viral replication, its immunosuppressive activity is conserved in orthologs of other sarbecovirus species, including the SARS-CoV reference virus Tor2. While all orthologs tested inhibited *IFNB1* promoter activation, those of SARS-CoV Tor2 and batCoV BANAL-20-52 were less active (and expressed to lower levels) than their counterparts from SARS-CoV-2 Wuhan-Hu-1 and batCoV ZXC21. The reduced activity and expression/detection of BANAL-20-52 ORF3c can be ascribed to a single amino acid change (L11P) distinguishing it from Wuhan-Hu-1 ORF3c (Fig 3B). While L11 is largely conserved in the SARS-CoV-2 cluster, most of the viruses in the SARS-CoV cluster (including Tor2) harbor a glutamine at this position (Fig 3B) (Cagliani *et al*, 2020; Firth, 2020). Thus, polymorphisms at position 11 can affect the inhibitory activity of ORF3c. In addition to this, our alanine scanning approach revealed that Leu2/Leu3 and Ile6/Leu7 are also contributing to the immunosuppressive effect of ORF3c and its ability to interact with MAVS (Fig 3D). Most of these residues are conserved among different sarbecoviruses. One notable exception is Ile6 (Fig 3B). Viruses from the SARS-CoV cluster harbor a Valine at this residue. These include SARS-CoV Tor2 ORF3c, which was less active than its SARS-CoV-2 counterpart.

ORF3c is not the only SARS-CoV-2 protein that interferes with RIG-I- and/or MDA-5-mediated immune activation. As already mentioned above, ORF10 suppresses innate sensing by targeting MAVS (Li *et al*, 2022). Moreover, ORF3b, nucleocapsid, ORF6, and ORF8 have all been shown to suppress IFN-β expression (Kopecky-Bromberg *et al*, 2007; Konno *et al*, 2020; Li *et al*, 2020; Hayn *et al*, 2021; Kimura *et al*, 2021), highlighting the selection pressure exerted by this pathway. Intriguingly, SARS-CoV-2 has recently also been shown to harbor small open reading frames in its negative-sense RNA (rORFs), whose gene products suppress IFN-β promoter activity (Gong *et al*, 2023). The convergent evolution of viral proteins exerting overlapping immune evasion activities may represent a backup mechanism that allows viral replication even if one of the IFN-β-suppressing proteins is lost. In line with this, SARS-CoV-2 variants expressing a C-terminally truncated, inactive ORF6 protein have emerged several times during the pandemic and spread via human-to-human transmission (Kimura *et al*, 2021). Similarly, natural SARS-CoV-2 variants lacking an intact ORF3c gene still efficiently spread in the human population. In fact, more than 80% of the sequenced genomes of B.1.617.1, B.1.617.2, and B.1.630 harbor premature stop codons at positions 5 and 14, respectively (Fig 4). We hypothesized that the loss of ORF3c in these viruses may be compensated by the activity of ORF6. However, replication kinetics in CaCo-2, CaLu-3, and bat cells revealed that loss of ORF3c does not affect viral replication in the absence of ORF6 either (Fig 5). Intriguingly, IFN-β mRNA levels were not increased upon infection with the SARS-CoV-2 double mutant lacking ORF3c and ORF6 (Fig EV5B). Thus, yet another viral inhibitor of IFN-β expression (e.g., ORF8 or N) may be able to rescue efficient viral replication in this case.

Notably, the emergence of premature stop codons in small reading frames such as *ORF3c* may also be tolerated or even be beneficial if they provide a fitness advantage by optimizing overlapping reading frames. For example, the ORF3c Q5* mutation is accompanied by an S26L change in ORF3a. However, experimental disruption of ORF3c without changing the amino acid sequence of ORF3a (Fig 5A) or upon deletion of ORF3a (Fig 5B) did not affect viral replication *in vitro* either.

Thus, it remains to be determined whether an intact ORF3c directly confers a significant fitness advantage to the virus *in vivo* that is not apparent in infected cell lines. Future experiments in animal models will shed light on the impact of ORF3c on immune activation and viral replication in an infected organism. We encourage performing *in vivo* infection experiments with SARS-CoV-2 variants harboring artificial or naturally occurring mutations in *ORF3c*, either alone or in combination with mutations in other immune evasion genes. While experiments in small animal models such as mice or hamsters can certainly provide important insights into the role of ORF3c, they may not necessarily reflect the situation in naturally infected host or reservoir species. Thus, it will also be important to further monitor the evolution of *ORF3c* variants in naturally infected hosts, potential co-evolution with other viral genes, and possible associations of *ORF3c* variants with viral loads, disease progression, or transmissibility.

One intriguing observation pointing to a selection advantage of ORF3c *in vivo* is the emergence of a nucleotide change in a subfraction (~3%) of B.1.617.2 viruses reverting their ORF3c stop codon at position 5 to a tyrosine (*5Y). This is different from a naturally occurring truncation of ORF8 that is not reverted and results in two shorter open reading frames (ORF8a and ORF8b) with signatures of purifying selection (Bykova *et al*, 2023). Thus, it is tempting to speculate that the loss of ORF3c was initially just carried along with mutations elsewhere in the genome (e.g., in Spike) that conferred a major fitness advantage to the virus before beneficial ORF3c expression was reverted by another point mutation.

In summary, our study identifies ORF3c as an immune evasion factor of SARS-CoV-2 and other sarbecoviruses. While an intact ORF3c gene is clearly dispensable for viral replication *in vitro* and *in vivo*, the conservation of this short open reading frame and the pseudo-reversion of premature stop codons suggest that it may still contribute to efficient viral replication *in vivo*. The emergence of future SARS-CoV-2 variants and *in vivo* infection experiments may help to fully decipher the role of this enigmatic ORF and its co-evolution with other viral genes.

Materials and Methods

Cell lines

HEK293T (CVCL_0063) were maintained in Dulbecco's modified Eagle medium (DMEM) supplemented with 10% heat-inactivated

© 2023 The Authors

fetal calf serum (FCS), L-glutamine (2 mM), streptomycin (100 mg/ml), and penicillin (100 U/ml) and were cultured at 37°C, 90% humidity and 5% CO_2. They were isolated from a female fetus. HEK293T cells were transfected using a standard calcium phosphate method. HEK293-C34 cells, IFNAR1 KO HEK293 cells expressing human ACE2 and TMPRSS2 by doxycycline treatment (Torii *et al*, 2021), were maintained in DMEM (high glucose) containing 10% FBS, 10 mg/ml blasticidin (InvivoGen), and 1% PS. CaCo-2 (human colorectal adenocarcinoma, male, CVCL_0025) cells were grown in Dulbecco's modified Eagle's medium (DMEM) supplemented with 10% heat-inactivated FCS, 100 units/ml penicillin, 100 µg/ml streptomycin, 2 mM L-glutamine, and 1× non-essential amino acids (NEAA). CaLu-3 cells (lung adenocarcinoma, male, CVCL_0609) were cultured in Eagle's minimum essential medium (EMEM) supplemented with 20% heat-inactivated FCS, 100 units/ml penicillin, and 100 µg/ml streptomycin. Medium was changed every day. Vero E6 (Cercopithecus aethiops-derived epithelial kidney, female, CVCL_0574) cells were grown in Dulbecco's modified Eagle's medium (DMEM) supplemented with 2.5% heat-inactivated FCS, 100 units/ml penicillin, 100 µg/ml streptomycin, 2 mM L-glutamine, 1 mM sodium pyruvate, and 1× non-essential amino acids. Derivatives thereof stably expressing TMPRSS2 (CVCL_YQ49) were cultured in DMEM supplemented with 5% heat-inactivated FCS, 100 units/ml penicillin, 100 µg/ml streptomycin, and 100 µg/ml G418. The generation of a clonal immortalized lung cell line from *Rhinolophus alcyone* stably expressing human ACE2 (RhiLu-hACE2) has been described before (Muth *et al*, 2018). Instead of the clonal cell line described in Muth *et al* (2018), polyclonal precursor cells were used in this study. The bat cells were cultured in DMEM supplemented with 10% Endotoxin-free, heat-inactivated fetal calf serum (FCS), L-glutamine (2 mM), streptomycin (100 mg/ml) and penicillin (100 U/ml). Cells were not authenticated, but regularly tested for mycoplasma contamination.

Virus strains and virus propagation

SARS-CoV-2 ΔORF6-YFP, SARS-CoV-2 ΔORF6-YFP ORF3c-HA, and SARS-CoV-2 ΔORF6-YFP ΔORF3c were propagated by inoculation of Vero E6/TMPRSS2 cells in 75 cm^2 cell culture flasks in medium containing 2% FCS. Cells were incubated at 37°C and supernatants were harvested 2–4 days post-inoculation. Supernatants were centrifuged for 5 min at 1,000 *g* to remove cellular debris, and then aliquoted and stored at −80°C as virus stocks. Infectious titer was determined in Vero E6/TMPRSS2 cells as Tissue Culture Infection Dose 50 (TCID50)/ml. All experiments with infectious SARS-CoV-2 were performed under BSL3 conditions.

In silico prediction of translation initiation sites

Translation initiation sites (TIS) and Kozak context were determined using TIS predictor (https://www.tispredictor.com/) (Gleason *et al*, 2022).

In silico prediction of ORF3c secondary structure and transmembrane domains

To predict the secondary structure of ORF3c and its R36I variant, PEP-FOLD 3 (Camproux *et al*, 2004) was used. The effect of point mutations on a transmembrane domain in ORF3c was predicted using the "Prediction of transmembrane helices in proteins" tool TMHMM2.0 (Krogh *et al*, 2001).

Generation of expression plasmids

ORF3c genes were PCR amplified using viral cDNA as a template and subsequently inserted into a pCG expression vector co-expressing GFP via an IRES using unique XbaI and MluI restriction sites. To facilitate protein detection, a C-terminal HA-tag (TACCCATACGATGTTCCAGATTACGCT) was added by extension PCR. To generate ORF3c-HA alanine mutants as well as ORF3c-R36I, -BANAL-20-52, −Tor2, and SL-CoVZXC21, point mutations were introduced by site-directed mutagenesis using the wild-type Wuhan-Hu-1 ORF3c-HA expression plasmid as template. pRen2-ORF3c was generated by conventional cloning, using the unique EcoRI and XhoI restriction sites in pRen2. IRF3 5D was PCR amplified using pCAGGS Flag-IRF3 5D as a template and subsequently inserted into pEGFP-C1 via XhoI and EcoRI. All constructs were sequenced to verify their integrity.

Generation and recovery of a recombinant SARS-CoV-2 ORF3 mutant

Stop mutations within ORF3c were introduced into the bacmid pBSCoV2_d6-YFP harboring the SARS-CoV-2 backbone (Herrmann *et al*, 2021) using 2-step Red Recombination (Tischer *et al*, 2006). For this purpose, the KanS cassette was amplified from pEP-KanS with the following oligonucleotides:

1 mut3c-fwd: caattggaactgtaactttgaagcaaggtgaaatcaaggaCgctactccttcagattttgAGGATGACG ACGATAAGTAGGG.
2 mut3c-rev: gtatcgttgcagtagcgcgaacaaaatctgaaggagtagcGtccttgatttcaccttgctCAACCAATTA ACCAATTCTGATTAG.

Integrity of the obtained bacterial artificial chromosomes (BAC) and presence of desired stop mutations were confirmed by restriction digestion and next-generation sequencing. Recombinant SARS-CoV-2 viruses expressing EYFP instead of the viral ORF6 protein and containing mutations within ORF3c were recovered by transfection of the BACs into HEK293T cells overexpressing viral N protein, ACE2 receptor, and T7 RNA polymerase as described previously (Herrmann *et al*, 2021). The obtained reporter viruses were further passaged in CaCo-2 cells and viral titers were determined by endpoint titration (see $TCID_{50}$).

Generation of recombinant SARS-CoV-2 mutants by circular polymerase extension reaction

To generate recombinant SARS-CoV-2 by circular polymerase extension reaction (CPER) (Torii *et al*, 2021), nine DNA fragments comprising parts of SARS-CoV-2 (WK-521, PANGO lineage A; GISAID ID: EPI ISL 408667) (Matsuyama *et al*, 2020) were generated by PCR using PrimeSTAR GXL DNA polymerase (TAKARA, Cat# R050A). A linker fragment comprising hepatitis delta virus ribozyme, the bovine growth hormone poly A signal, and the cytomegalovirus promoter was also prepared by PCR. The 10 obtained DNA fragments were mixed and used for CPER. ORF3c mutations

 © 2023 The Authors

were inserted in fragment 9/10 by site-directed overlap extension PCR.

To produce chimeric recombinant SARS-CoV-2, Tetracycline-inducible ACE2 and TMPRSS-expressing IFNAR1-deficient HEK293 (HEK293-C34) cells were transfected with the CPER products using TransIT-LT1 (MirusBio, Cat#2300) according to the manufacturer's protocol. One day post-transfection, the culture medium was replaced with Dulbecco's modified Eagle's medium (high glucose) containing 2% FCS, 1% PS, and doxycycline. At 7 days post-transfection, the culture medium was harvested and centrifuged, and the supernatants were collected as the seed virus. To remove the CPER products (i.e., any SARS-CoV-2 DNA), 1 ml of the seed virus was treated with 2 μl TURBO DNase (Thermo Fisher Scientific, Cat# AM2238) and incubated at 37°C for 1 h. Complete removal of the CPER products (i.e., SARS-CoV-2-related DNA) from the seed virus was verified by PCR. To prepare virus stocks for infection, VeroE6/TMPRSS2 cells (5,000,000 cells in a T-75 flask) were infected with 20–50 μl of the seed virus. One-hour post-infection, the culture medium was replaced with DMEM (low glucose) containing 2% FBS and 1% PS. Two to four days post-infection, the culture medium was harvested and centrifuged, and the supernatants were collected. Viral titers were determined by $TCID_{50}$. To verify the sequence of chimeric recombinant SARS-CoV-2, viral RNA was extracted from the virus stocks using the QIAamp viral RNA mini kit (Qiagen, Cat#74136) and viral genomes were sequenced as described before (Kimura *et al*, 2022).

Tissue culture infectious dose ($TCID_{50}$)

Viral titers were determined as the 50% tissue culture infectious dose. Briefly, 1 day before infection, VeroE6/TMPRSS2 cells (10,000 cells) were seeded into 96-well plates. Cells were inoculated with serially diluted virus stocks and incubated at 37°C. Four days later, cells were checked microscopically for cytopathic effects (CPE), and $TCID_{50}$/ml was calculated using the Reed–Muench method.

SARS-CoV-2 replication kinetics in CaCo-2, CaLu-3, and *R. alcyone* lung cells

One day before infection with CPER-derived SARS-CoV-2 clones, CaCo-2 cells (10,000 cells/well) or CaLu-3 cells (20,000 cells/well) were seeded into a 96-well plate. Cells were infected with SARS-CoV-2 at an MOI of 0.1 and incubated at 37°C. One hour later, the infected cells were washed and 180 μl of culture medium was added. The culture supernatants and cells were harvested at the indicated time points and used for RT–qPCR to quantify the viral RNA copy number. For replication kinetics of BAC-derived SARS-CoV-2 ΔORF6-YFP and SARS-CoV-2 ΔORF6-YFP ΔORF3c in CaCo-2 cells (10,000 cells/well) or CaLu-3 cells (20,000 cells/well) were seeded into a 96-well plate 1 day prior to infection. Cells were infected in triplicates at an MOI of 0.1 for 1 h at 37°C. After washing and addition of 100 μl fresh culture medium, the plates were placed in an Incucyte plate reader and images were taken at the indicated time points for up to 96 h.

The "Basic Analysis Mode" was applied to quantify virus growth as green area normalized to phase area. Supernatants and cells were harvested at the indicated time points to determine cytokine levels by Cytokine Array and RT–qPCR, respectively. To monitor replication of SARS-CoV-2 ΔORF6-YFP and SARS-CoV-2 ΔORF6-YFP ΔORF3c in *R. alcyone* lung cells, 50,000 cells/well were seeded into a 96-well 1 day prior to infection. Cells were infected at an MOI of 0.1 or 0.001 for 1 h at 37°C. After washing and addition of fresh culture medium, the 96-well plates were placed in an Incucyte plate reader and images were taken at the indicated time points. The "Basic Analysis Mode" was applied to quantify virus growth as green area normalized to phase area.

RT–qPCR

Five microliter culture supernatant was mixed with 5 μl of 2 × RNA lysis buffer [2% Triton X-100, 50 mM KCl, 100 mM Tris–HCl (pH 7.4), 40% glycerol, and 0.8 U/μl recombinant RNase inhibitor] and incubated at room temperature for 10 min. RNase-free water (90 μl) was added, and the diluted sample (2.5 μl) was used as the template for real-time RT–PCR performed according to the manufacturer's protocol using the OneStep TB Green PrimeScript PLUS RT-PCR kit (TAKARA, Cat# RR096A) and the following primers: Forward *N*, 5′-AGC CTC TTC TCG TTC CTC ATC AC-3′; and Reverse *N*, 5′-CCG CCA TTG CCA GCC ATT C-3′. The viral RNA copy number was standardized using a home-made standard.

IFNB1 and *GAPDH* RNA levels were determined in cell lysates collected from (i) SARS-CoV-2 ΔORF6-YFP and SARS-CoV-2 ΔORF6-YFP ΔORF3c-infected CaCo-2 and CaLu wild-type cells and (ii) transfected HEK293T cells infected with Sendai virus (Cantell Strain) (Charles River, 10100774) for 8 h. Total RNA was isolated using the Viral RNA Mini Kit (Qiagen, Cat#74136) according to the manufacturer's instructions. Genomic DNA was removed using the DNA-free kit (Thermo Fischer Scientific, Cat# AM1906) and subsequent cDNA synthesis was performed using the PrimeScript RT reagent Kit (TAKARA, Cat# RR037A), both according to the manufacturer's instructions. qPCR was performed using the Luna Universal Probe qPCR Master Mix (NEB) together with primer probes for IFN-β (Thermo Fischer Scientific, Cat# 4331182) and GAPDH (Thermo Fischer Scientific, Cat# 4448489). All reactions were run in duplicate and RNA levels were internally normalized to GAPDH.

Transfection of HEK293T cells

For overexpression experiments, HEK293T cells were transfected using standard calcium phosphate transfection protocols. 6×10^5 cells were seeded in six-well plates on the day before and medium was changed 6 h after transfection.

Bimolecular fluorescence complementation (BiFC) assay

The BiFC assay was performed essentially as previously described (Sanchez-Aparicio *et al*, 2017). In brief, HEK293T cells were transfected with a combination of expression plasmids encoding YFP amino acids 1–154 (yn) or 155 to the end (yc) conjugated to the N-terminus of RIG-I or MAVS, respectively. Cells were additionally transfected with an expression plasmid encoding different variants of ORF3c-HA or an empty vector control as well as an expression plasmid-encoding BFP as transfection control (overall ratio: 2:2:1:0.5). After 24 h, cells were fixed using the Fix & Perm Kit 1000 (Nordic-MUbio) according to the manufacturer's instructions. ORF3c-HA was detected using a primary antibody against the HA-tag (Sigma-Aldrich, Cat# H3663) followed by staining with a

secondary AF555 antibody (Invitrogen, Cat# A-21422). Samples were measured on a MACS Quant VYB (Miltenyi Biotec). Analysis was performed using FlowLogic V.8 (Inivai) and protein:protein interaction was determined as the MFI of YFP in BFP-positive cells.

Antibodies

The following primary antibodies were used: rabbit polyclonal (IgG) anti-HA (1:1,000, Sigma-Aldrich, Cat# SAB4300603), mouse monoclonal anti-HA (1:1,000, Sigma-Aldrich, Cat# H3663), mouse monoclonal (IgG1) anti-Flag (1:1,000, Sigma-Aldrich, Cat# F1804), rabbit polyclonal (IgG) anti-Flag (1:1,000, Sigma-Aldrich, Cat# SAB4301135), Rabbit anti-IRF3 (1:1,000, Cell Signaling, Cat# 11904S), rabbit anti-pIRF3 (Ser386) (1:1,000, Cell Signaling, Cat# 37829), rat polyclonal (IgG2a, κ) anti-human GAPDH (1:1,000, BioLegend, Cat# 607902), rabbit polyclonal anti-Sendai Virus (1:2,000, MBL Life science, Cat# PD029), rabbit monoclonal (IgG) anti-RIG-I (1:1,000, Cell Signaling, Cat# 3743), rabbit monoclonal (IgG) anti-MDA5 (1:1,000, Cell Signaling, Cat# 5321), rabbit monoclonal (IgG) anti-MAVS (1:1,000, Cell Signaling, Cat# 3993), rabbit monoclonal (IgG) anti-TBK1 (1:1000), Cell Signaling, Cat# 3504).

The following secondary antibodies were used for immunostaining: IRDye® 680RD goat anti-mouse IgG (H + L) (1:20,000, LI-COR, Cat# 926-68070), IRDye® 680RD goat anti-rabbit IgG (H + L) (1:20,000, LI-COR, Cat# 926-68071), IRDye® 800CW goat anti-mouse IgG (H + L) (1:20,000, LI-COR, Cat# 926-32210), IRDye® 800CW goat anti-rabbit IgG (H + L) (1:20,000, LI-COR, Cat# 926-32211), IRDye® 800CW goat anti-streptavidin IgG (H + L) (1:20,000, LI-COR, Cat# 926-32230), Alexa Fluor 488 goat anti-rabbit IgG (1:250, Invitrogen, Cat# A-11008), Alexa Fluor 488 goat anti-mouse IgG (1:250, Invitrogen, Cat# A-11001), Alexa Fluor 555 goat anti-mouse IgG (1:250, Invitrogen, Cat# A-21422), goat anti-rabbit IgG (H + L) cross-adsorbed secondary antibody, and Alexa Fluor 555 (1:250, ThermoFisher, Cat# A-21428).

Co-immunoprecipitation

To investigate possible interactions between ORF3c and proteins of the Interferon signaling pathway, co-immunoprecipitation with subsequent analysis by Western blotting was performed. Briefly, HEK293T cells were seeded in six-well plates and co-transfected with expression plasmids for HA-tagged ORF3c and Flag-tagged RIG-I, MDA5, MAVS or TBK1 (ratio 4:1; 5 μg/well). One day post transfection, cells were lysed in 300 μl Western blot lysis buffer and cleared by centrifugation (see "Western blotting"). Forty-five microliter of the lysate was used for whole-cell lysate analysis and further prepared as described in "Western blotting," while 255 μl of the lysate was used for co-immunoprecipitation. A pre-clearing step was performed to remove unspecifically binding compounds from the lysate. Pierce Protein A/G Magnetic beads (Thermo Fisher, Cat# 88802) were washed three times with 1 ml NP40 wash buffer (50 mM HEPES, 300 mM NaCl, and 0.5% NP40, pH 7.4) and added to the lysate. After incubation for 1 h at 4°C, beads were removed from the lysate using a magnetic rack. To precipitate protein complexes, the lysate was incubated first with an anti-Flag antibody (1.5 μg/sample) for 1 h followed by addition of 15 μl washed Protein A/G Magnetic Beads for one additional hour at 4°C. After incubation, the beads were washed three times in NP40 wash buffer before incubation with 80 μl 1 × Protein Sample Loading Buffer (LI-COR, Cat# 928–40,004) at 95°C for 10 min to recover bound proteins. After addition of 1.75 ml β-mercaptoethanol, whole-cell lysates and precipitates were analyzed by Western blotting.

Western blotting

To determine expression of cellular and viral proteins, cells were washed in PBS, lysed in Western blot lysis buffer (150 mM NaCl, 50 mM HEPES, 5 mM EDTA, 0.1% NP40, 500 mM Na_3VO_4, and 500 mM NaF, pH 7.5) containing protease and phosphatase inhibitors and cleared by centrifugation at 20,800 *g* for 20 min at 4°C. Lysates were mixed with Protein Sample Loading Buffer (LI-COR, Cat# 928-40004) supplemented with 10% β-mercaptoethanol and heated at 95°C for 5 min. Proteins were separated on NuPAGE 4%–12% Bis–Tris Gels (Thermo Fischer Scientific, Cat# NP0323BOX), blotted onto Immobilon-FL PVDF membranes (Merck Milipore, Cat# IPFL00010) and stained using primary antibodies directed against HA-tag, Flag-tag, GAPDH, RIG-I, MDA5, MAVS, TBK1, IRF3, and infrared dye-labeled secondary antibodies (LI-COR IRDye). All washing and blocking steps were performed using PBS containing 0.2% Tween 20 and 1–5% Milk. To detect phosphorylated proteins, membranes were washed in TBS containing 0.2% Tween 20 and blocked in PBS containing 0.2% Tween 20 and 5% Milk. Proteins were detected using a LI-COR Odyssey scanner and band intensities were quantified using LI-COR Image Studio Lite Version 5.2.

Cytokine Array

To detect cytokines in the supernatants of virus-infected cells, the Human Cytokine Array C16 kit (RayBiotech) was used with adjustments to the manufacturer's protocol. Specifically, instead of the provided secondary HRP-streptavidin antibody, a streptavidin-human IRDye 800CW (LI-COR) secondary antibody in PBST was used to incubate the membranes for 30 min at room temperature. Cytokines were detected using an infrared LI-COR Odyssey Imager as described in Western blotting.

Immunofluorescence microscopy

Confocal immunofluorescence microscopy was used to determine the subcellular localization of ORF3c and ORF3c R36I, as well as co-localization of ORF3c with mitochondria and nuclear translocation of endogenous IRF3. Briefly, 150,000 HEK293T cells were seeded on 13-mm-diameter glass coverslips coated with poly-L-lysine (Sigma-Aldrich, Cat# P6282-5 mg) in 24-well plates. On the following day, cells were transfected (500 ng) using Lipofectamine 2000 (Invitrogen, Cat# 11668019) according to the manufacturer's instructions. One day post-transfection, cells were fixed in 4% PFA for 20 min at RT, permeabilized in PBS 0.5% Triton X-100 for 15 min at RT, and blocked in 5% BSA/PBS supplemented with 0.1% Triton X-100 for 15 min at RT. ORF3c was stained using a primary antibody against the HA-tag (Sigma-Aldrich, Cat# H3663) and secondary goat anti-mouse AF488 (Invitrogen, Cat# A-11001). IRF3 was detected using an anti-IRF3 antibody (Cell Signaling, Cat# 11904S) followed by secondary staining with goat anti-rabbit AF555 (ThermoFisher, Cat# A-21428). Mitochondria were labeled by transfection with DsRed2-Mito7 (Addgene; Cat# 55838). Nuclei were stained in parallel using

 © 2023 The Authors

4′,6-Diamidine-2′-phenylindole (DAPI; Thermo Fischer Scientific, Cat# 62247). Coverslips were mounted on glass slides using Mowiol (CarlRoth, Cat# 0713.1) mounting medium, and confocal microscopy was performed using an LSM710 (Carl Zeiss). No blinding was done during image acquisition.

Flow cytometry

HEK293T cells were transfected with increasing amounts of a reporter plasmid for ORF3c-HA to determine the effect on endogenous IRF3 levels. Transfection with expression plasmids for NSP1 from the rotaviruses NCDV (reduces IRF3 expression) or OSU (inactive against IRF3) (both co-expressing YFP) served as controls (Sen *et al*, 2009). One day post-transfection, cells were washed in PBS with 2% FCS, fixed in 4% PFA, and fluorescence was determined using a MACS Quant VYB (Miltenyi Biotec). To quantify IRF3 protein levels, cells were permeabilized using the FIX & PERM kit (Nordic-MUbio, #GAS-002-1) according to the manufacturer's instructions. Subsequently, cells were stained using anti-IRF3 (Cell Signaling, Cat# 11904S) followed by secondary staining with Alexa Fluor 555 (ThermoFisher, Cat# A-21428).

Firefly luciferase assay

HEK293T cells were seeded in 96-well plates at 3×10^4 cells/well. After 24 h, cells were transfected with a mix of expression vectors containing firefly luciferase reporter constructs for the IFN-β promoter or a mutant thereof lacking NF-κB-biding sites (IFN-β Δ-NF-κB) (reporter, 10 ng), a *Gaussia* luciferase expression plasmid (normalization control, 5 ng), expression plasmids for RIG-I-CARD, MDA5, MAVS, or IRF3 5D (stimulus, 5 ng), different amounts of ORF3 expression constructs (12.5–100 ng), and empty vector (pCG_HIV-1 M NL4-3 *nef* stop ΔIRES-eGFP) to adjust total DNA amounts across all conditions to 200 ng/well. After 24 h, supernatants were harvested, and cells were lysed in 100 μl 1× Passive Lysis Buffer (Promega, Cat# E194A). *Gaussia* luciferase activity in the supernatants was measured by addition of Coelenterazine (PJK Biotech, Cat# 102174). Firefly luciferase activity was measured in the cells using the Luciferase Assay System (Promega, Cat# E1501) according to the manufacturer's instruction.

LIPS assay

Luciferase fusion protein (10^6 RLU) in 50 μl Buffer A (50 mM Tris, 150 mM NaCl, and 0.1% Triton X-100, pH 7.5) and 1 μl sample serum (CoV-PosSet-S2-RB; COVID-19 Serum Sample Set 2; BioCat) in 49 μl Buffer A were added to 1.5 ml tubes and incubated with shaking at 300 rpm for 1 h at room temperature. Pierce Protein A/G Magnetic beads were added to each condition as a 30% suspension in PBS for an additional hour and shaking at room temperature. Samples were placed on a magnetic rack, and supernatant was removed after 1-min incubation. Magnetic beads were washed twice with 150 μl Buffer A followed by two washes with 150 μl PBS. Samples were transferred into a 96-well opaque Nunc plate (VWR), and 50 μl Coelenterazine (PJK Biotech, Cat# 102174) was added to each condition. Samples were measured immediately on a TriStar² S LB 942 Multimode Reader (Berthold Technologies) with an integration time of 0.1 s and a read height of 1 mm.

Quantification and statistical analysis

Statistical analyses were performed using GraphPad PRISM 9.4.1. For statistical testing between two means, *P*-values were calculated using paired or unpaired Student's *t*-test. For comparison within one group, we used one-way analysis of variation (ANOVA) with Dunnett's multiple-comparison test, and for comparison between two or more groups we used two-way ANOVA with Sidak's multiple comparison test. Unless otherwise stated, data are shown as the mean of at least three biological replicates ± s.d. Statistical parameters are specified in the figure legends.

Data availability

This study includes no data deposited in external repositories.

Expanded View for this article is available online.

Acknowledgements

We thank Isabell Haußmann and Veronique Baudy for excellent technical assistance and Zinaida Klestova for performing literature search. We would like to thank the following persons for providing helpful reagents: Peter D Burbelo (LIPS assay plasmids), Konstantin MJ Sparrer (RIG-I, MDA5, and TBK1 expression plasmids, and IRF luciferase reporter plasmid), Harmit Malik (MAVS expression plasmid), Rongtuan Lin (IRF3 expression plasmid), Bernd Baumann (NF-κB luciferase reporter plasmid, *Gaussia* luciferase control vector, and IKKβ expression plasmid), Michael Gale Jr (IFN-β promoter reporter plasmid), Prof. Takasuke Fukuhara (CPER plasmids and cell lines), and Prof. Takashi Irie (pCAGGS Flag-IRF3 5d). SARS-CoV-2 mNeon Green was kindly provided by Prof. Michael Schindler. We would also like to thank Prof. Adolfo García-Sastre and the CEIRR program (NIAD Centers of Excellence for Influenza Research and Response) for providing BiFC reporter plasmids. DsRed2-Mito-7 was a gift from Michael Davidson. NSP1 expression constructs were kindly provided by Michele Hardy. We gratefully acknowledge all data contributors, that is, the authors and their originating laboratories responsible for obtaining the specimens, and their submitting laboratories for generating the genetic sequence and metadata and sharing via the GISAID Initiative, on which parts of this research are based. Graphical abstract and synopsis were created with BioRender.com. This work was funded by the Federal Ministry of Education and Research Germany (BMBF; grant ID: FKZ 01KI20135), the Canon Foundation Europe, the Heisenberg Program of the German Research Foundation (DFG; grant ID: SA 2676/3-1), and grants of the COVID-19 program of the Ministry of Science, Research and the Arts Baden-Württemberg (MWK; grants IDs: MWK K.N.K.C.014 (U21) and MWK K.N.K.C.015 (U22)) to DS. AE and AH were supported by BMBF SenseCoV2 01KI20172A; DFG Fokus COVID-19, EN 423/7-1; and Coronavirus research grants by the Bavarian State Ministry of Science and the Arts and Bavarian State Ministry of Health, Bay-VOC. Open Access funding enabled and organized by Projekt DEAL.

Author contributions

Martin Müller: Conceptualization; data curation; formal analysis; supervision; validation; investigation; visualization; methodology; writing – review and editing. **Alexandra Herrmann:** Resources; methodology. **Shigeru Fujita:** Formal analysis; validation; investigation; methodology. **Keiya Uriu:** Formal analysis; validation; investigation. **Carolin Kruth:** Formal analysis; validation; investigation; writing – review and editing. **Adam Strange:** Formal analysis; methodology. **Jan E Kolberg:** Validation; investigation. **Markus Schneider:**

© 2023 The Authors

Resources; validation; methodology. **Jumpei Ito:** Formal analysis; methodology. **Marcel A Müller:** Resources; writing – review and editing. **Christian Drosten:** Resources. **Armin Ensser:** Resources; supervision; funding acquisition. **Kei Sato:** Resources; supervision; funding acquisition; methodology. **Daniel Sauter:** Conceptualization; resources; data curation; formal analysis; supervision; funding acquisition; validation; investigation; visualization; methodology; writing – original draft; project administration; writing – review and editing.

Disclosure and competing interests statement

The authors declare that they have no conflict of interest.

References

Anggakusuma, Brown RJP, Banda DH, Todt D, Vieyres G, Steinmann E, Pietschmann T (2016) Hepacivirus NS3/4A proteases interfere with MAVS signaling in both their cognate animal hosts and humans: implications for zoonotic transmission. *J Virol* 90: 10670–10681

Bykova A, Saura A, Glazko GV, Roche-Lima A, Yurchenko V, Rogozin IB (2023) The 29-nucleotide deletion in SARS-CoV: truncated versions of ORF8 are under purifying selection. *BMC Genomics* 24: 387

Cagliani R, Forni D, Clerici M, Sironi M (2020) Coding potential and sequence conservation of SARS-CoV-2 and related animal viruses. *Infect Genet Evol* 83: 104353

Camproux AC, Gautier R, Tuffery P (2004) A hidden markov model derived structural alphabet for proteins. *J Mol Biol* 339: 591–605

Finkel Y, Mizrahi O, Nachshon A, Weingarten-Gabbay S, Morgenstern D, Yahalom-Ronen Y, Tamir H, Achdout H, Stein D, Israeli O *et al* (2021) The coding capacity of SARS-CoV-2. *Nature* 589: 125–130

Firth AE (2020) A putative new SARS-CoV protein, 3c, encoded in an ORF overlapping ORF3a. *J Gen Virol* 101: 1085–1089

Firth AE, Brierley I (2012) Non-canonical translation in RNA viruses. *J Gen Virol* 93: 1385–1409

Gleason AC, Ghadge G, Chen J, Sonobe Y, Roos RP (2022) Machine learning predicts translation initiation sites in neurologic diseases with nucleotide repeat expansions. *PLoS One* 17: e0256411

Gong P, Shen Q, Zhang M, Qiao R, Jiang J, Su L, Zhao S, Fu S, Ma Y, Ge L *et al* (2023) Plant and animal positive-sense single-stranded RNA viruses encode small proteins important for viral infection in their negative-sense strand. *Mol Plant* https://doi.org/10.1016/j.molp.2023.09.020

Hachim A, Gu H, Kavian O, Mori M, Kwan MYW, Chan WH, Yau YS, Chiu SS, Tsang OTY, Hui DSC *et al* (2022) SARS-CoV-2 accessory proteins reveal distinct serological signatures in children. *Nat Commun* 13: 2951

Hayn M, Hirschenberger M, Koepke L, Nchioua R, Straub JH, Klute S, Hunszinger V, Zech F, Prelli Bozzo C, Aftab W *et al* (2021) Systematic functional analysis of SARS-CoV-2 proteins uncovers viral innate immune antagonists and remaining vulnerabilities. *Cell Rep* 35: 109126

Herrmann A, Jungnickl D, Cordsmeier A, Peter AS, Uberla K, Ensser A (2021) Cloning of a passage-free SARS-CoV-2 genome and mutagenesis using red recombination. *Int J Mol Sci* 22: 10188

Horner SM, Wilkins C, Badil S, Iskarpatyoti J, Gale M Jr (2015) Proteomic analysis of mitochondrial-associated ER membranes (MAM) during RNA virus infection reveals dynamic changes in protein and organelle trafficking. *PLoS One* 10: e0117963

Jungreis I, Nelson CW, Ardern Z, Finkel Y, Krogan NJ, Sato K, Ziebuhr J, Stern-Ginossar N, Pavesi A, Firth AE *et al* (2021a) Conflicting and ambiguous names of overlapping ORFs in the SARS-CoV-2 genome: a homology-based resolution. *Virology* 558: 145–151

Jungreis I, Sealfon R, Kellis M (2021b) SARS-CoV-2 gene content and COVID-19 mutation impact by comparing 44 Sarbecovirus genomes. *Nat Commun* 12: 2642

Khare S, Gurry C, Freitas L, Schultz MB, Bach G, Diallo A, Akite N, Ho J, Lee RT, Yeo W *et al* (2021) GISAID's role in pandemic response. *China CDC Wkly* 3: 1049–1051

Kimura I, Konno Y, Uriu K, Hopfensperger K, Sauter D, Nakagawa S, Sato K (2021) Sarbecovirus ORF6 proteins hamper induction of interferon signaling. *Cell Rep* 34: 108916

Kimura I, Yamasoba D, Nasser H, Zahradnik J, Kosugi Y, Wu J, Nagata K, Uriu K, Tanaka YL, Ito J *et al* (2022) The SARS-CoV-2 spike S375F mutation characterizes the Omicron BA.1 variant. *iScience* 25: 105720

Konno Y, Kimura I, Uriu K, Fukushi M, Irie T, Koyanagi Y, Sauter D, Gifford RJ, USFQ-COVID19 Consortium, Nakagawa S *et al* (2020) SARS-CoV-2 ORF3b is a potent interferon antagonist whose activity is increased by a naturally occurring elongation variant. *Cell Rep* 32: 108185

Kopecky-Bromberg SA, Martinez-Sobrido L, Frieman M, Baric RA, Palese P (2007) Severe acute respiratory syndrome coronavirus open reading frame (ORF) 3b, ORF 6, and nucleocapsid proteins function as interferon antagonists. *J Virol* 81: 548–557

Krogh A, Larsson B, von Heijne G, Sonnhammer EL (2001) Predicting transmembrane protein topology with a hidden Markov model: application to complete genomes. *J Mol Biol* 305: 567–580

Li JY, Liao CH, Wang Q, Tan YJ, Luo R, Qiu Y, Ge XY (2020) The ORF6, ORF8 and nucleocapsid proteins of SARS-CoV-2 inhibit type I interferon signaling pathway. *Virus Res* 286: 198074

Li X, Hou P, Ma W, Wang X, Wang H, Yu Z, Chang H, Wang T, Jin S, Wang X *et al* (2022) SARS-CoV-2 ORF10 suppresses the antiviral innate immune response by degrading MAVS through mitophagy. *Cell Mol Immunol* 19: 67–78

Matsuyama S, Nao N, Shirato K, Kawase M, Saito S, Takayama I, Nagata N, Sekizuka T, Katoh H, Kato F *et al* (2020) Enhanced isolation of SARS-CoV-2 by TMPRSS2-expressing cells. *Proc Natl Acad Sci U S A* 117: 7001–7003

Min YQ, Huang M, Sun X, Deng F, Wang H, Ning YJ (2021) Immune evasion of SARS-CoV-2 from interferon antiviral system. *Comput Struct Biotechnol J* 19: 4217–4225

Miorin L, Kehrer T, Sanchez-Aparicio MT, Zhang K, Cohen P, Patel RS, Cupic A, Makio T, Mei M, Moreno E *et al* (2020) SARS-CoV-2 Orf6 hijacks Nup98 to block STAT nuclear import and antagonize interferon signaling. *Proc Natl Acad Sci U S A* 117: 28344–28354

Mozzi A, Oldani M, Forcella ME, Vantaggiato C, Cappelletti G, Pontremoli C, Valenti F, Forni D, Saresella M, Biasin M *et al* (2023) SARS-CoV-2 ORF3c impairs mitochondrial respiratory metabolism, oxidative stress, and autophagic flux. *iScience* 26: 107118

Muth D, Corman VM, Roth H, Binger T, Dijkman R, Gottula LT, Gloza-Rausch F, Balboni A, Battilani M, Rihtaric D *et al* (2018) Attenuation of replication by a 29 nucleotide deletion in SARS-coronavirus acquired during the early stages of human-to-human transmission. *Sci Rep* 8: 15177

Nelson CW, Ardern Z, Goldberg TL, Meng C, Kuo CH, Ludwig C, Kolokotronis SO, Wei X (2020) Dynamically evolving novel overlapping gene as a factor in the SARS-CoV-2 pandemic. *Elife* 9: e59633

Oulas A, Zanti M, Tomazou M, Zachariou M, Minadakis G, Bourdakou MM, Pavlidis P, Spyrou GM (2021) Generalized linear models provide a measure of virulence for specific mutations in SARS-CoV-2 strains. *PLoS One* 16: e0238665

Pavesi A (2020) New insights into the evolutionary features of viral overlapping genes by discriminant analysis. *Virology* 546: 51–66

 © 2023 The Authors

Sanchez-Aparicio MT, Ayllon J, Leo-Macias A, Wolff T, Garcia-Sastre A (2017) Subcellular localizations of RIG-I, TRIM25, and MAVS complexes. *J Virol* 91: e01155-16

Sen A, Feng N, Ettayebi K, Hardy ME, Greenberg HB (2009) IRF3 inhibition by rotavirus NSP1 is host cell and virus strain dependent but independent of NSP1 proteasomal degradation. *J Virol* 83: 10322 – 10335

Seth RB, Sun L, Ea CK, Chen ZJ (2005) Identification and characterization of MAVS, a mitochondrial antiviral signaling protein that activates NF-kappaB and IRF 3. *Cell* 122: 669 – 682

Strahle L, Garcin D, Kolakofsky D (2006) Sendai virus defective-interfering genomes and the activation of interferon-beta. *Virology* 351: 101 – 111

Tischer BK, von Einem J, Kaufer B, Osterrieder N (2006) Two-step red-mediated recombination for versatile high-efficiency markerless DNA manipulation in *Escherichia coli*. *Biotechniques* 40: 191 – 197

Torii S, Ono C, Suzuki R, Morioka Y, Anzai I, Fauzyah Y, Maeda Y, Kamitani W, Fukuhara T, Matsuura Y (2021) Establishment of a reverse genetics system for SARS-CoV-2 using circular polymerase extension reaction. *Cell Rep* 35: 109014

Varga ZT, Ramos I, Hai R, Schmolke M, Garcia-Sastre A, Fernandez-Sesma A, Palese P (2011) The influenza virus protein PB1-F2 inhibits the induction of type I interferon at the level of the MAVS adaptor protein. *PLoS Pathog* 7: e1002067

Varga ZT, Grant A, Manicassamy B, Palese P (2012) Influenza virus protein PB1-F2 inhibits the induction of type I interferon by binding to MAVS and decreasing mitochondrial membrane potential. *J Virol* 86: 8359 – 8366

Weingarten-Gabbay S, Chen DY, Sarkizova S, Taylor HB, Gentili M, Pearlman LR, Bauer MR, Rice CM, Clauser KR, Hacohen N *et al* (2023) The HLA-II immunopeptidome of SARS-CoV-2. *bioRxiv* https://doi.org/10.1101/2023.05.26.542482 [PREPRINT]

Xia H, Cao Z, Xie X, Zhang X, Chen JY, Wang H, Menachery VD, Rajsbaum R, Shi PY (2020) Evasion of type I interferon by SARS-CoV-2. *Cell Rep* 33: 108234

License: This is an open access article under the terms of the Creative Commons Attribution License, which permits use, distribution and reproduction in any medium, provided the original work is properly cited.

Expanded View Figures

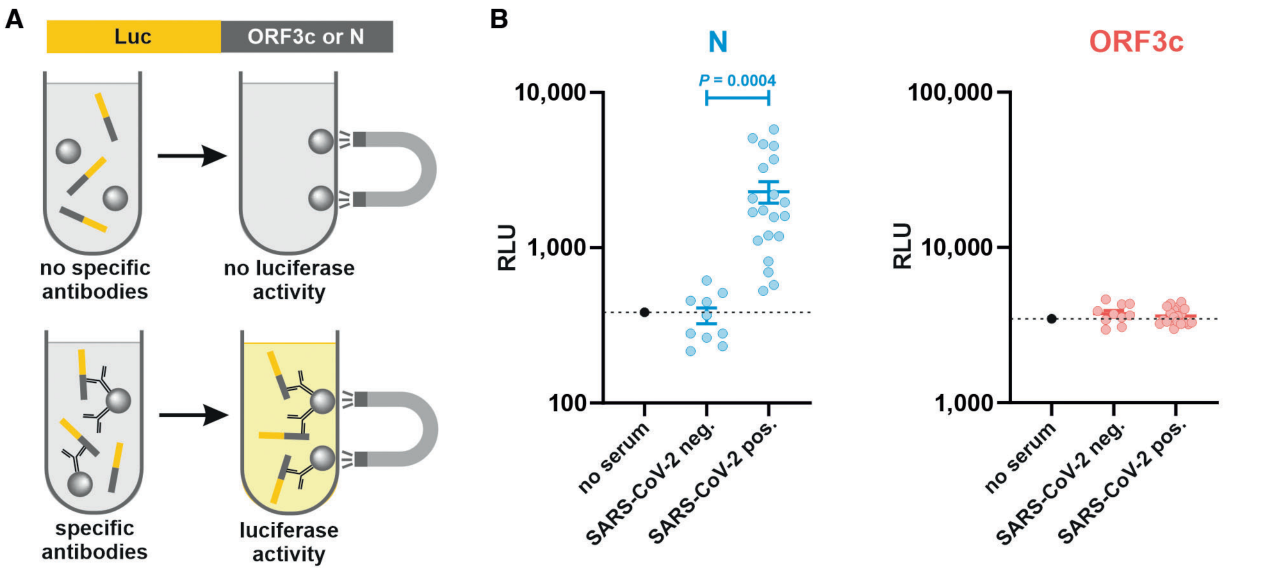

Figure EV1. Detection of SARS-CoV-2-specific antibodies via Luciferase Immunoprecipitation System (LIPS).

A Principle of the LIPS assay: HEK293T cells are transfected with expression plasmids for a viral protein of interest fused to Renilla luciferase. Subsequently, transfected cells are lysed and incubated with serum samples and magnetic beads. Antibodies against viral proteins of interest will cross-link the luciferase-containing proteins with beads and allow magnet-assisted pull-down of both beads and luciferase activity.

B LIPS-mediated quantification of antibodies against SARS-CoV-2 N (left panel, n = 10) and ORF3c (right panel, n = 20) in sera from SARS-CoV-2 naïve and convalescent sera (RLU, relative light units). Each dot represents one independent serum sample. Data are shown as mean ± s.e.m. Differences in antibody levels between SARS-CoV-2 naïve and convalescent sera were determined by unpaired, one-tailed student's t-test.

Source data are available online for this figure.

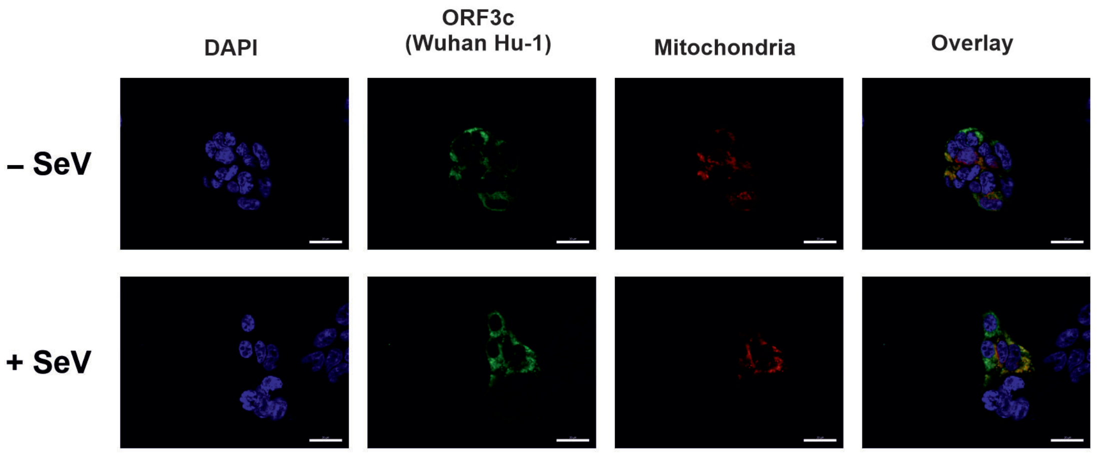

Figure EV2. ORF3c co-localizes with mitochondria.

Representative images of HEK293T cells transfected with the expression plasmid dsRedMito coding for a mitochondria marker, as well as expression plasmids for Wuhan Hu-1 ORF3c or an empty vector control. One day post-transfection, cells were infected with SeV for 6 h and subsequently stained for ORF3c (anti-HA, green) and nuclei (DAPI, blue) (scale bar = 20 µm).

Source data are available online for this figure.

 © 2023 The Authors

Figure EV3. ORF3c affects IRF3 activation and translocation but not total protein levels.

A HEK293T cells were transfected with either Wuhan Hu-1 ORF3c or an empty vector control and stimulated with SeV 6 h p.t. Cells were harvested at the indicated time points, and (phosphorylated) IRF3 was analyzed by western blotting. Data are representative of three biological replicates ($n = 3$).

B Representative images of HEK293T cells transfected with expression plasmids for Wuhan Hu-1 ORF3c or an empty vector control. One day post-transfection, cells were treated with SeV for 6 h and subsequently stained for ORF3c (anti-HA, green), IRF3 (endogenous, red) and nuclei (DAPI, blue) (scale bar = 20 μm). Weighted colocalization coefficient of IRF3 with DAPI was determined as a marker for IRF3 translocation into the nucleus (n = 28–32 cells in each condition analyzed). Data are shown as individual cells with the median and quartiles indicated as dotted lines (right panels).

C HEK293T cells were transfected with increasing amounts of expression plasmids for Wuhan Hu-1 ORF3c and IRES eGFP. Transfection with expression plasmids for NSP1 from the rotaviruses NCDV (reduces IRF3 expression) or OSU (inactive against IRF3) (both co-expressing YFP) served as controls. One day post-transfection cells were fixed, permeabilized and stained against IRF3 (endogenous, AF555) before FACS analysis. Numbers in gates indicate MFI of IRF3-AF555 (left). Levels of IRF3 expression are shown as MFI of AF555 in GFP-positive over GFP-negative cells. Data are shown as bars (min to max) with median of three biological replicates ($n = 3$) and were analyzed by one-way ANOVA with Dunnett's multiple-comparison test.

Source data are available online for this figure.

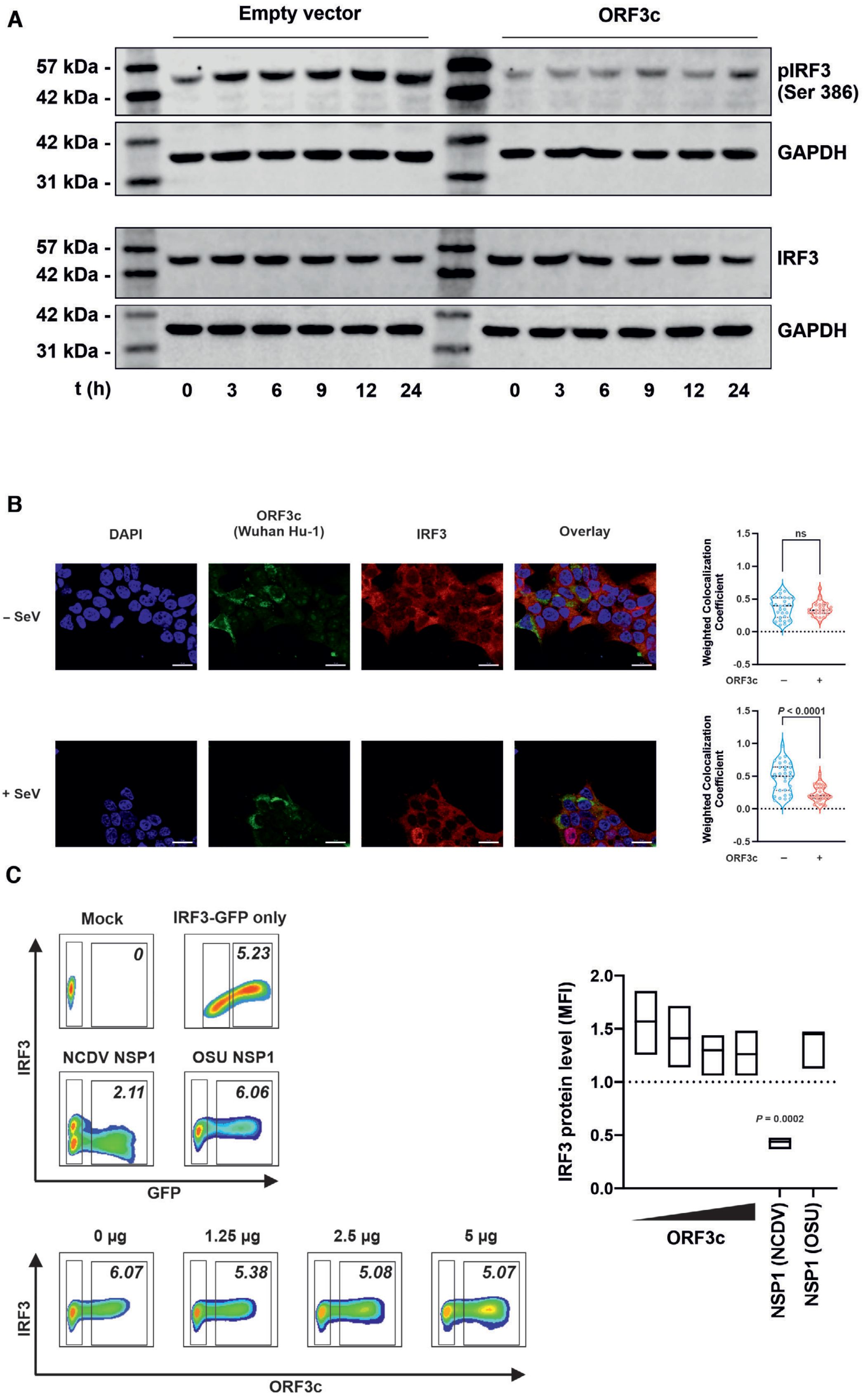

Figure EV3.

 © 2023 The Authors

Figure EV4. Targeting of MAVS by different ORF3c orthologs and mutants.

A HEK293T cells were co-transfected with expression plasmids for Flag-tagged MAVS and expression plasmids for the indicated HA-tagged SARS-CoV-2 ORF3c variants. One day post-transfection, cells were lysed. Cell lysates were analyzed by Western blotting, either directly ("input") or upon pull-down using a Flag-specific antibody ("IP"). Data are representative of two biological replicates (n = 2). The red arrow indicates a faint ORF3c band.

B HEK293T cells were co-transfected with expression plasmids for blue fluorescent protein (BFP) and expression plasmids for the indicated HA-tagged SARS-CoV-2 ORF3c variants. One day post-transfection, cells were permeabilized, stained for the HA-tag, and analyzed by flow cytometry. BFP-positive (i.e. transfected) cells were gated, and the percentage of HA-ORF3c-positive (i.e. PE-positive) cells was determined. Exemplary primary data of one out of three biological replicates are shown.

C HEK293T cells were transfected with plasmids expressing BFP, the indicated ORF3c orthologs, the C-terminal part of YFP fused to RIG-I and/or the N-terminal part of YFP fused to MAVS. Twenty-four hours later, cells were fixed, and YFP fluorescence was detected by flow cytometry as a reporter for MAVS-RIG-I interaction. Data are shown as mean and s.d. of three biological replicates (n = 3) and were analyzed by one-way ANOVA with Dunnett's multiple-comparison test.

Source data are available online for this figure.

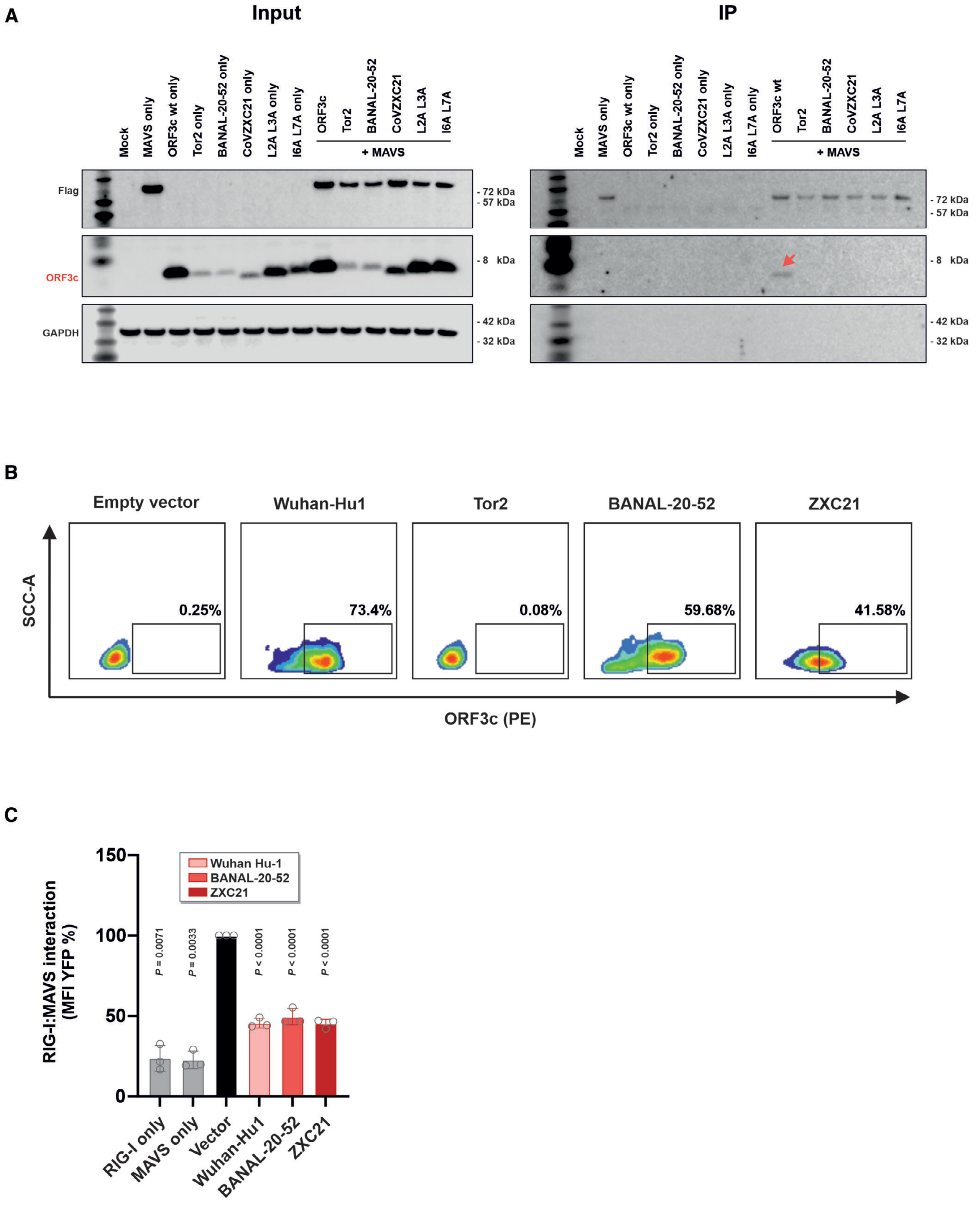

Figure EV4.

 © 2023 The Authors

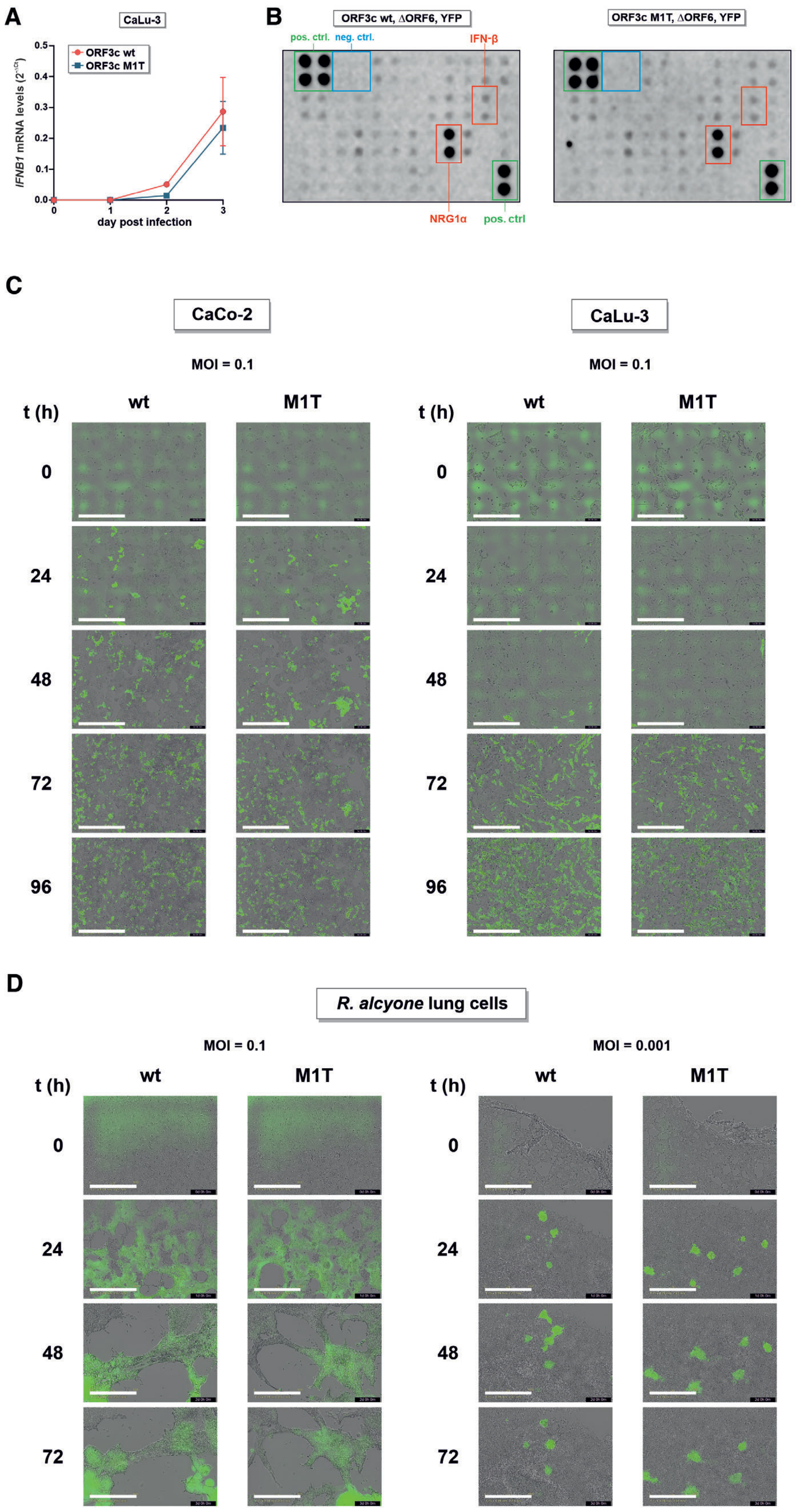

Figure EV5. Cytokine expression and release from cells infected with wild-type or ORF3c-deficient SARS-CoV-2.

A *IFNB1* mRNA levels of the infected CaLu-3 cells in Fig 5A were determined by qPCR. Mean values of four biological replicates ± s.e.m. are shown. *IFNB1* mRNA levels were not or hardly detectable in the respective experiment in CaCo-2 cells (see source data).

B Ninety-six hours post-infection, supernatants of one of the CaLu-3 experiment shown in Fig 5C were harvested and cytokine amounts were quantified using a membrane-based array. This approach enables a semi-quantitative analysis of cytokine release. The signal intensity of each dot indicates the amount of a specific cytokine in the culture supernatant (in duplicates). Positive and negative controls are highlighted in green and blue, respectively. Selected cytokines are highlighted in red. Data are representative of two biological replicates ($n = 2$).

C CaCo-2 and CaLu-3 cells were infected with SARS-CoV-2 ΔORF6-YFP or SARS-CoV-2 ΔORF6-YFP ΔORF3c and analyzed as described in Fig 5C. The images show virus-infected cells (green) over the indicated time points post-infection from one randomly chosen well of one of the replicates of Fig 5C (scale bar = 600 μm).

D *R. alcyone* lung cells stably expressing human ACE2 were infected with SARS-CoV-2 ΔORF6-YFP or SARS-CoV-2 ΔORF6-YFP ΔORF3c and analyzed as described in Fig 5D. The images show virus-infected cells (green) over the indicated time points post-infection from one randomly chosen well of one of the replicates of Fig 5D (scale bar = 600 μm).

Source data are available online for this figure.
