# Supplementary material for: ORF3c is expressed in SARS‐CoV‐2‐infected cells and inhibits innate sensing by targeting MAVS
Source: EMBO Rep. 2023 Oct 23;24(12):e57137. doi: 10.15252/embr.202357137 (PMC10702836; doi:10.15252/embr.202357137)
Supplement: Supplementary file 4 — Source Data for Figure 1 [file EMBR-24-e57137-s003.zip › Fig1C/EMBOR-2023-57137V2_SourceDataForFigure1C.pptx]

## Slide 1
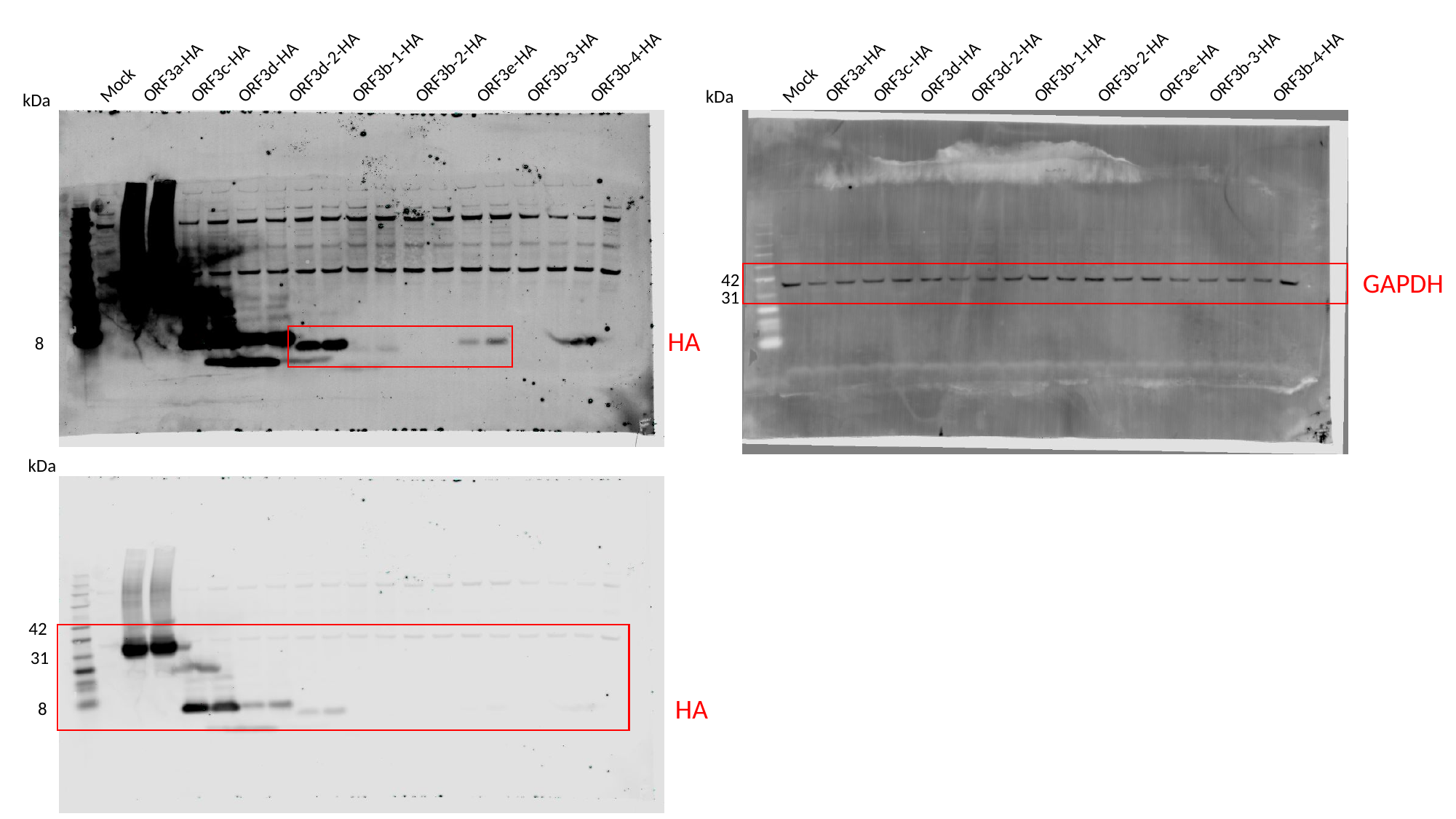

ORF3d-2-HA
ORF3b-1-HA
ORF3b-2-HA
ORF3b-3-HA
ORF3b-4-HA
ORF3d-2-HA
ORF3b-1-HA
ORF3b-2-HA
ORF3b-3-HA
ORF3b-4-HA
ORF3d-HA
ORF3d-HA
ORF3e-HA
ORF3e-HA
ORF3a-HA
ORF3a-HA
ORF3c-HA
ORF3c-HA
Mock
Mock
kDa
kDa
GAPDH
42
31
HA
8
kDa
42
31
HA
8
