# Supplementary figures and images for: ORF3c is expressed in SARS‐CoV‐2‐infected cells and inhibits innate sensing by targeting MAVS
Source: EMBO Rep. 2023 Oct 23;24(12):e57137. doi: 10.15252/embr.202357137 (PMC10702836; doi:10.15252/embr.202357137)

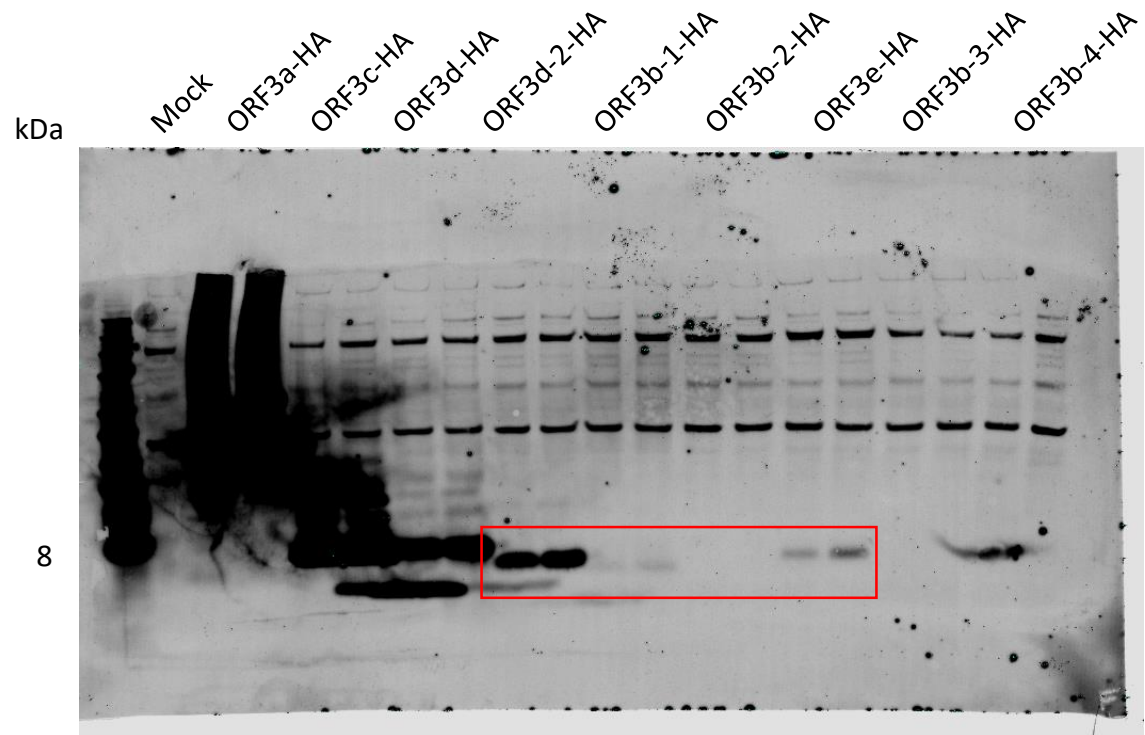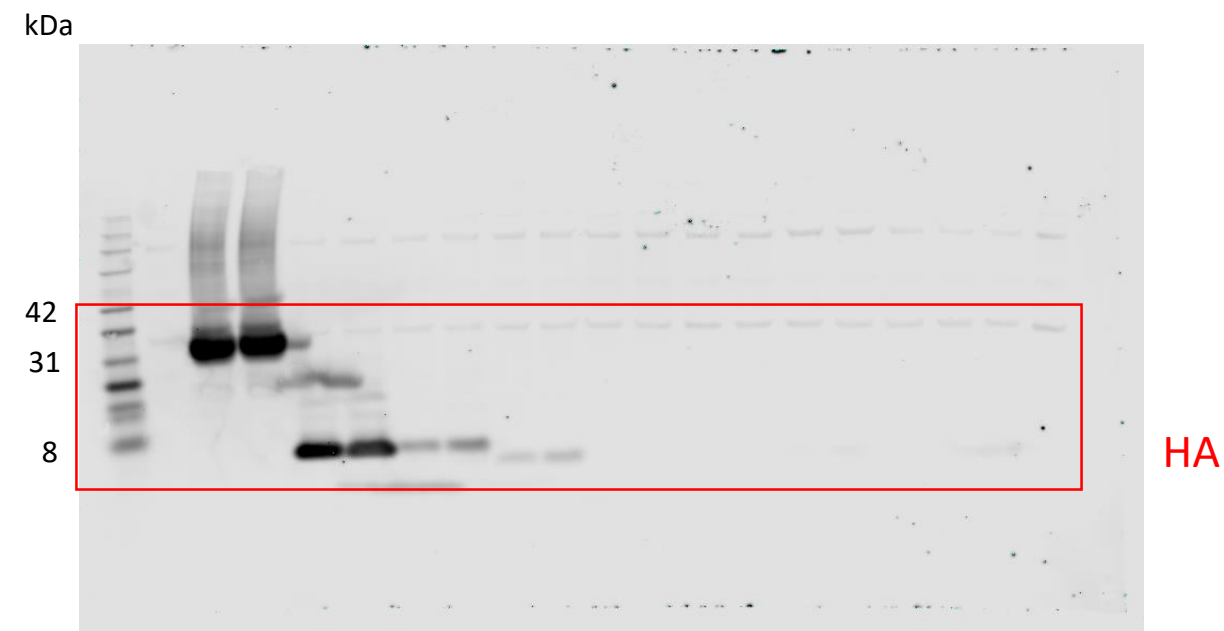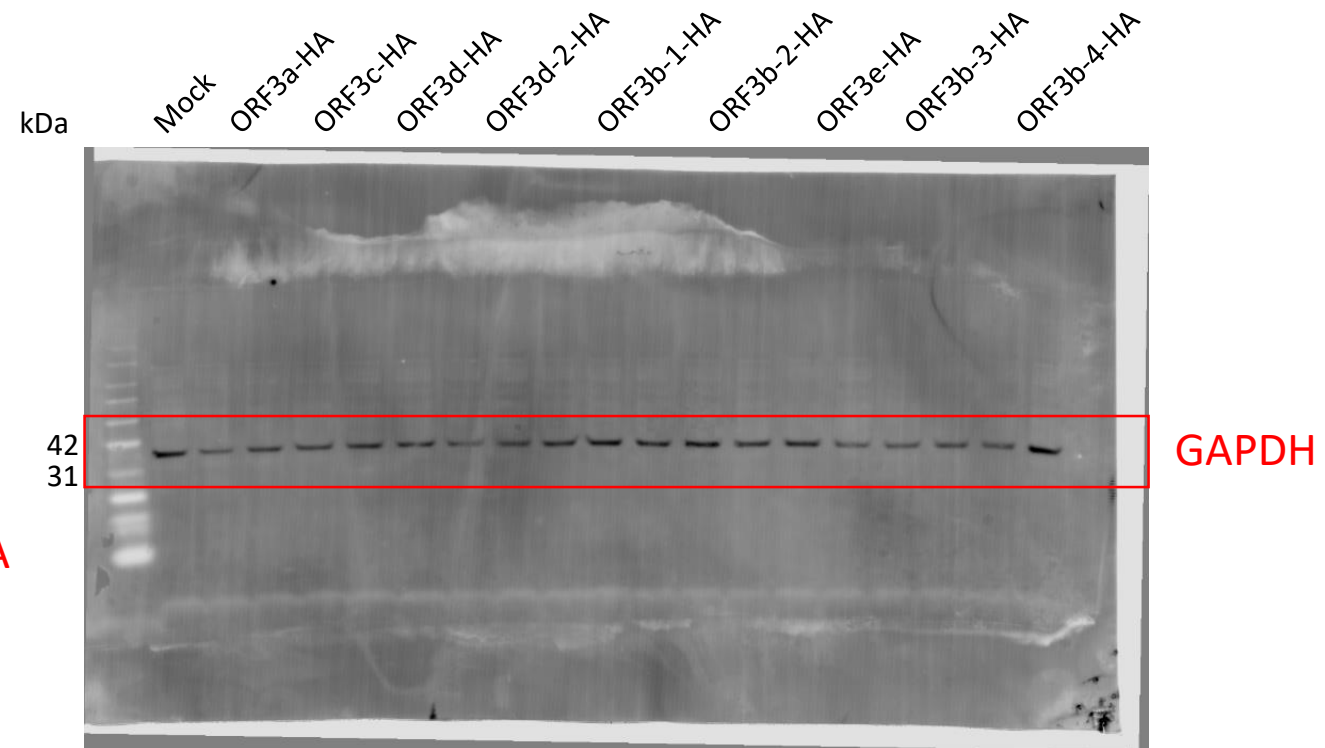

Supplement: Supplementary file 4 — Source Data for Figure 1 [file EMBR-24-e57137-s003.zip › Fig1C/EMBOR-2023-57137V2_SourceDataForFigure1C.pdf]

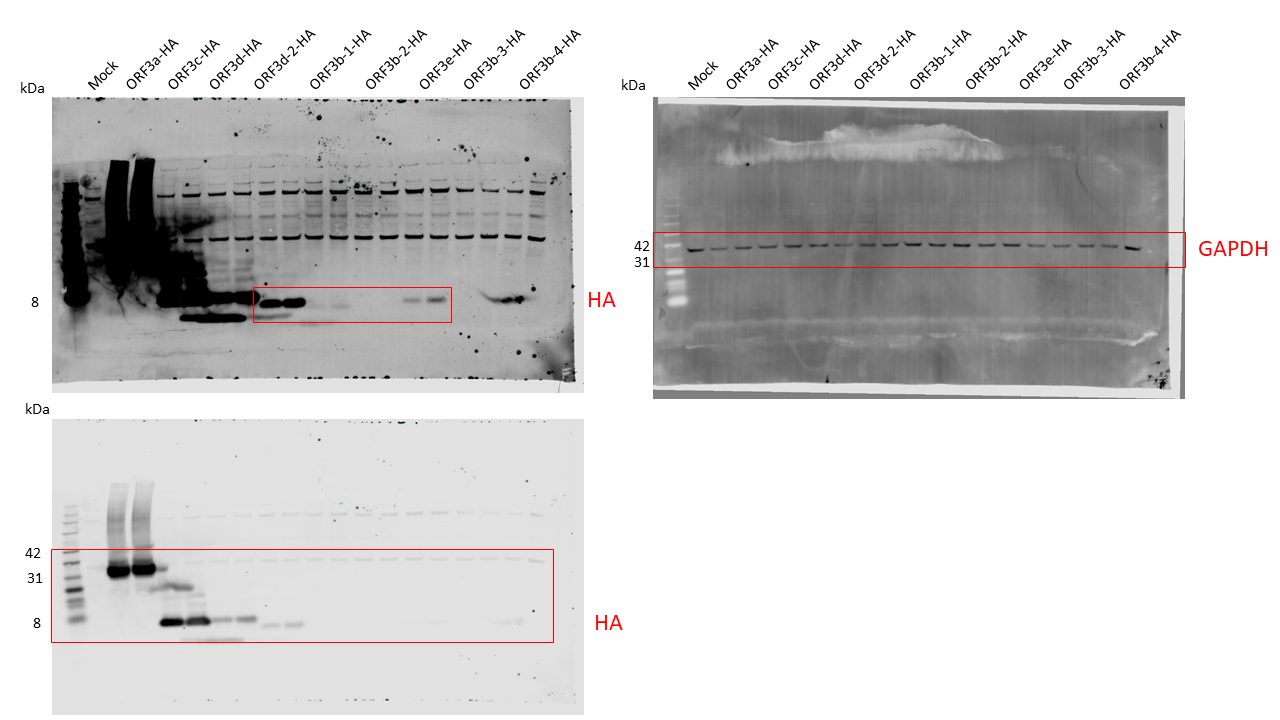

Supplement: Supplementary file 4 — Source Data for Figure 1 [file EMBR-24-e57137-s003.zip › Fig1C/EMBOR-2023-57137V2_SourceDataForFigure1C.tif]

|     |   |     |   |   |     |   |          |
|-----|---|-----|---|---|-----|---|----------|
|     | - | -   | - | + | +   | + | ORF3c-HA |
| kDa | - | +++ | + | - | +++ | + | SeV      |

42

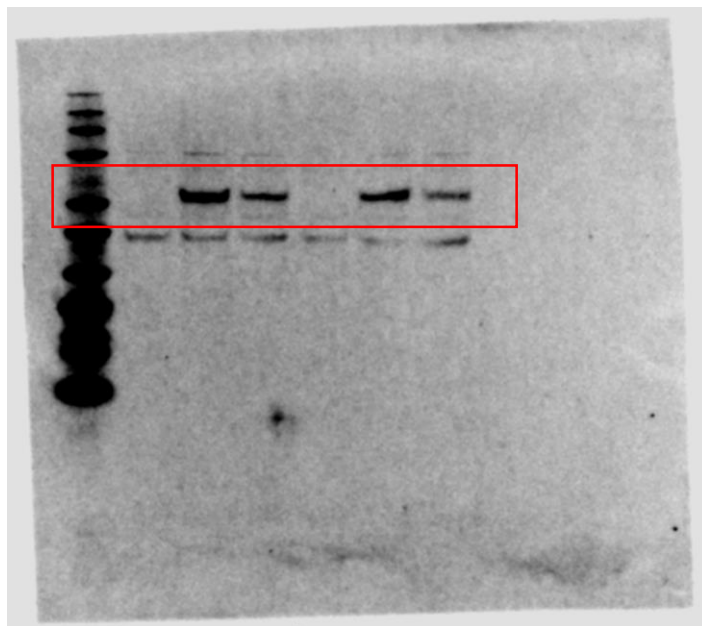

SeV

|     |   |     |   |   |     |   |          |
|-----|---|-----|---|---|-----|---|----------|
|     | - | -   | - | + | +   | + | ORF3c-HA |
| kDa | - | +++ | + | - | +++ | + | SeV      |

42  
31

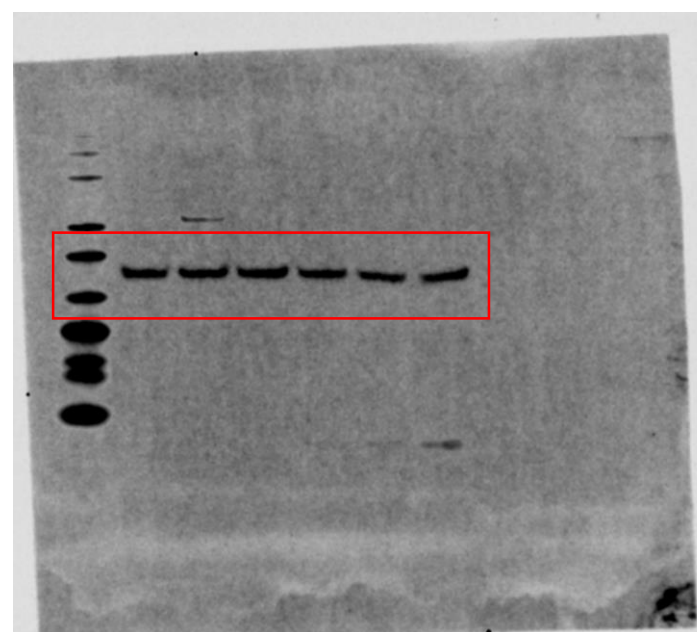

GAPDH

kDa

8

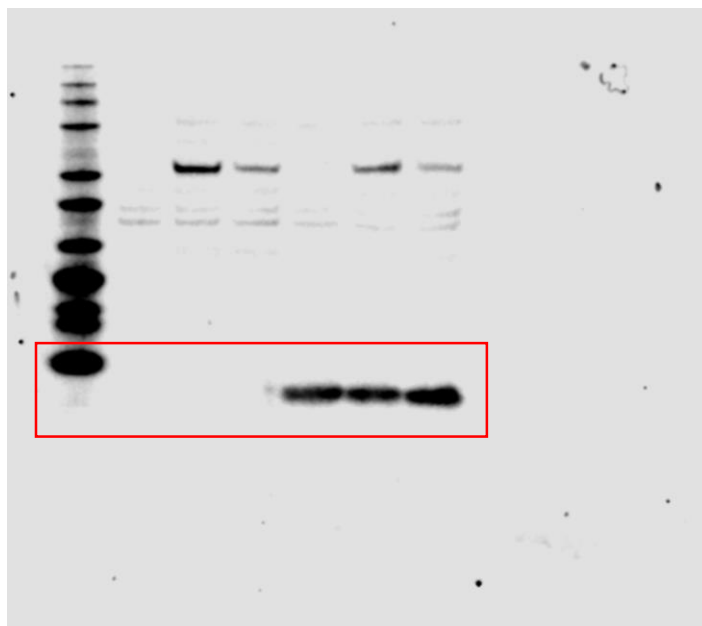

HA

Supplement: Supplementary file 4 — Source Data for Figure 1 [file EMBR-24-e57137-s003.zip › Fig1E/EMBOR-2023-57137V2_SourceDataForFigure1E.pdf]

## Slide 1
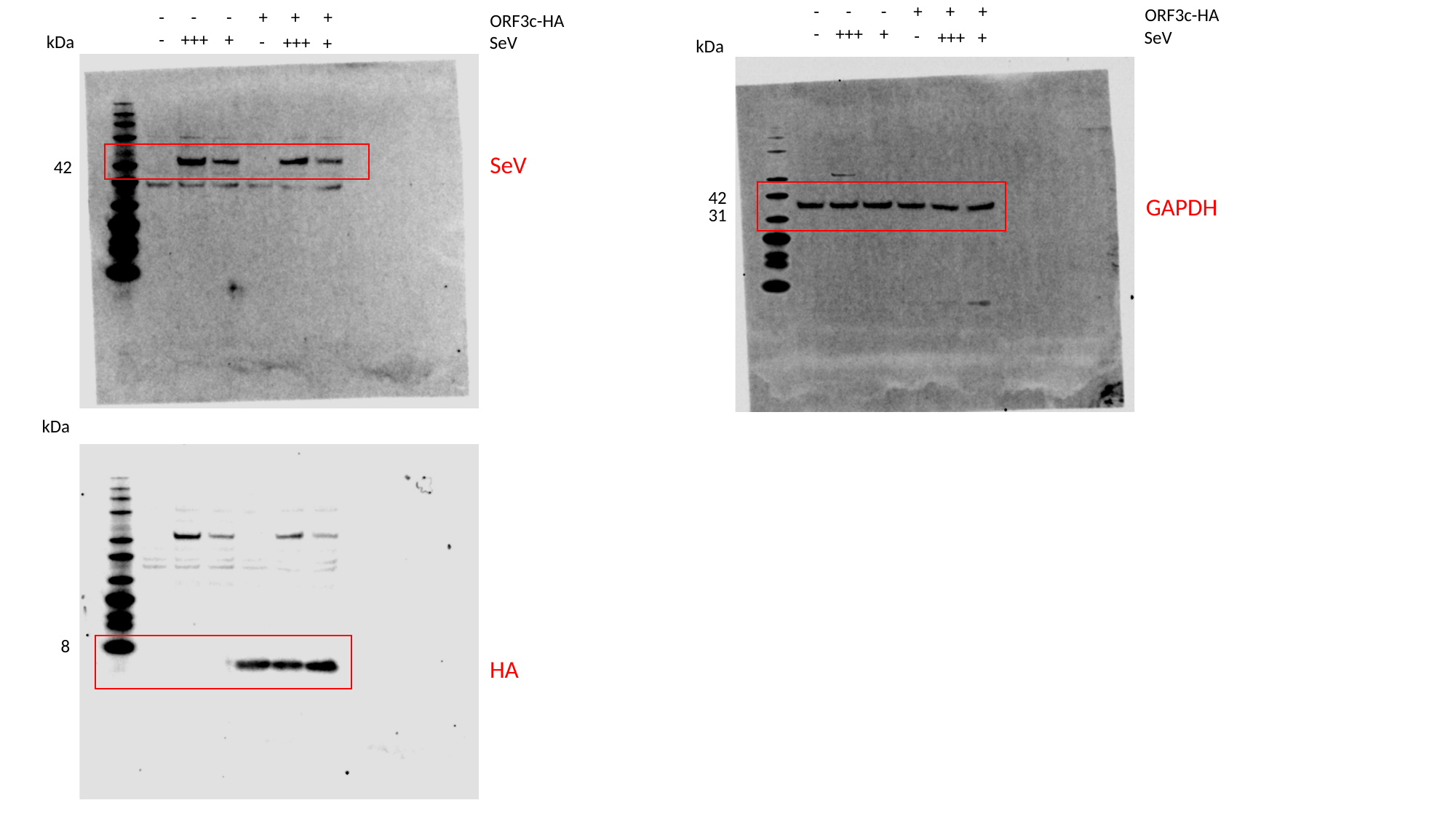

-
-
-
+
+
+
ORF3c-HA
-
-
-
+
+
+
ORF3c-HA
-
+++
+
-
+++
SeV
+
-
+++
+
kDa
-
+++
SeV
+
kDa
SeV
42
42
GAPDH
31
kDa
8
HA

Supplement: Supplementary file 4 — Source Data for Figure 1 [file EMBR-24-e57137-s003.zip › Fig1E/EMBOR-2023-57137V2_SourceDataForFigure1E.pptx]

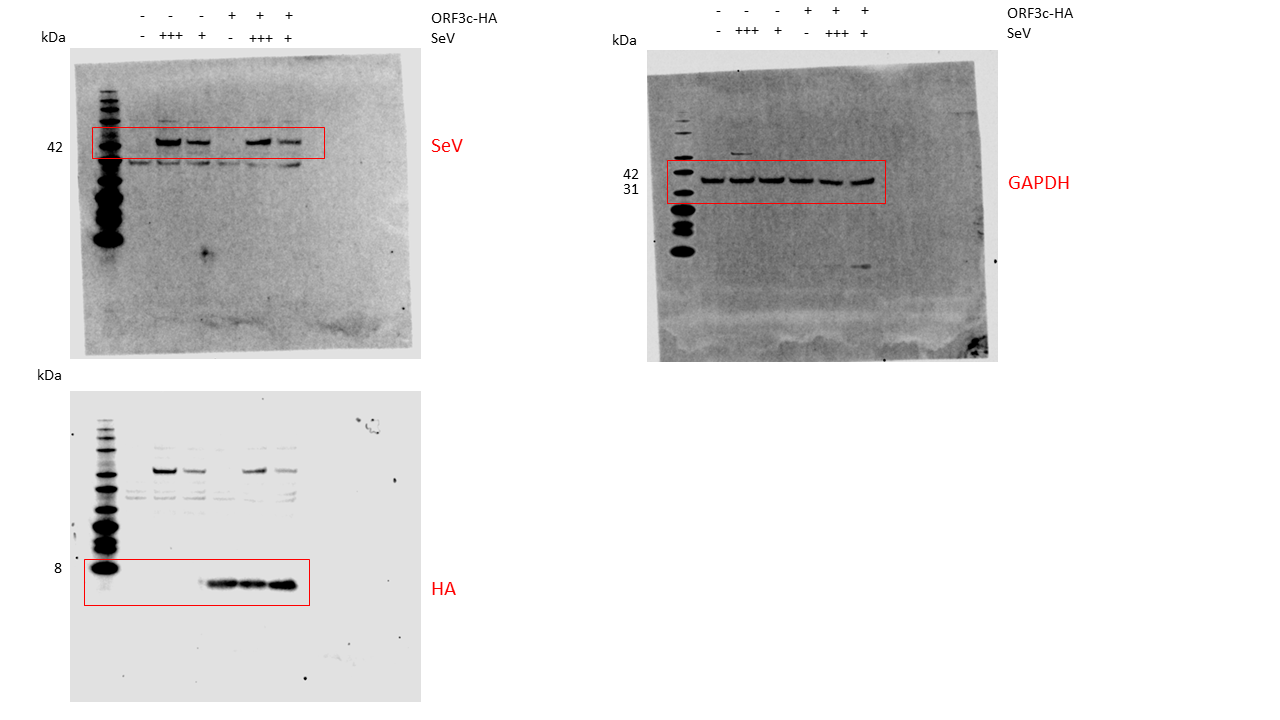

Supplement: Supplementary file 4 — Source Data for Figure 1 [file EMBR-24-e57137-s003.zip › Fig1E/EMBOR-2023-57137V2_SourceDataForFigure1E.tif]

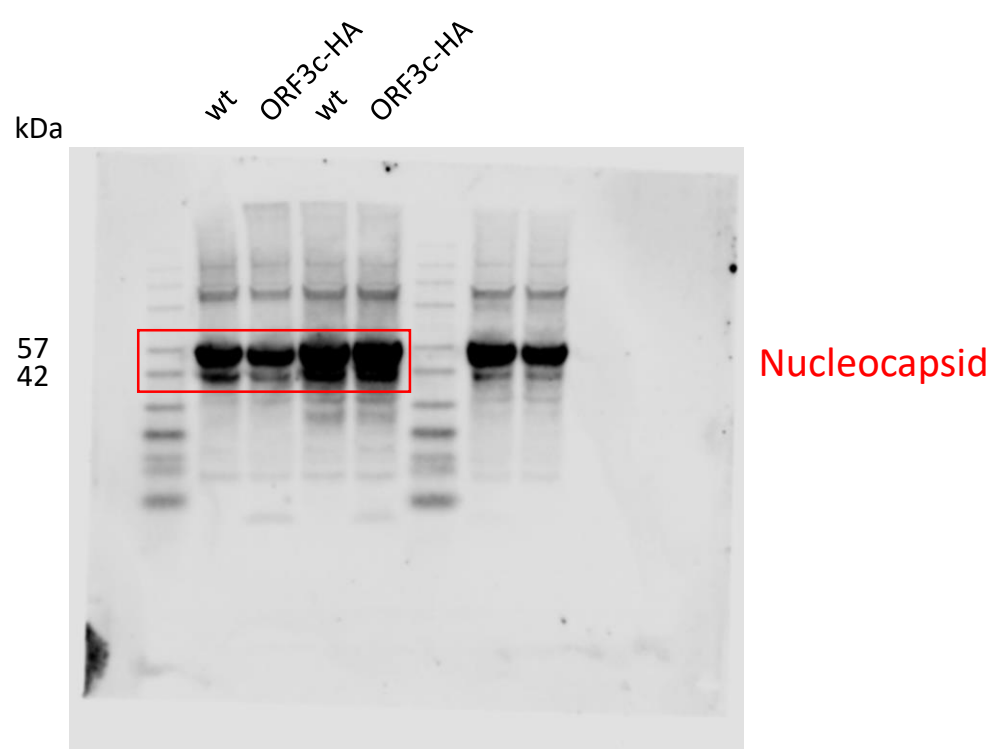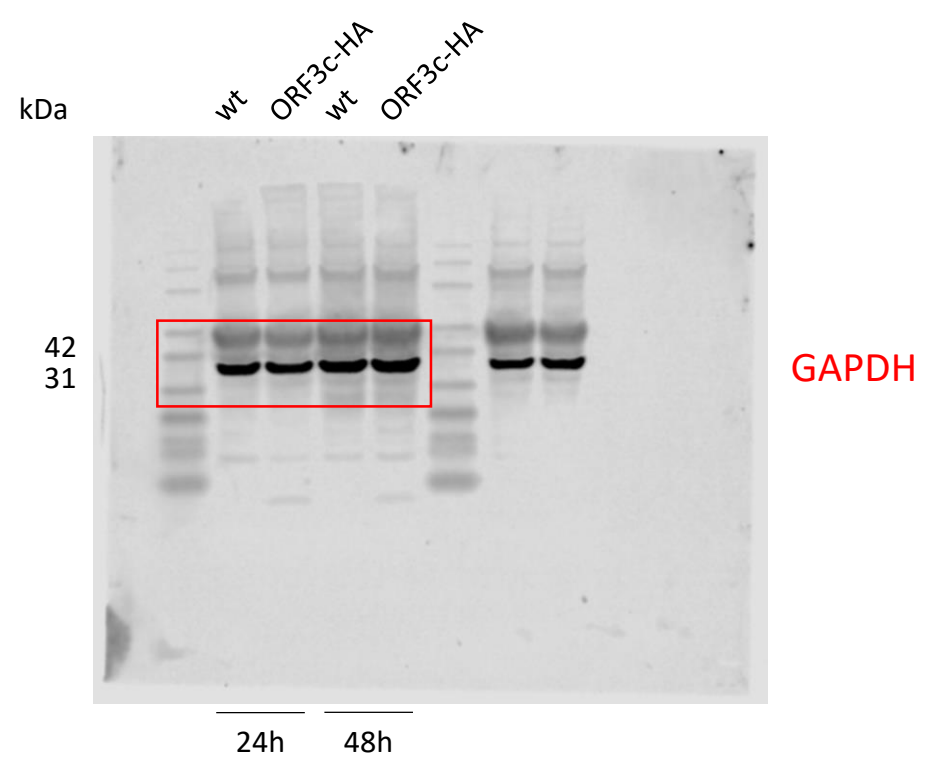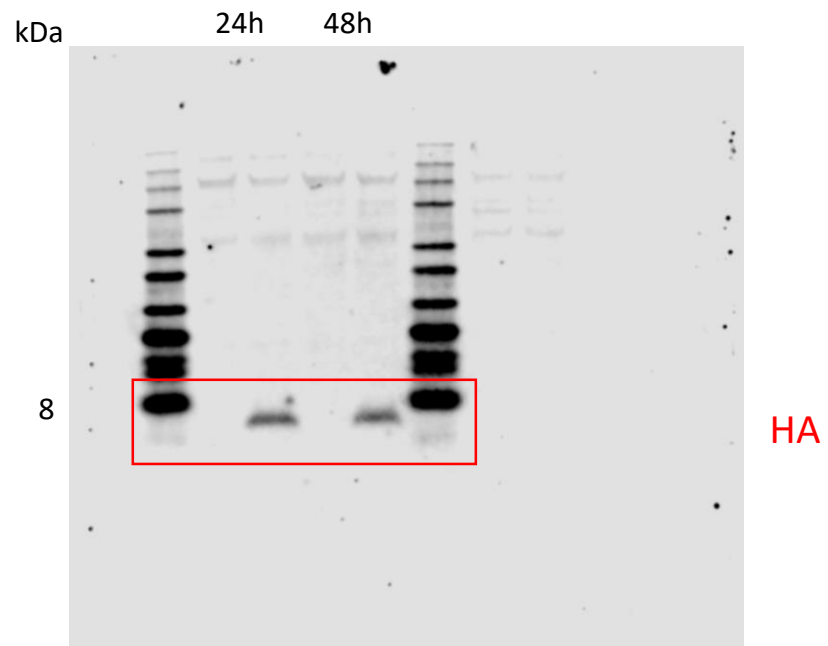

Supplement: Supplementary file 4 — Source Data for Figure 1 [file EMBR-24-e57137-s003.zip › Fig1F/EMBOR-2023-57137V2_SourceDataForFigure1F.pdf]

## Slide 1
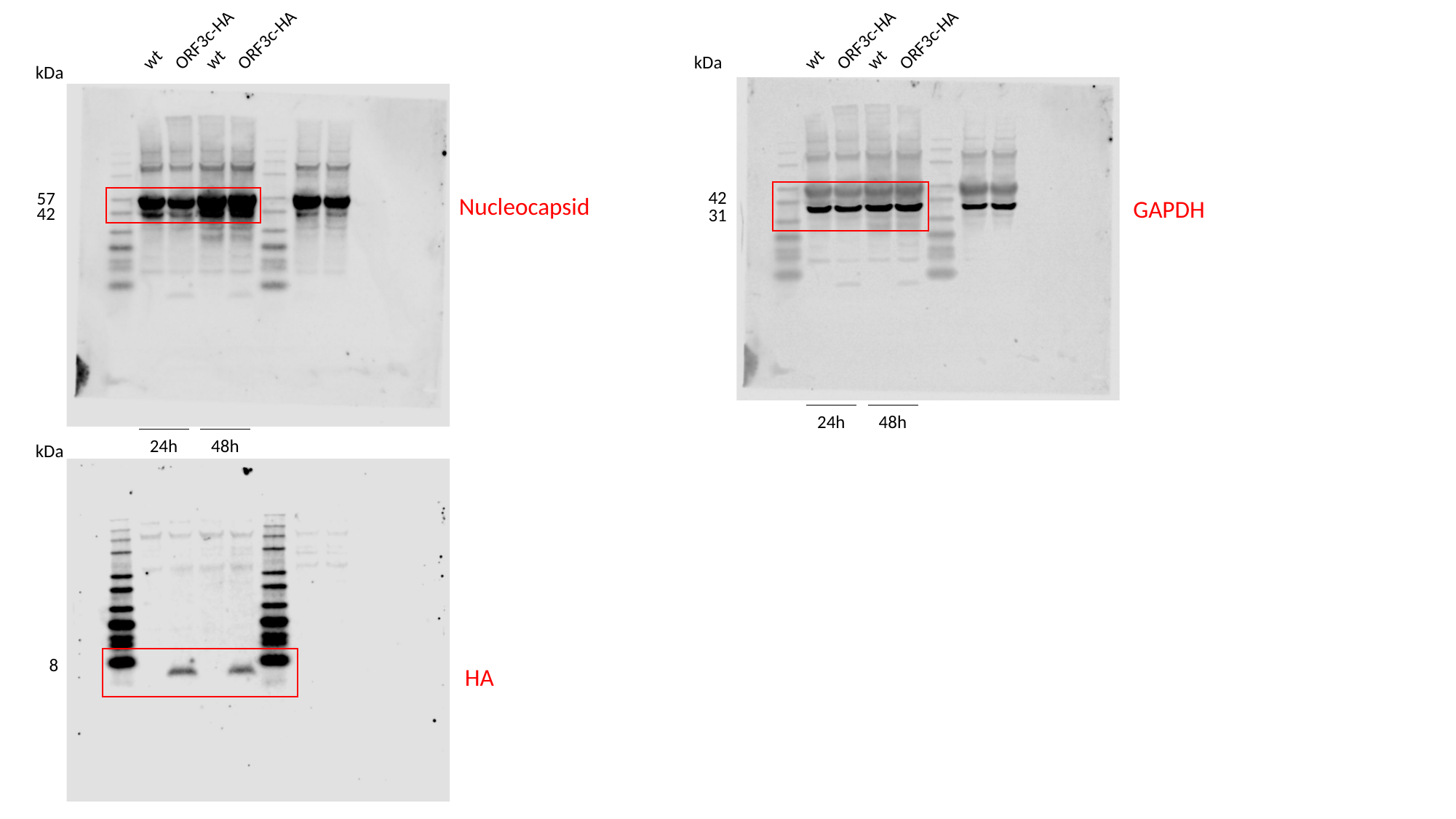

ORF3c-HA
ORF3c-HA
ORF3c-HA
ORF3c-HA
wt
wt
wt
wt
kDa
kDa
42
57
Nucleocapsid
GAPDH
42
31
24h
48h
24h
48h
kDa
8
HA

Supplement: Supplementary file 4 — Source Data for Figure 1 [file EMBR-24-e57137-s003.zip › Fig1F/EMBOR-2023-57137V2_SourceDataForFigure1F.pptx]

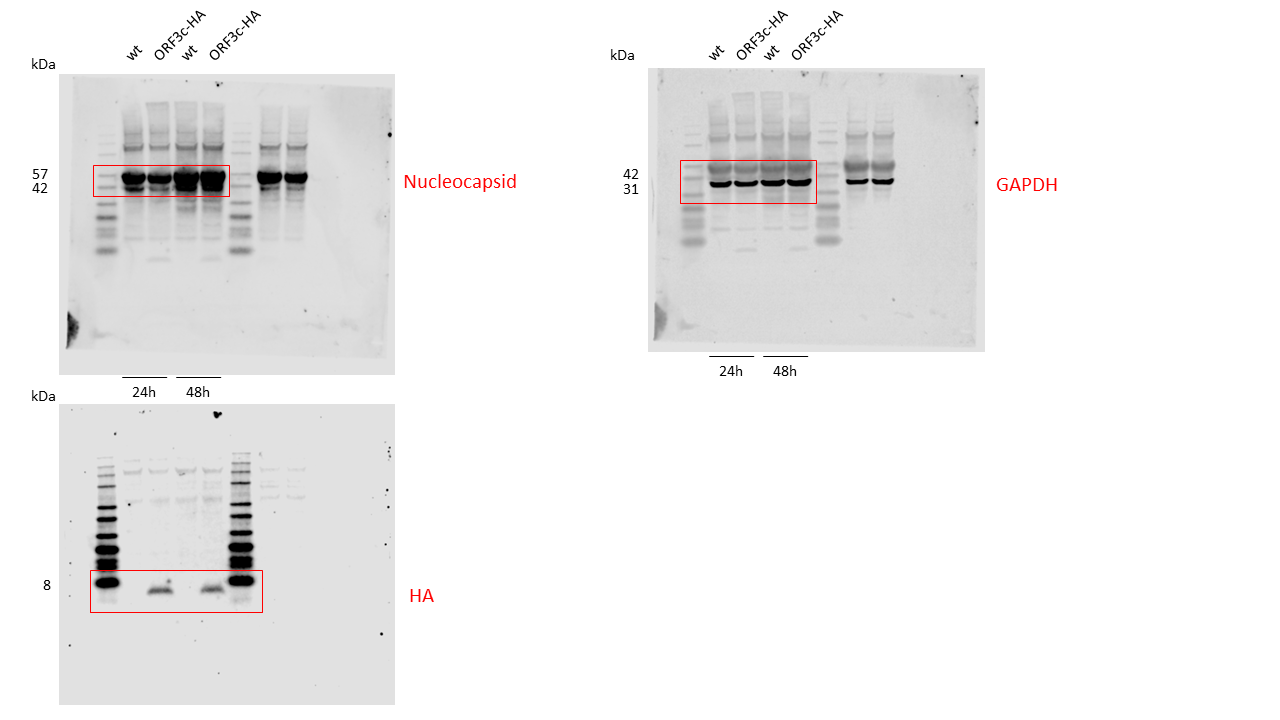

Supplement: Supplementary file 4 — Source Data for Figure 1 [file EMBR-24-e57137-s003.zip › Fig1F/EMBOR-2023-57137V2_SourceDataForFigure1F.tif]

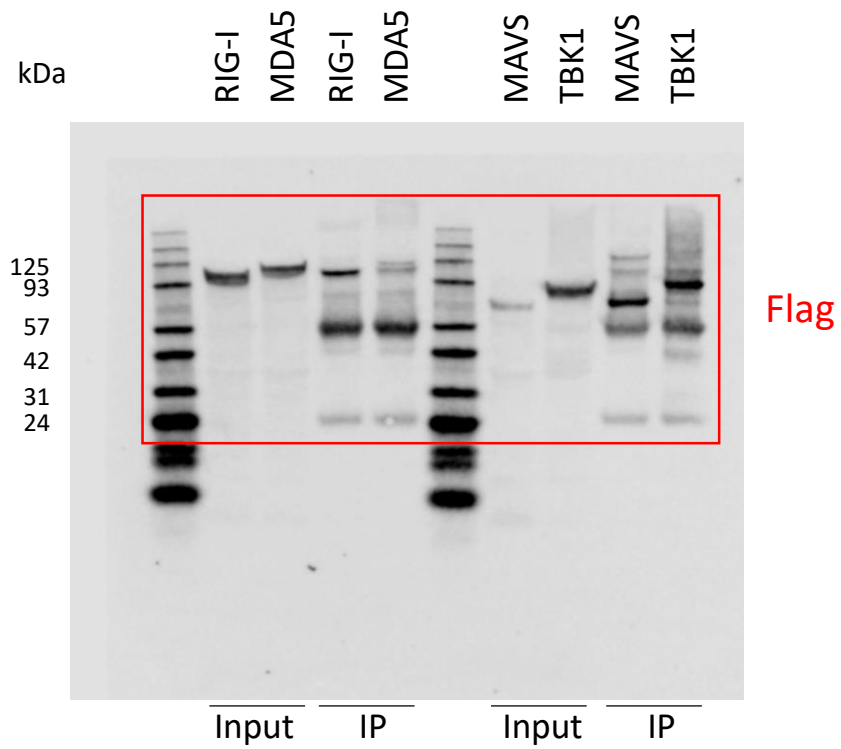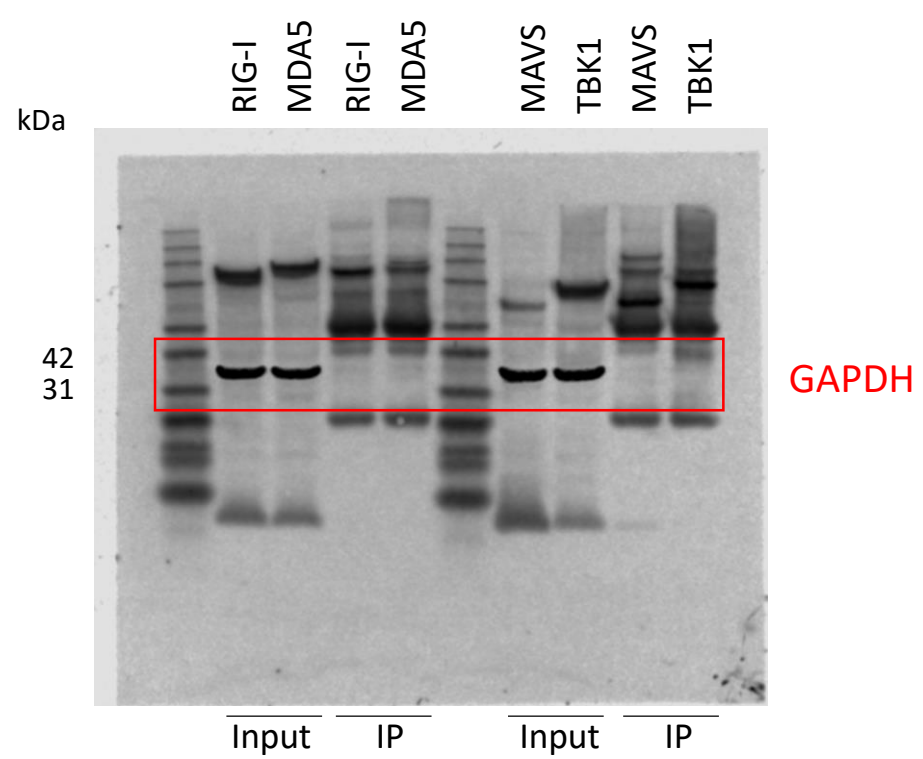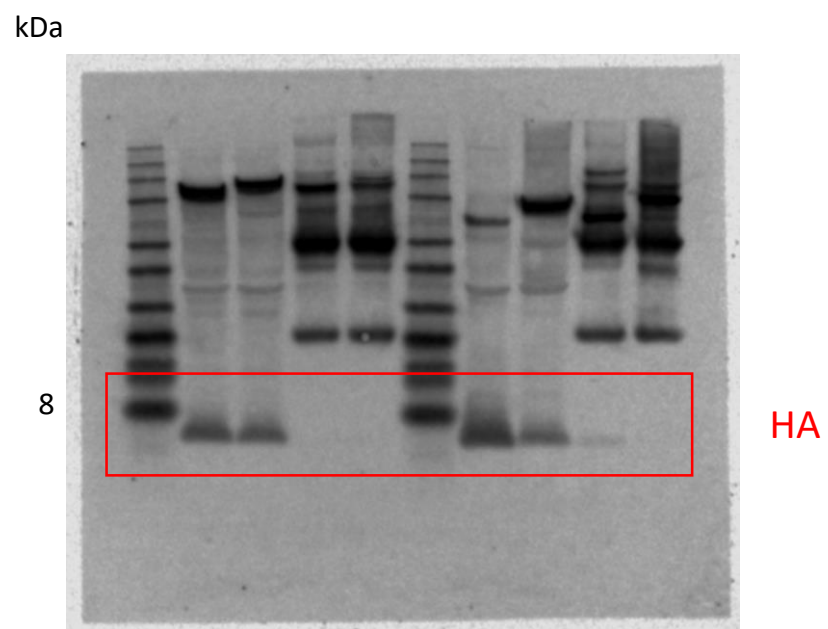

Supplement: Supplementary file 5 — Source Data for Figure 2 [file EMBR-24-e57137-s001.zip › Fig2D/EMBOR-2023-57137V2_SourceDataForFigure2D.pdf]

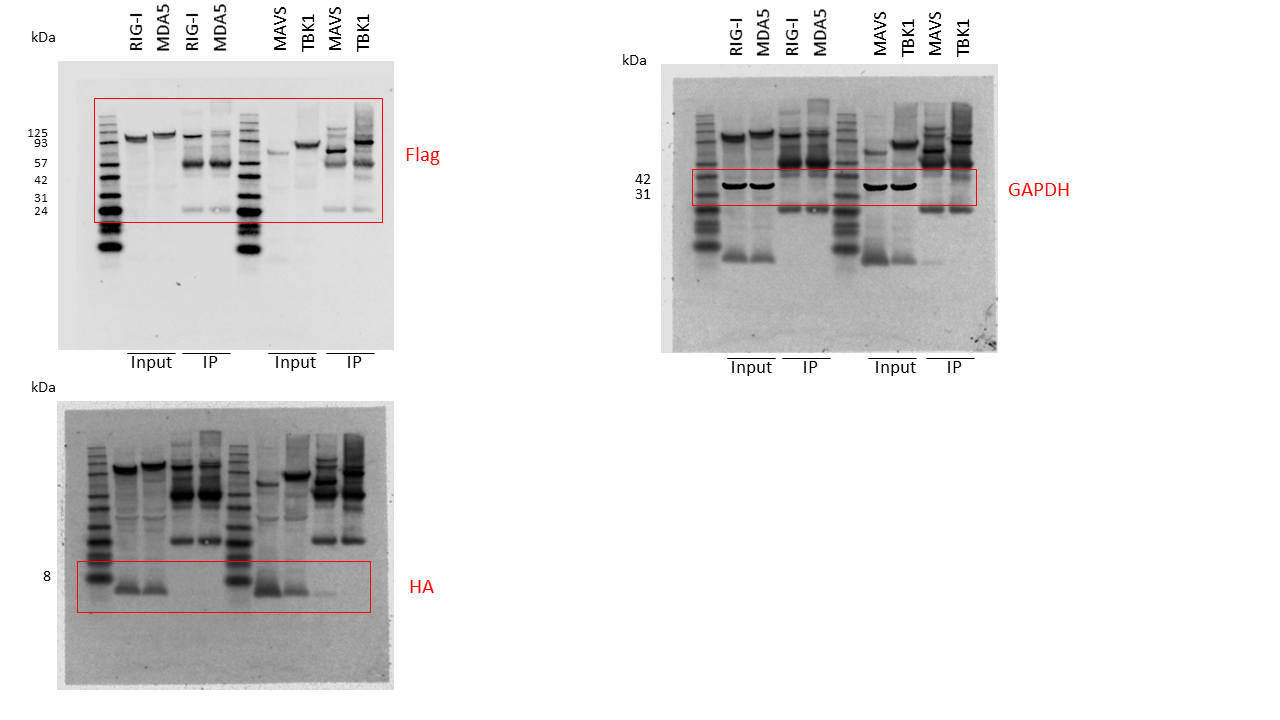

Supplement: Supplementary file 5 — Source Data for Figure 2 [file EMBR-24-e57137-s001.zip › Fig2D/EMBOR-2023-57137V2_SourceDataForFigure2D.tif]

## Slide 1
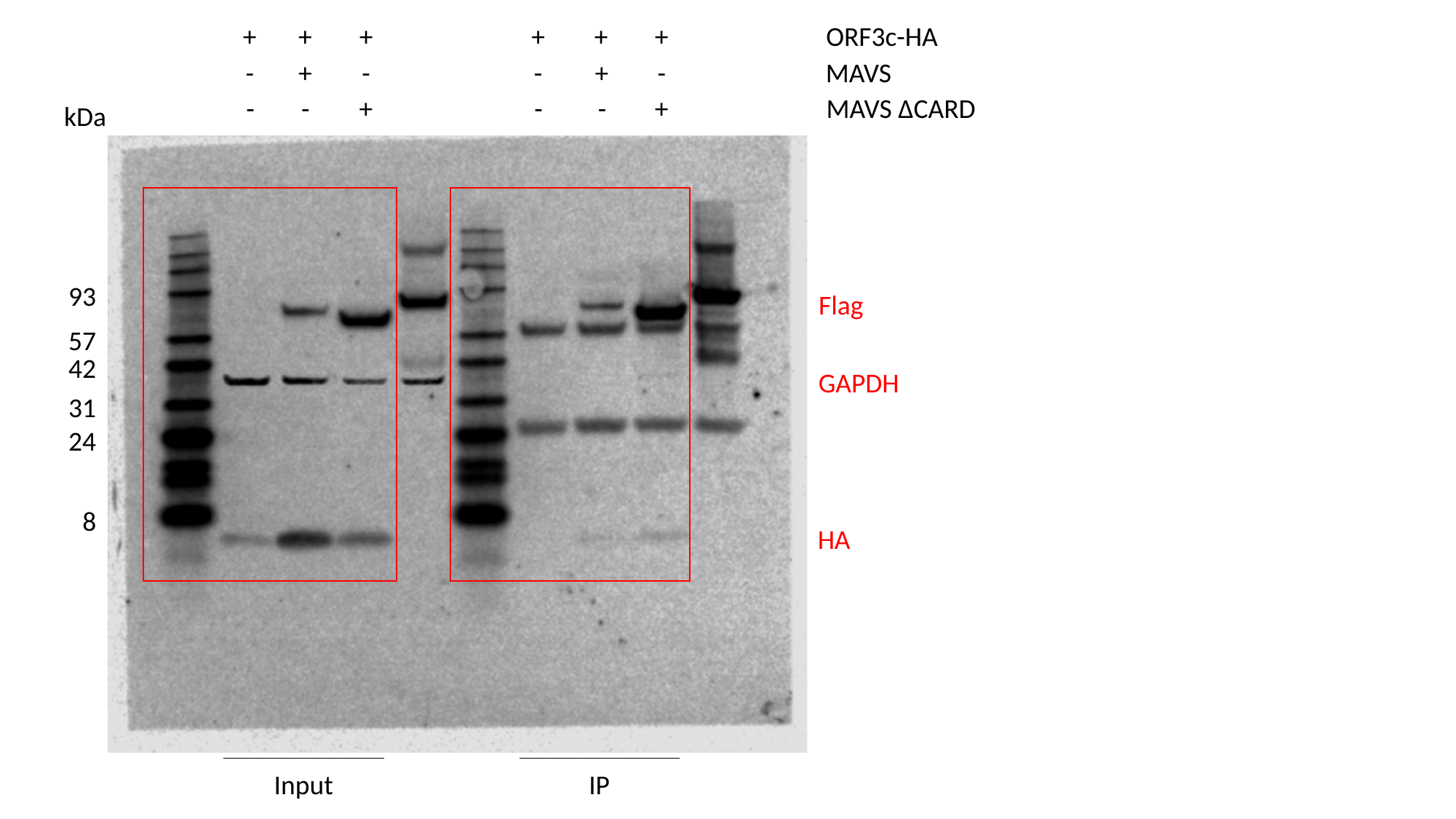

+
+
+
+
+
+
ORF3c-HA
-
+
-
-
+
-
MAVS
-
-
+
-
-
+
MAVS ΔCARD
kDa
93
Flag
57
42
GAPDH
31
24
8
HA
Input
IP

Supplement: Supplementary file 5 — Source Data for Figure 2 [file EMBR-24-e57137-s001.zip › Fig2E/EMBOR-2023-57137V2_SourceDataForFigure2E.pptx]

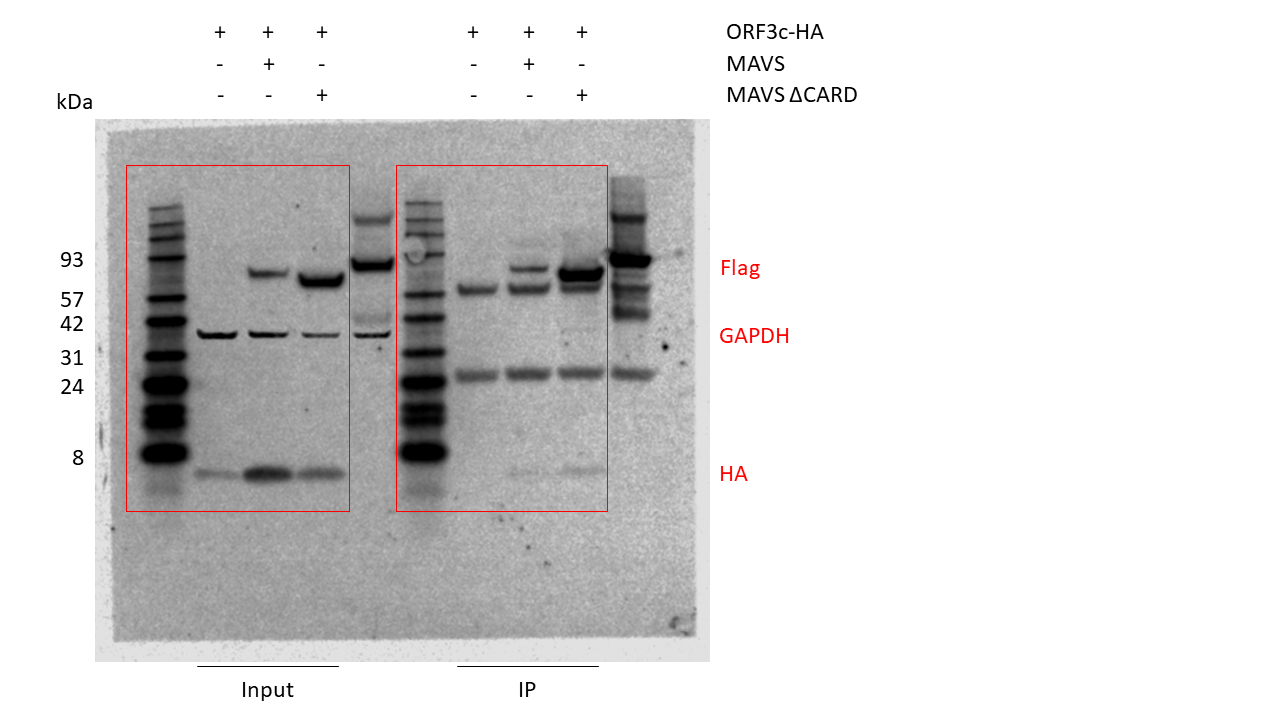

Supplement: Supplementary file 5 — Source Data for Figure 2 [file EMBR-24-e57137-s001.zip › Fig2E/EMBOR-2023-57137V2_SourceDataForFigure2E.tif]

kDa

- + ++ +++

ORF3c-HA

93

57

42

31

24

8

MAVS

GAPDH

Cleaved MAVS  
HA

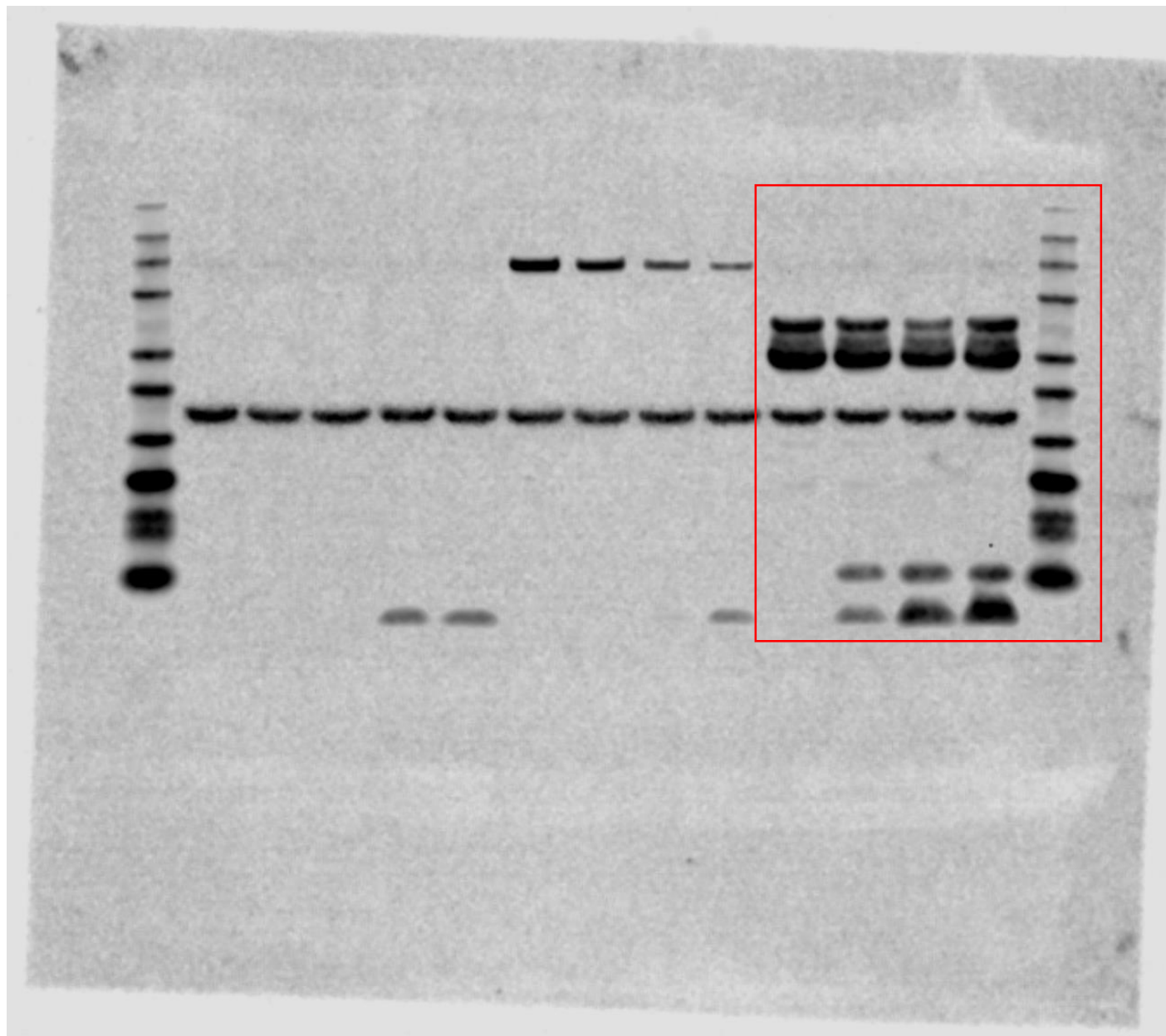

Supplement: Supplementary file 5 — Source Data for Figure 2 [file EMBR-24-e57137-s001.zip › Fig2G/EMBOR-2023-57137V2_SourceDataForFigure2G.pdf]

## Slide 1
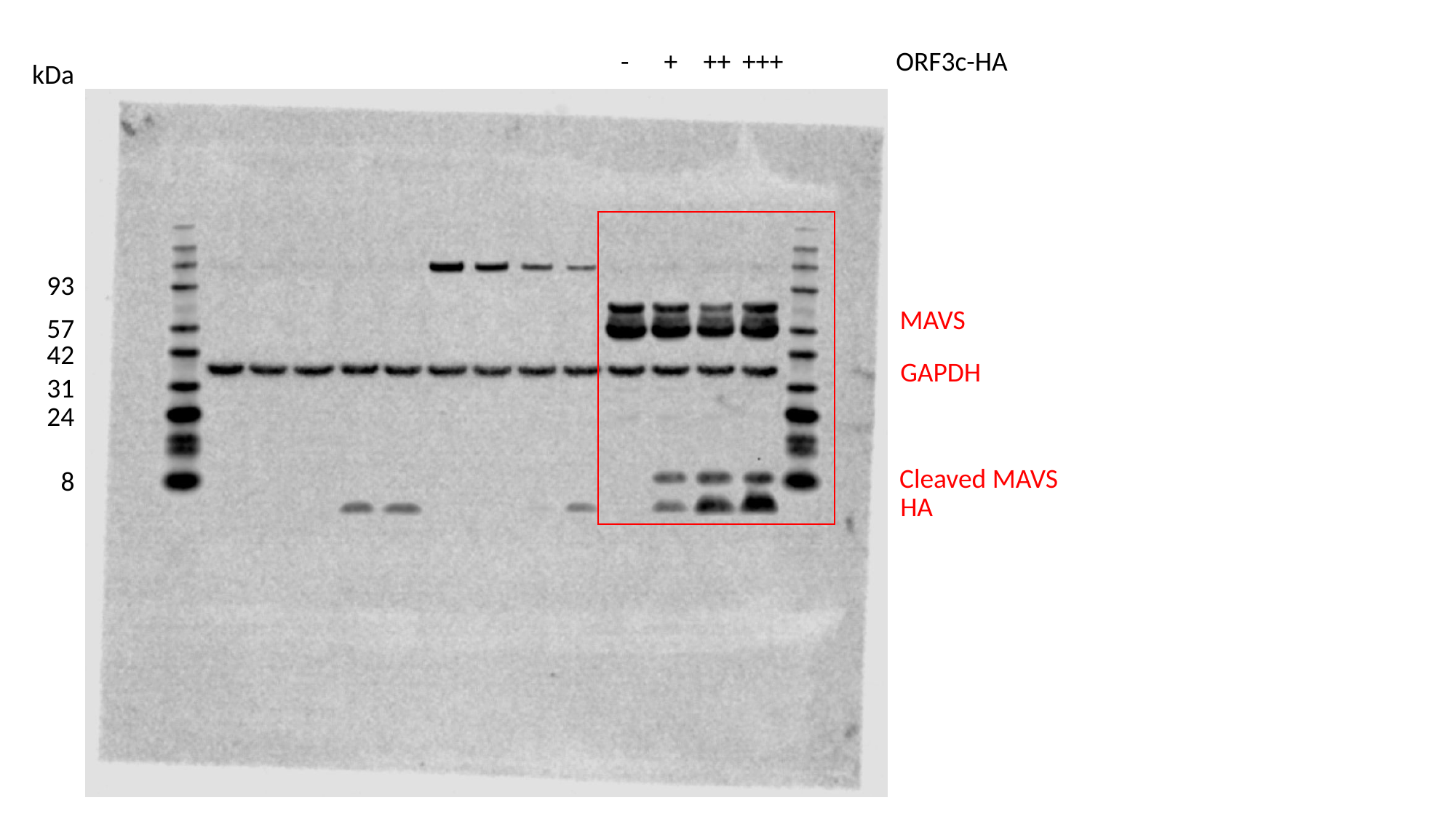

-
+
++
+++
ORF3c-HA
kDa
93
MAVS
57
42
GAPDH
31
24
Cleaved MAVS
8
HA

Supplement: Supplementary file 5 — Source Data for Figure 2 [file EMBR-24-e57137-s001.zip › Fig2G/EMBOR-2023-57137V2_SourceDataForFigure2G.pptx]

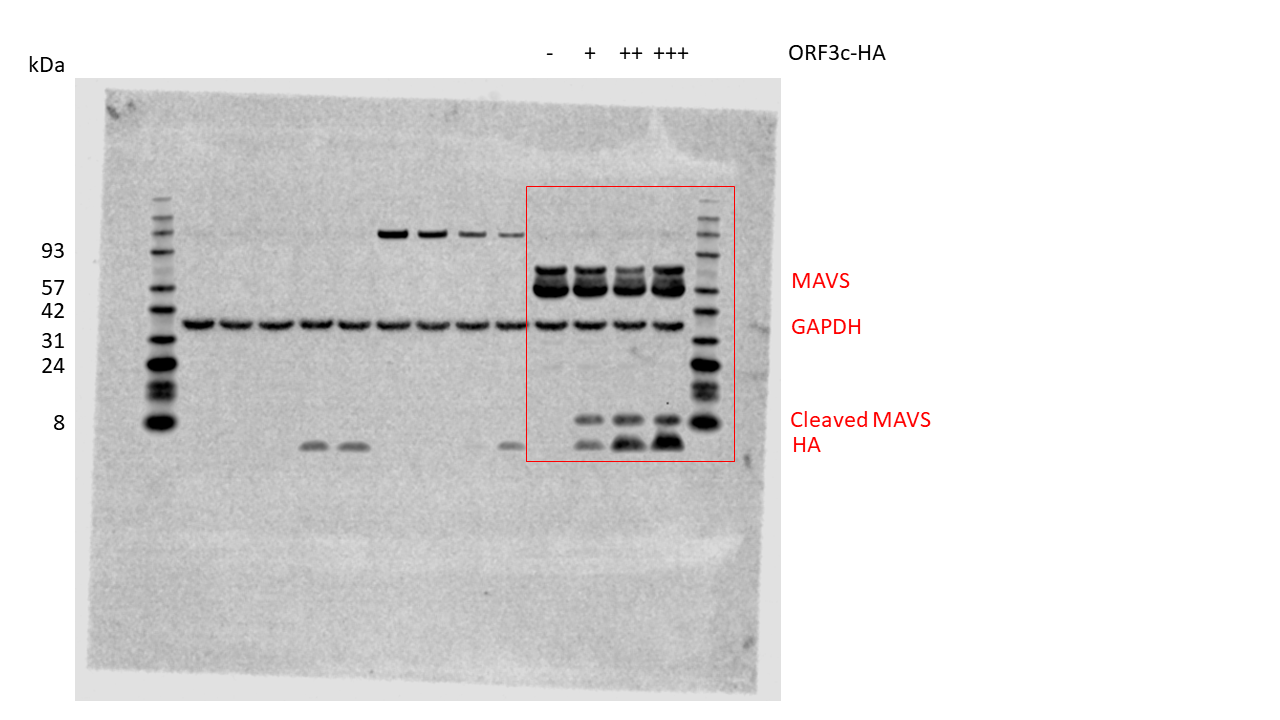

Supplement: Supplementary file 5 — Source Data for Figure 2 [file EMBR-24-e57137-s001.zip › Fig2G/EMBOR-2023-57137V2_SourceDataForFigure2G.tif]

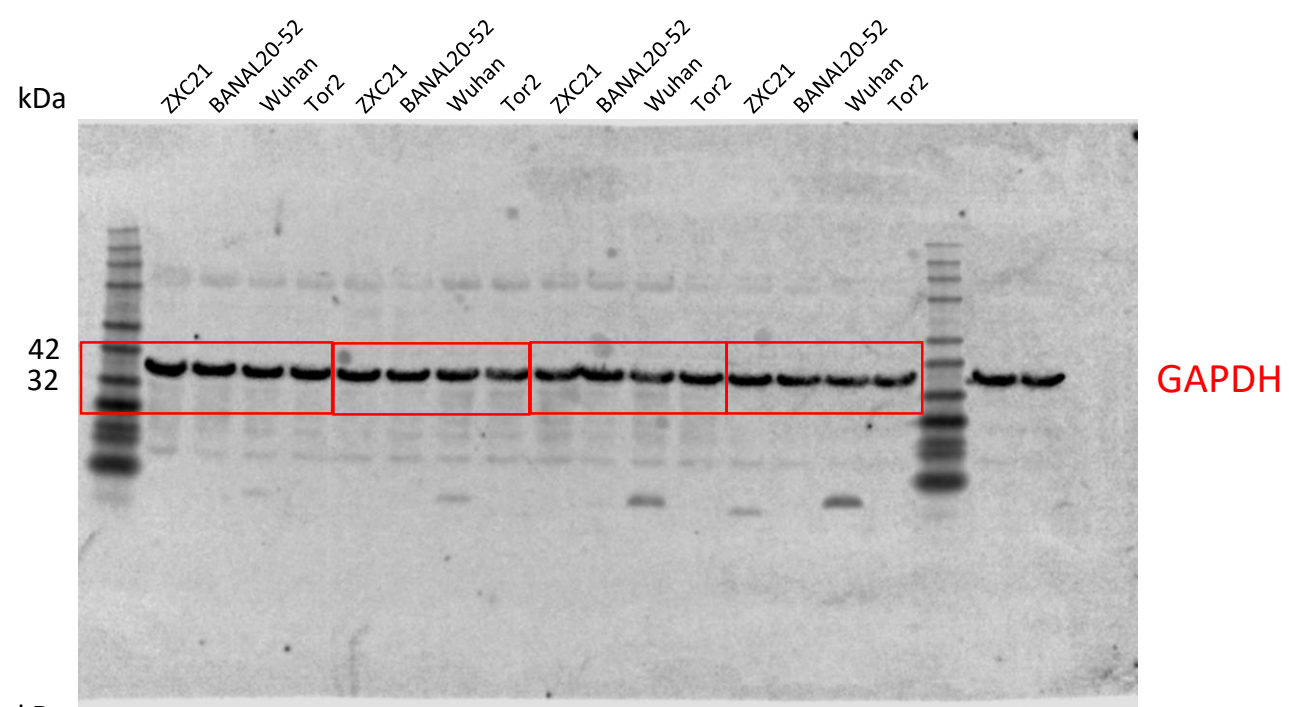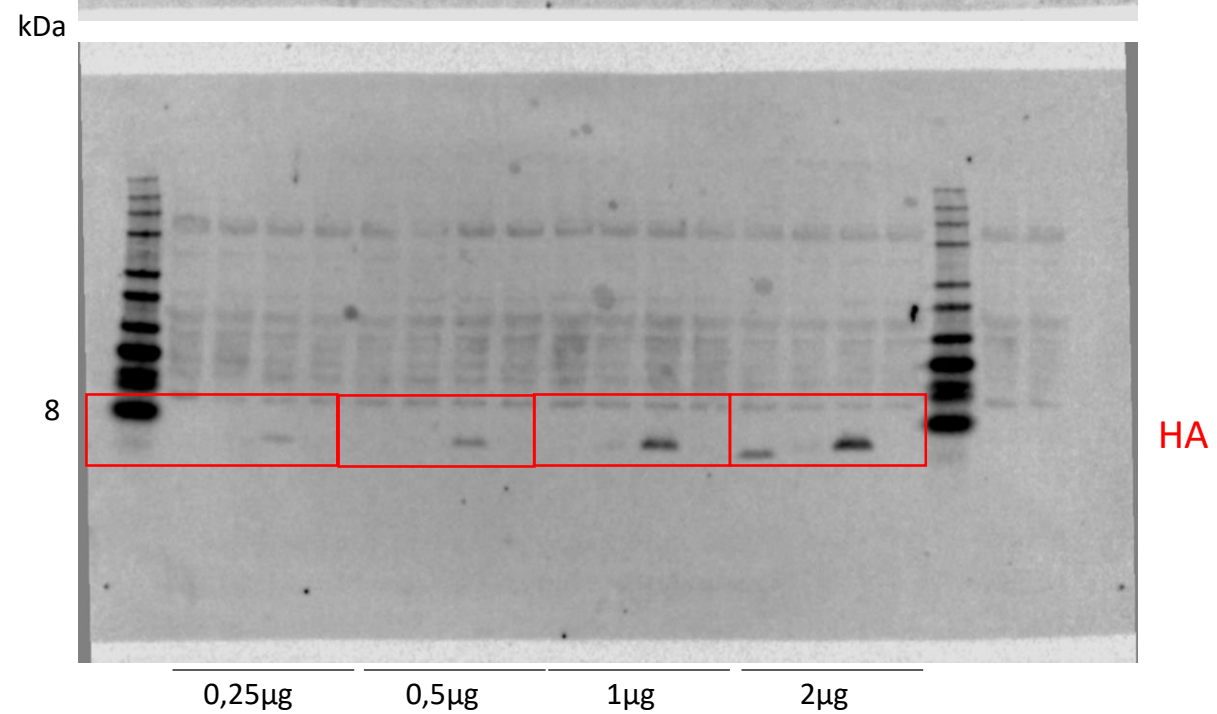

Supplement: Supplementary file 6 — Source Data for Figure 3 [file EMBR-24-e57137-s005.zip › Fig3C/EMBOR-2023-57137V2_SourceDataForFigure3C.pdf]

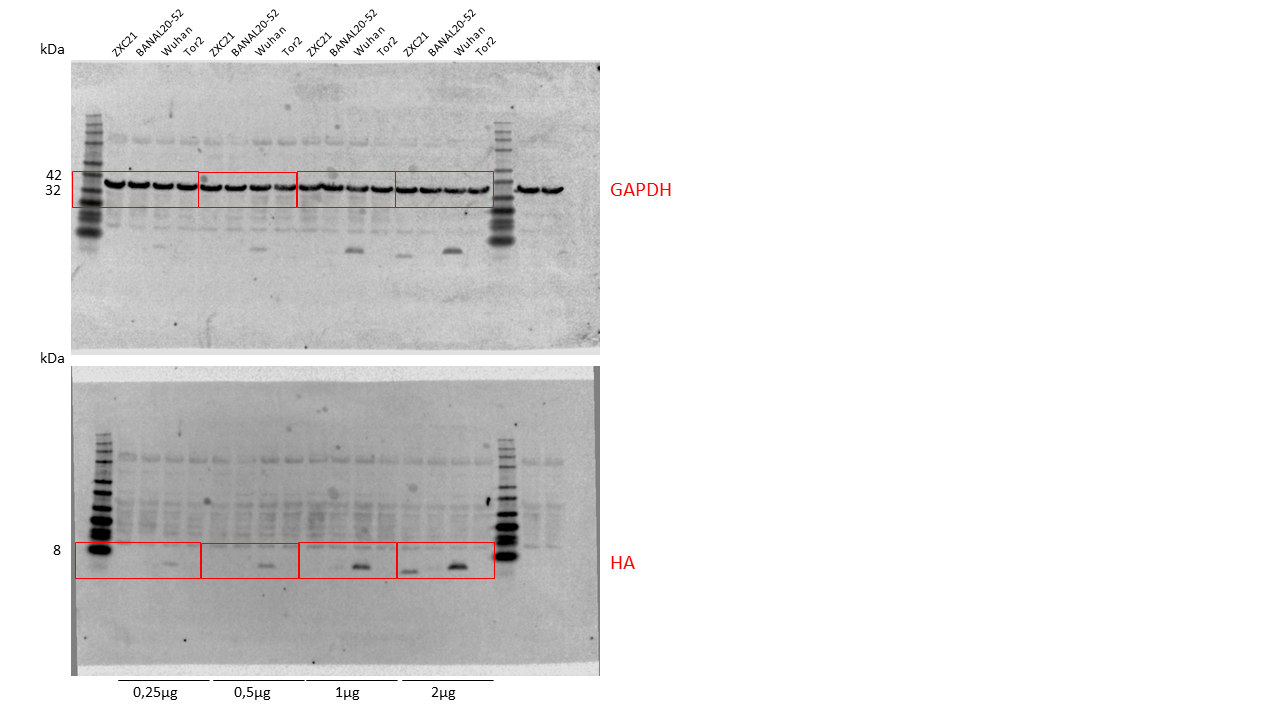

Supplement: Supplementary file 6 — Source Data for Figure 3 [file EMBR-24-e57137-s005.zip › Fig3C/EMBOR-2023-57137V2_SourceDataForFigure3C.tif]

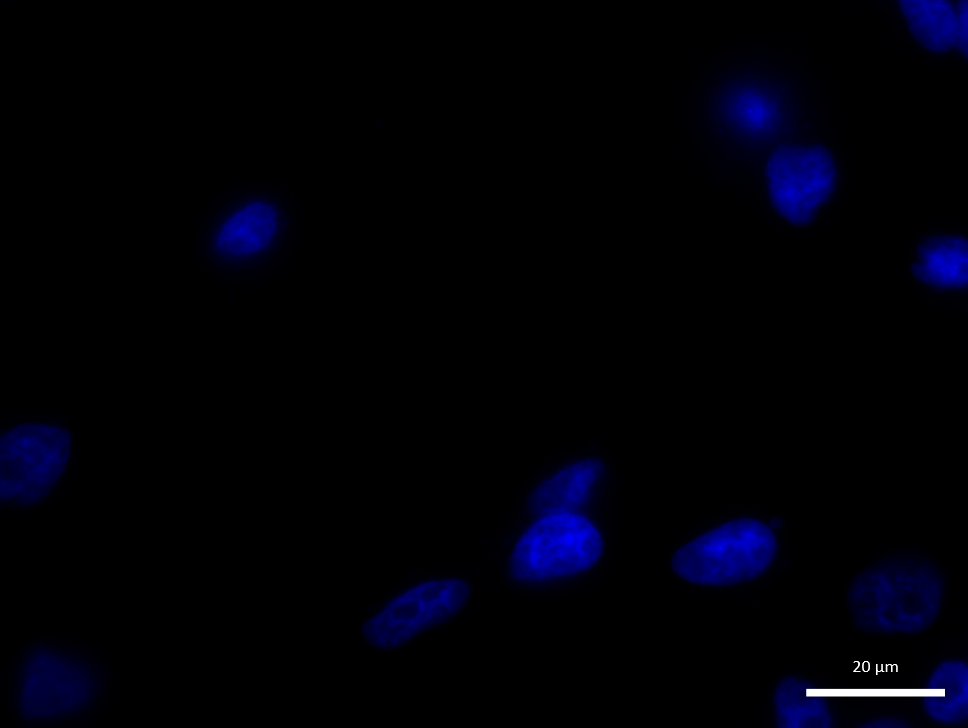

Supplement: Supplementary file 7 — Source Data for Figure 4 [file EMBR-24-e57137-s009.zip › Fig4D/Mock blue green.tif]

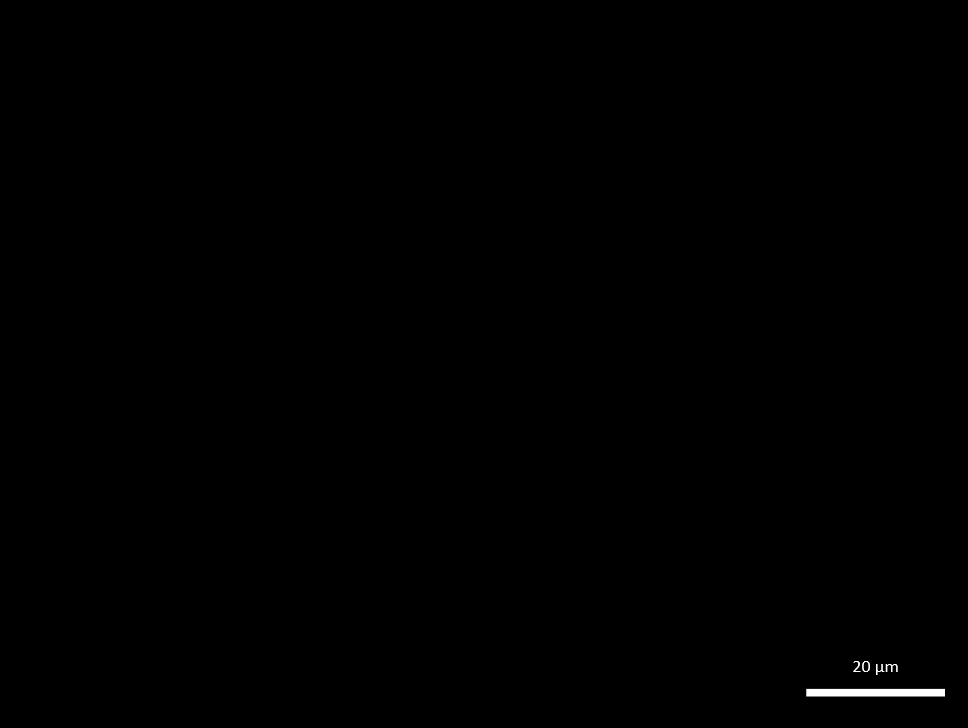

Supplement: Supplementary file 7 — Source Data for Figure 4 [file EMBR-24-e57137-s009.zip › Fig4D/Mock green.tif]

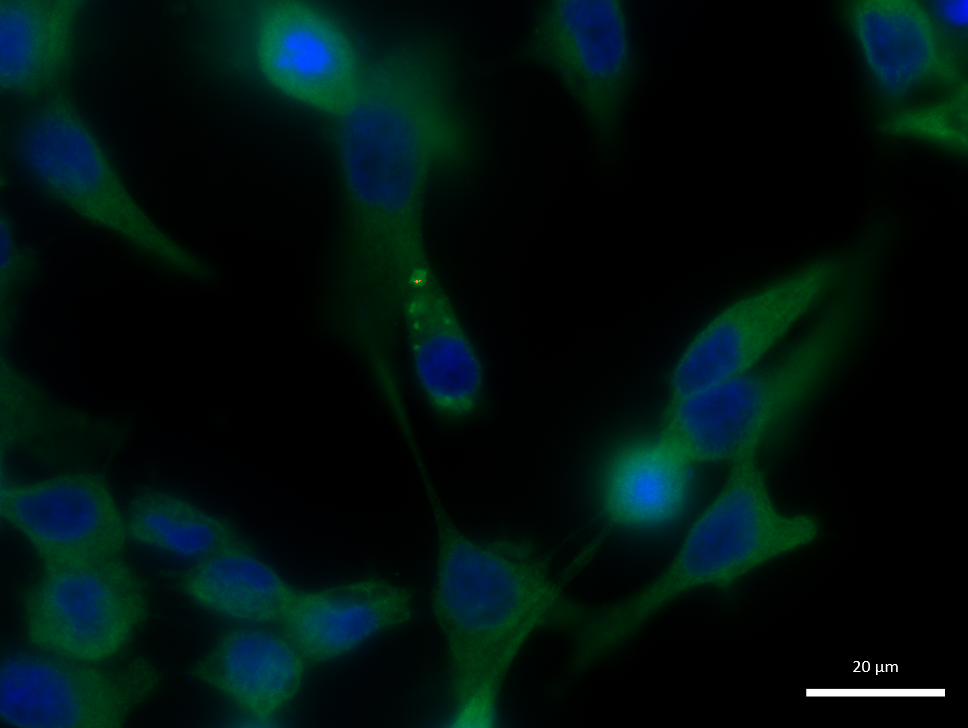

Supplement: Supplementary file 7 — Source Data for Figure 4 [file EMBR-24-e57137-s009.zip › Fig4D/ORF3c R36I green blue.tif]

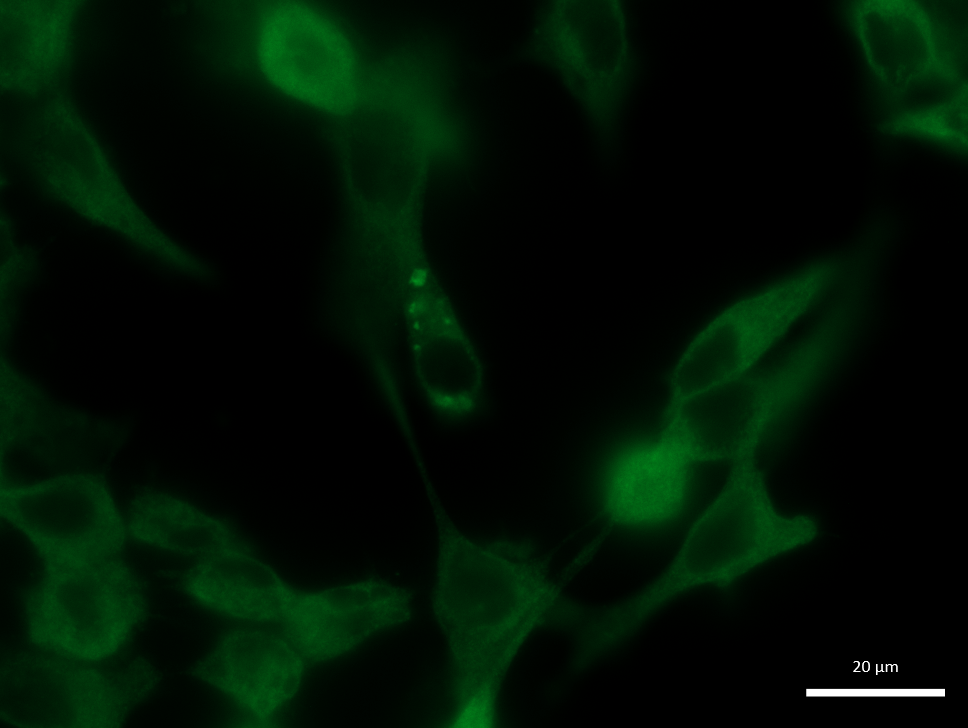

Supplement: Supplementary file 7 — Source Data for Figure 4 [file EMBR-24-e57137-s009.zip › Fig4D/ORF3c R36I green.tif]

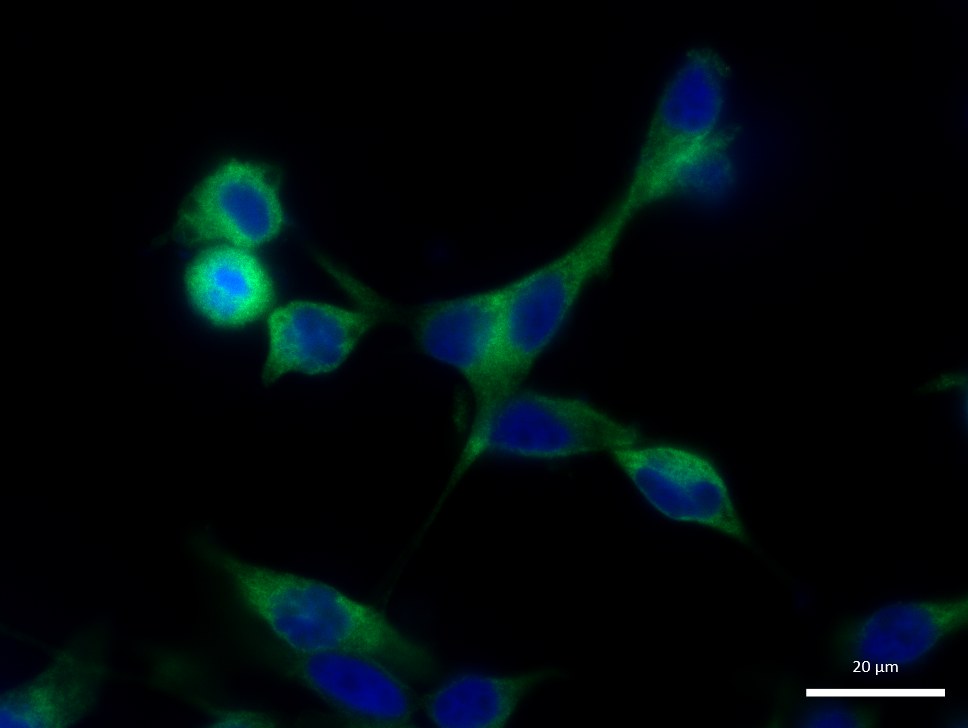

Supplement: Supplementary file 7 — Source Data for Figure 4 [file EMBR-24-e57137-s009.zip › Fig4D/ORF3c wt blue green.tif]

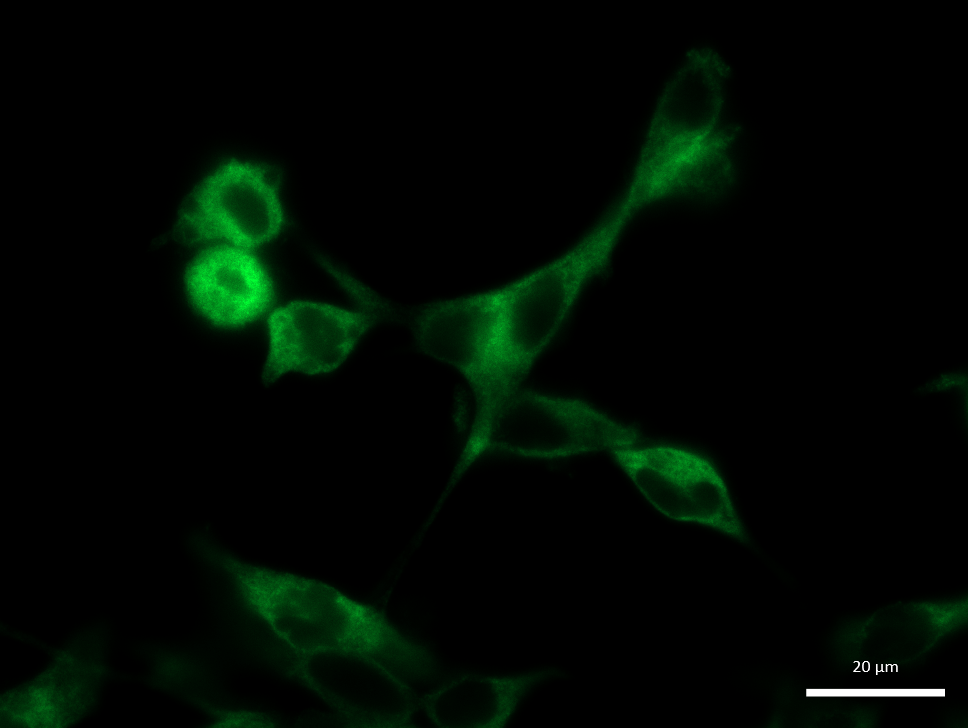

Supplement: Supplementary file 7 — Source Data for Figure 4 [file EMBR-24-e57137-s009.zip › Fig4D/ORF3c wt green.tif]
