# Supplementary material for: ORF3c is expressed in SARS‐CoV‐2‐infected cells and inhibits innate sensing by targeting MAVS
Source: EMBO Rep. 2023 Oct 23;24(12):e57137. doi: 10.15252/embr.202357137 (PMC10702836; doi:10.15252/embr.202357137)
Supplement: Supplementary file 5 — Source Data for Figure 2 [file EMBR-24-e57137-s001.zip › Fig2D/EMBOR-2023-57137V2_SourceDataForFigure2D.pptx]

## Slide 1
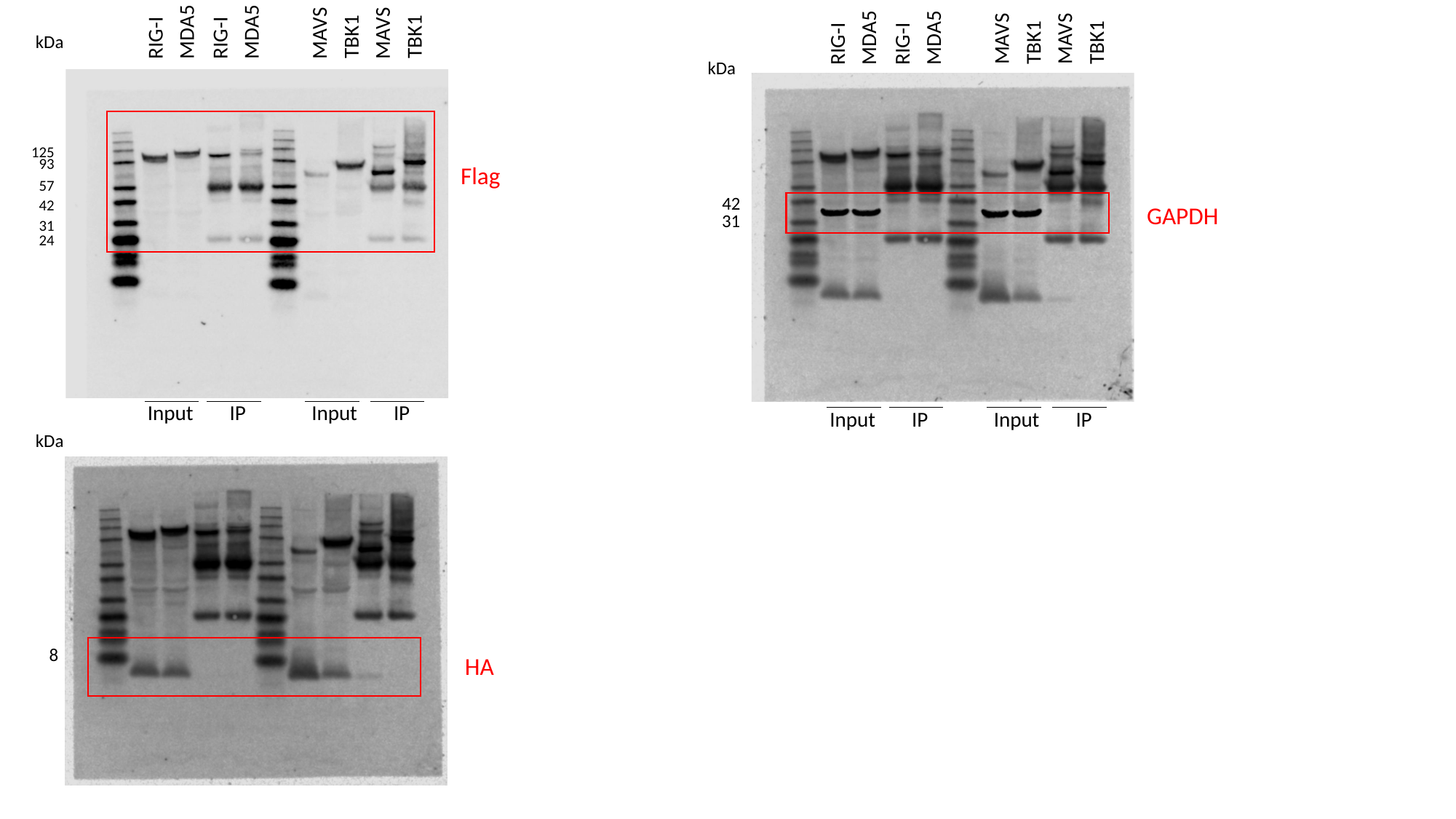

MDA5
MDA5
MAVS
MAVS
MDA5
MDA5
RIG-I
RIG-I
TBK1
TBK1
MAVS
MAVS
RIG-I
RIG-I
TBK1
TBK1
kDa
kDa
125
93
Flag
57
42
42
GAPDH
31
31
24
Input
IP
Input
IP
Input
IP
Input
IP
kDa
8
HA
