# Supplementary material for: ORF3c is expressed in SARS‐CoV‐2‐infected cells and inhibits innate sensing by targeting MAVS
Source: EMBO Rep. 2023 Oct 23;24(12):e57137. doi: 10.15252/embr.202357137 (PMC10702836; doi:10.15252/embr.202357137)
Supplement: Supplementary file 5 — Source Data for Figure 2 [file EMBR-24-e57137-s001.zip › Fig2E/EMBOR-2023-57137V2_SourceDataForFigure2E.pdf]

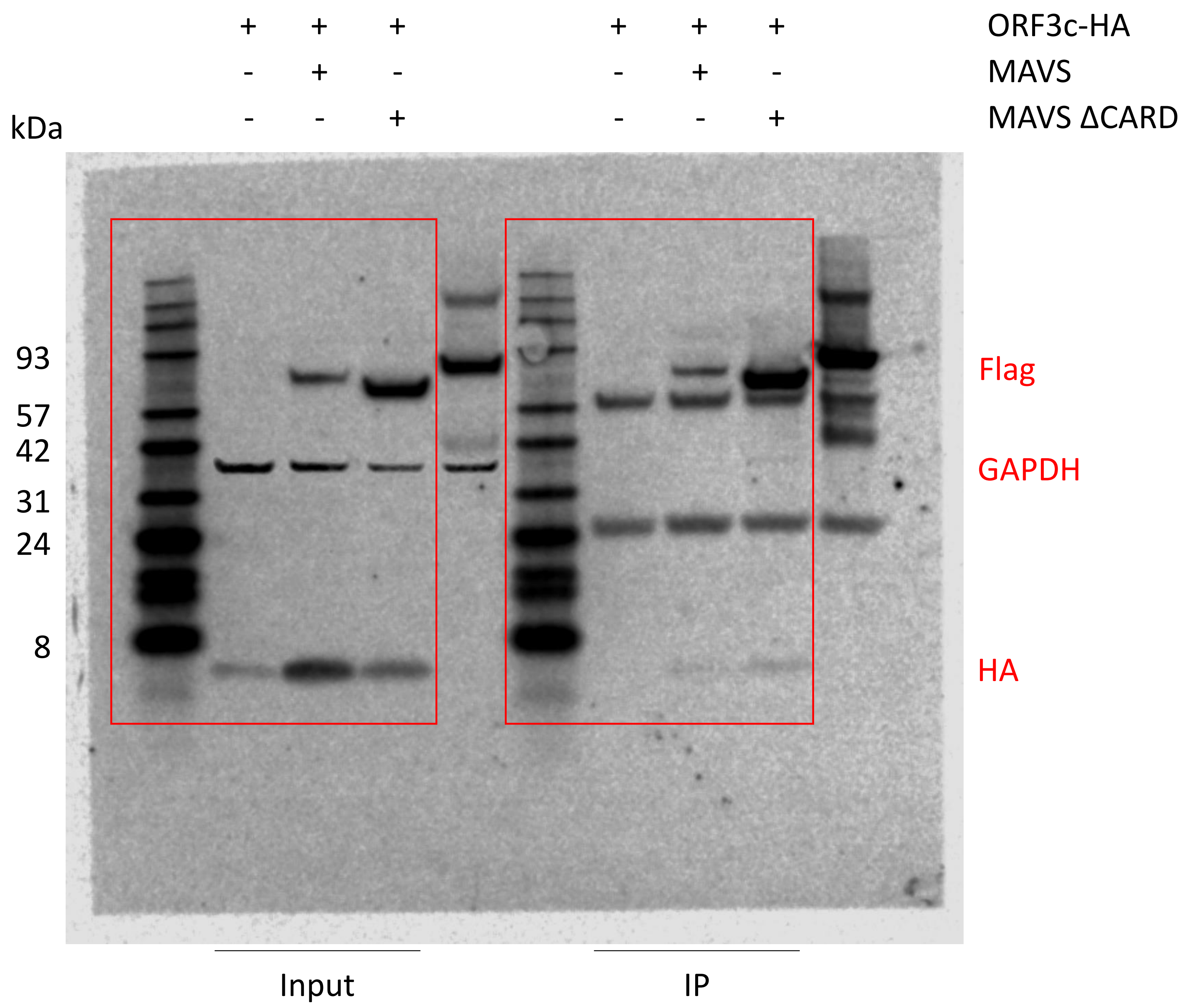

ORF3c-HA + + + + + +
MAVS - + - - + -
MAVS ΔCARD - - + - - +
kDa
93
57
42
31
24
8
Flag
GAPDH
HA
Input
IP
