# Supplementary material for: ORF3c is expressed in SARS‐CoV‐2‐infected cells and inhibits innate sensing by targeting MAVS
Source: EMBO Rep. 2023 Oct 23;24(12):e57137. doi: 10.15252/embr.202357137 (PMC10702836; doi:10.15252/embr.202357137)

## Slide 1
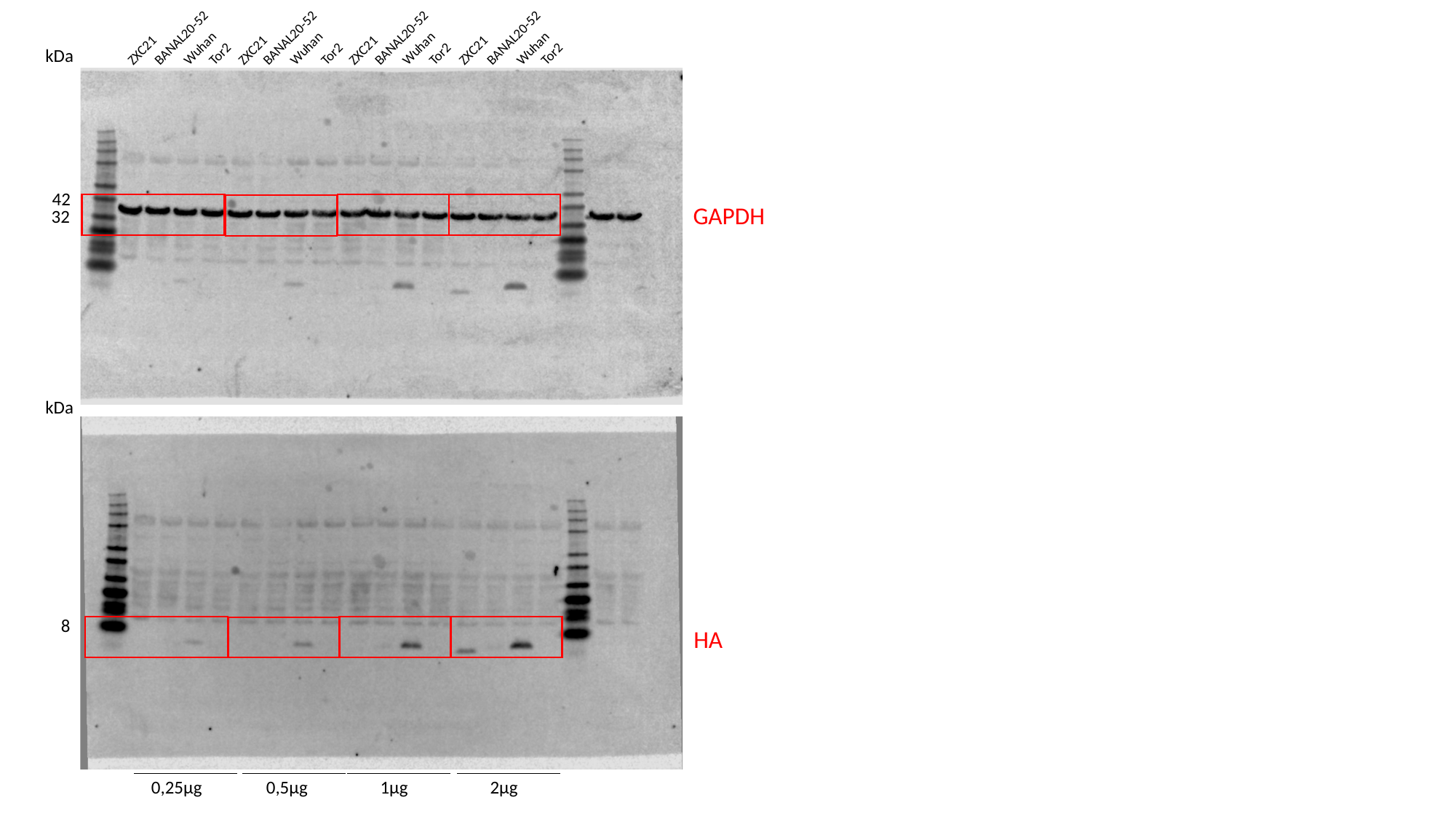

Wuhan
Wuhan
Wuhan
BANAL20-52
BANAL20-52
BANAL20-52
BANAL20-52
ZXC21
Tor2
Tor2
Wuhan
Tor2
ZXC21
ZXC21
Tor2
ZXC21
kDa
42
GAPDH
32
kDa
8
HA
2µg
0,25µg
0,5µg
1µg

## Slide 2
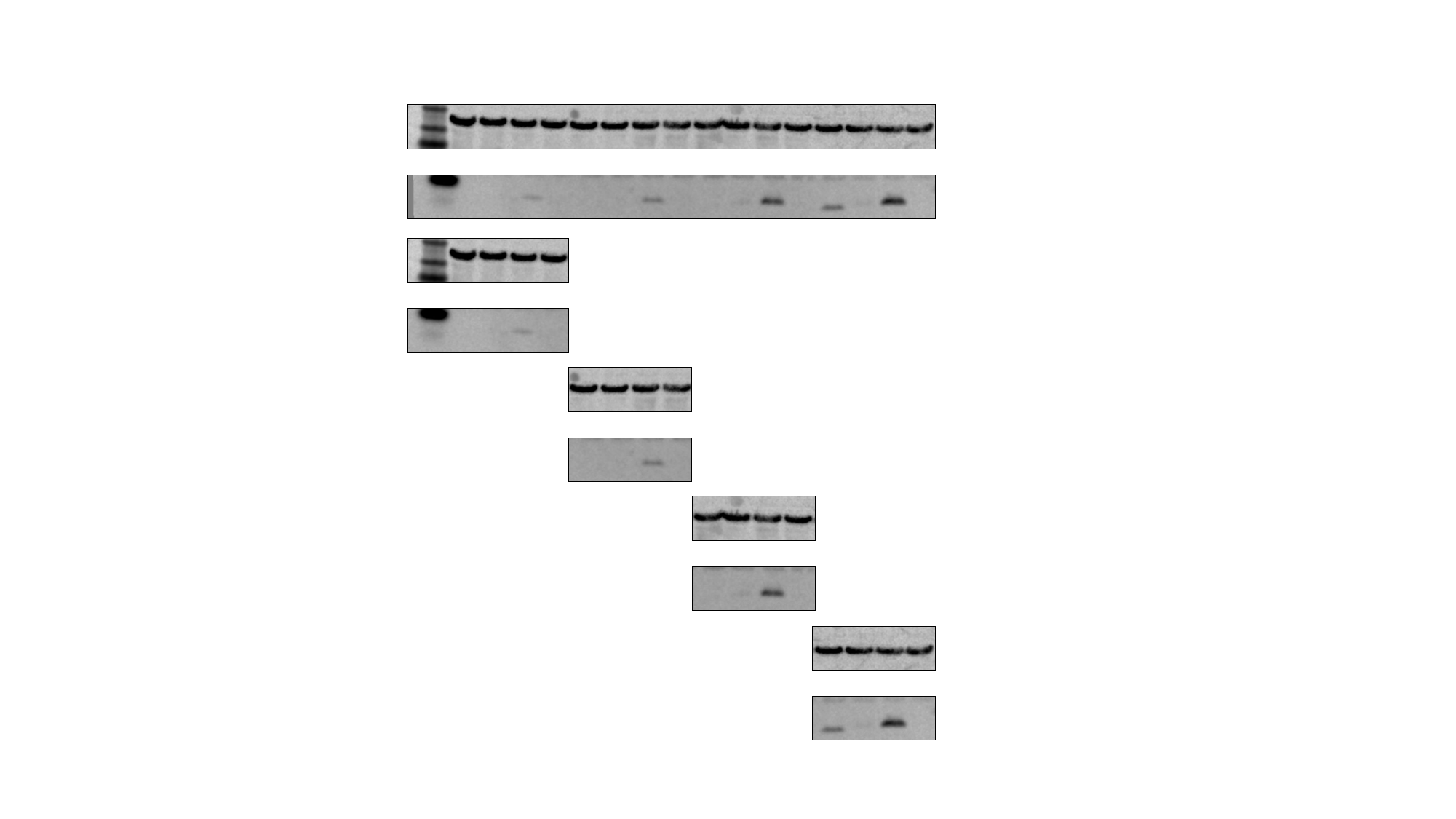

Supplement: Supplementary file 6 — Source Data for Figure 3 [file EMBR-24-e57137-s005.zip › Fig3C/EMBOR-2023-57137V2_SourceDataForFigure3C.pptx]
