# Supplementary material for: ORF3c is expressed in SARS‐CoV‐2‐infected cells and inhibits innate sensing by targeting MAVS
Source: EMBO Rep. 2023 Oct 23;24(12):e57137. doi: 10.15252/embr.202357137 (PMC10702836; doi:10.15252/embr.202357137)
Supplement: Supplementary file 9 [file EMBR-24-e57137-s004.pdf]

EMBO reports

ORF3c is expressed in SARS-CoV-2 infected cells and inhibits innate sensing by targeting MAVS

Martin Müller, Alexandra Herrmann, Shigeru Fujita, Keiya Uriu, Carolin Kruth, Adam Strange, Jan Kolberg, Markus Schneider, Jumpei Ito, Marcel Müller, Christian Drosten, Armin Ensser, Kei Sato, and Daniel Sauter
DOI: 10.15252/embr.202357137

Corresponding author(s): Daniel Sauter (daniel.sauter@med.uni-tuebingen.de)

Review Timeline:

Submission Date:	8th Mar 23
Editorial Decision:	31st Mar 23
Revision Received:	20th Sep 23
Editorial Decision:	29th Sep 23
Revision Received:	5th Oct 23
Accepted:	6th Oct 23

Editor: Achim Breiling

Transaction Report:

(Note: With the exception of the correction of typographical or spelling errors that could be a source of ambiguity, letters and reports are not edited. Depending on transfer agreements, referee reports obtained elsewhere may or may not be included in this compilation. Referee reports are anonymous unless the Referee chooses to sign their reports.)

Dear Prof. Sauter,

Thank you for the submission of your manuscript to EMBO reports. I have now received the reports from the three referees that were asked to evaluate your study, which can be found at the end of this message. Before sending out the study, I also obtained input from an advisor, which is also (partly) included, as I feel this is important.

As you will see, the referees indicate that these findings are of interest. However, they have several comments, concerns, and suggestions, indicating that a major revision of the manuscript is necessary to allow publication of the study in EMBO reports. As the reports are below, and all the referee concerns need to be addressed as indicated in the reports, I will not detail them here. However, I think it is important to show that ORF3c is expressed in virally infected cells (asked for by referee #2 and the advisor).

Given the constructive referee comments, I would like to invite you to revise your manuscript with the understanding that all referee concerns must be addressed in the revised manuscript and in a detailed point-by-point response. Acceptance of your manuscript will depend on a positive outcome of a second round of review. It is EMBO reports policy to allow a single round of revision only and acceptance of the manuscript will therefore depend on the completeness of your responses included in the next, final version of the manuscript.

Revised manuscripts should be submitted within three months of a request for revision. Please contact me to discuss the revision (also by video chat) if you have questions or comments regarding the revision, or should you need additional time.

When submitting your revised manuscript, please also carefully review the instructions that follow below.

PLEASE NOTE THAT upon resubmission revised manuscripts are subjected to an initial quality control prior to exposition to re-review. Upon failure in the initial quality control, the manuscripts are sent back to the authors, which may lead to delays. Frequent reasons for such a failure are the lack of the data availability section (please see below) and the presence of statistics based on n=2 (the authors are then asked to present scatter plots or provide more data points).

When submitting your revised manuscript, we will require:

1) a .docx formatted version of the final manuscript text (including legends for main figures, EV figures and tables), but without the figures included. Figure legends should be compiled at the end of the manuscript text.

2) individual production quality figure files as .eps, .tif, .jpg (one file per figure), of main figures (up to 8) and EV figures. Please upload these as separate, individual files upon re-submission.

The Expanded View format, which will be displayed in the main HTML of the paper in a collapsible format, has replaced the Supplementary information. You can submit up to 5 images as Expanded View. Please follow the nomenclature Figure EV1, Figure EV2 etc. The figure legend for these should be included in the main manuscript document file in a section called Expanded View Figure Legends after the main Figure Legends section. Additional Supplementary material should be supplied as a single pdf file labeled Appendix. The Appendix should have page numbers and needs to include a table of content on the first page (with page numbers) and legends for all content. Please follow the nomenclature Appendix Figure Sx, Appendix Table Sx etc. throughout the text, and also label the figures and tables according to this nomenclature.

For more details, please refer to our guide to authors:
http://www.embopress.org/page/journal/14693178/authorguide#manuscriptpreparation

Please consult our guide for figure preparation:
http://wol-prod-cdn.literatumonline.com/pb-assets/embo-site/EMBOPress_Figure_Guidelines_061115-1561436025777.pdf

See also the guidelines for figure legend preparation:
https://www.embopress.org/page/journal/14693178/authorguide#figureformat

3) a complete author checklist, which you can download from our author guidelines (https://www.embopress.org/page/journal/14693178/authorguide). Please insert page numbers in the checklist to indicate where the requested information can be found in the manuscript. The completed author checklist will also be part of the RPF.

Please also follow our guidelines for the use of living organisms, and the respective reporting guidelines:
http://www.embopress.org/page/journal/14693178/authorguide#livingorganisms

4) that primary datasets produced in this study (e.g. RNA-seq, ChIP-seq, structural and array data) are deposited in an appropriate public database. If no primary datasets have been deposited, please also state this in a dedicated section (e.g. 'No

primary datasets have been generated and deposited'), see below.

See also: http://embor.embopress.org/authorguide#datadeposition

Please remember to provide a reviewer password if the datasets are not yet public.

The accession numbers and database should be listed in a formal "Data Availability" section (placed after Materials & Methods) that follows the model below. This is now mandatory (like the COI statement). Please note that the Data Availability Section is restricted to new primary data that are part of this study. This section is mandatory. As indicated above, if no primary datasets have been deposited, please state this in this section

Data availability

The datasets produced in this study are available in the following databases:

- RNA-Seq data: Gene Expression Omnibus GSE46843 (https://www.ncbi.nlm.nih.gov/geo/query/acc.cgi?acc=GSE46843)
- [data type]: [name of the resource] [accession number/identifier/doi] ([URL or identifiers.org/DATABASE:ACCESSION])

*** Note - All links should resolve to a page where the data can be accessed. ***

Moreover, I have these editorial requests:

6) We now request the publication of original source data with the aim of making primary data more accessible and transparent to the reader. Our source data coordinator will contact you to discuss which figure panels we would need source data for and will also provide you with helpful tips on how to upload and organize the files.

7) Our journal encourages inclusion of *data citations in the reference list* to directly cite datasets that were re-used and obtained from public databases. Data citations in the article text are distinct from normal bibliographical citations and should directly link to the database records from which the data can be accessed. In the main text, data citations are formatted as follows: "Data ref: Smith et al, 2001" or "Data ref: NCBI Sequence Read Archive PRJNA342805, 2017". In the Reference list, data citations must be labeled with "[DATASET]". A data reference must provide the database name, accession number/identifiers and a resolvable link to the landing page from which the data can be accessed at the end of the reference. Further instructions are available at: http://www.embopress.org/page/journal/14693178/authorguide#referencesformat

8) Regarding data quantification and statistics, please make sure that the number "n" for how many independent experiments were performed, their nature (biological versus technical replicates), the bars and error bars (e.g. SEM, SD) and the test used to calculate p-values is indicated in the respective figure legends (also for potential EV figures and all those in the final Appendix). Please also check that all the p-values are explained in the legend, and that these fit to those shown in the figure. Please provide statistical testing where applicable. Please avoid the phrase 'independent experiment', but clearly state if these were biological or technical replicates. Please also indicate (e.g. with n.s.) if testing was performed, but the differences are not significant. In case n=2, please show the data as separate datapoints without error bars and statistics. See also: http://www.embopress.org/page/journal/14693178/authorguide#statisticalanalysis

If n<5, please show single datapoints for diagrams.

9) Please add scale bars of similar style and thickness to microscopic images, using clearly visible black or white bars (depending on the background). Please place these in the lower right corner of the images themselves. Please do not write on or near the bars in the image but define the size in the respective figure legend.

10) Please also note our reference format:
http://www.embopress.org/page/journal/14693178/authorguide#referencesformat

11) We updated our journal's competing interests policy in January 2022 and request authors to consider both actual and perceived competing interests. Please review the policy https://www.embopress.org/competing-interests and update your competing interests if necessary. Please name this section 'Disclosure and Competing Interests Statement' and put it after the Acknowledgements section.

12) We now use CRediT to specify the contributions of each author in the journal submission system. CRediT replaces the author contribution section. Please use the free text box to provide more detailed descriptions and remove the author contributions from the manuscript. See also guide to authors:
https://www.embopress.org/page/journal/14693178/authorguide#authorshipguidelines

Please order the manuscript sections like this, using these names:
Title page - Abstract - Keywords - Introduction - Results - Discussion - Materials and Methods - Data availability section -

Acknowledgements - Disclosure and Competing Interests Statement - References - Figure legends - Expanded View Figure legends

I look forward to seeing a revised version of your manuscript when it is ready. Please let me know if you have questions or comments regarding the revision.

Yours sincerely,

Achim Breiling
Senior Editor
EMBO Reports

Referee #1:

In the manuscript titled "SARS-CoV-2 ORF3c suppresses immune activation by inhibiting innate sensing" Mueller et al examine how ORF3c, a 41 aa peptide encoded from the ORF3a sgRNA, modulates cellular innate immune responses to infection. Specifically, they examine RLR antagonism to demonstrate that ORF3c inhibits both RIG-I and MDA-5 activation to dampen IFN production. Although the authors demonstrate that ORF3c can interact with MAVS, the relevance of this interaction is not fully addressed. In addition, they find that loss of ORF3c does not significantly affect SARS-CoV2 replication in cell culture. The data presented is novel and compelling. The study could be strengthened by the inclusion of a few key control experiments and mechanistic insights as suggested below:

1) Figure 2: To better understand the mechanism by which ORF3c suppresses IFN responses it would be useful for the authors to address the following: What is the relevance of the interaction between ORF3c and MAVS? Does ORF3c prevent RLR:MAVS interactions or MAVS oligomerization? Is MAVS stability compromised by ORF3c overexpression? Is there a decrease in the activation and nuclear translocation of endogenous IRF3 upon overexpression of ORF3c? Do the L2A/L3A and I6A/L7A mutants fail to interact with MAVS?

2) Figure 3: What dictates the capacity of distinct sarbecoviruses tested to inhibit IFN responses. Is the expression of ORF3c from Cov-2, ZXC21, Tor2 and BANAL-20-50 across the doses tested? Do peptides that display differential IFN antagonism interact with MAVS to the same extent?

3) Figure 4D: Does the localization of ORF3c change upon dsRNA virus challenge? Does it colocalize with mitochondria or other organelles relevant for MAVS activation (peroxisome)?

4) Figure 5: Although there were no observed changes in infectivity, are there changes in the induction of RLR-dependent IFN responses (PMID: 33514628) between viruses lacking ORF3c expression?

Referee #2:

This study explored a possible redundant protein ORF3c encoded by the SARS-CoV-2. The authors found that in cells ORF3c can be effectively expressed by plasmid transfection and show the activity of inhibition of IFN-β induction by suppression RIG-I- and MDA5-mediated immune activation and interacts with the signaling adaptor MAVS. By analysis and testing the sequences and function of ORF3c in other strains in sarbecoviruses they found the ORF3c is conserved and showed same function in immunosuppressive activity. The author constructed deletion mutation and point mutation strain of ORF3c viruses and found that that the ORF3c is not essential for efficient viral replication in vivo. The results presented in this study are relatively solid and could contribute to a deeper understanding of the redundant protein function of coronaviruses. A basic issue of concern is as follows.

Although the author has verified the immunosuppressive function of this small protein through transfection and expression in cell, and preliminarily revealed a possible pathway for regulating host interferon production. There is still a great deal of controversy over whether and to what extent these reading frames like ORF3c, ORF3d and etc, are expressed during the infection process. Given that this study has constructed ORF3c deletion virus strain, can the authors verify the expression and level of ORF3c at the level of cell infection in vitro, deepening the significance of this study.

Referee #3:

The authors show that a small protein, ORF3c, produced from an overlapping reading frame within the SARS-CoV-2 genome can inhibit IFNb promoter activity. This inhibition occurred when IFNb promoter activity was induced by RIG-I or MDA5 overexpression or by Sendai virus infection, but not when MAVS or IRF3 were overexpressed. They show that ORF3c co-immunoprecipitates with MAVS. Comparison of viruses with and without an intact ORF3c showed that ORF3c does not affect viral replication in human cells. While this is an interesting start to a manuscript, important readouts are missing, some data is misinterpreted, and there is a lack of both mechanism and significance/relevance for ORF3c provided in the manuscript.

1. The only readout of IFN is an IFNb promoter reporter. No actual measurements of IFNb gene transcription or secreted protein were provided. Particularly in viral infection experiments with SARS-CoV-2 where ORF3c was disrupted, it would have been interesting to know if IFNb production was affected.

2. Line 133-134: "...demonstrate that ORF3c suppresses IFN-β expression independently of the pattern recognition receptor, i.e. downstream of RIG-I and MDA5." I take issue with the phrase "independently of the pattern recognition receptor." The authors did not test any other pattern recognition receptors (TLRs, cGAS, etc) so they don't know if IFNb is inhibited independently of pattern recognition. Further, they do not demonstrate that inhibition occurs downstream of RIG-I or MDA5 since everything that they overexpressed downstream of RIG-I remained able to induce IFNb promoter activity in the presence of ORF3c.

3. The authors invoke/imply a mechanism whereby ORF3c inhibits IFN induction via interaction with MAVS. How do they explain that MAVS overexpression is still able to induce IFNb promoter activity in the presence of ORF3c? Overall, mechanistic insights into how ORF3c inhibits IFNb promoter activity is entirely lacking.

4. If ORF3c is not important for viral replication in human cells, might it be more important in an animal host? Might an effect be seen in bat cells or some other likely reservoir species? Alternatively, have the authors tested their rescued viruses in an animal model to determine if there are in vivo effects of ORF3c that are not readily apparent in cell lines?

Advisor:

A key open question to me is whether ORF3c is expressed in virally infected cells. All the experiments use overexpression. It is possible that ORF3c is not expressed in infected cells, or at much lower levels than in transfected cells. To address this, the authors would need to produce an anti-ORF3c antibody.

Another limitation is that the authors do not measure interferon induction in the infection experiments, and that these are limited to cells. A phenotype of the ORF3c deficient virus may well be apparent only in vivo.

Referee #1:

In the manuscript titled "SARS-CoV-2 ORF3c suppresses immune activation by inhibiting innate sensing" Mueller et al examine how ORF3c, a 41 aa peptide encoded from the ORF3a sgRNA, modulates cellular innate immune responses to infection. Specifically, they examine RLR antagonism to demonstrate that ORF3c inhibits both RIG-I and MDA-5 activation to dampen IFN production. Although the authors demonstrate that ORF3c can interact with MAVS, the relevance of this interaction is not fully addressed. In addition, they find that loss of ORF3c does not significantly affect SARS-CoV2 replication in cell culture. The data presented is novel and compelling. The study could be strengthened by the inclusion of a few key control experiments and mechanistic insights as suggested below:

1) Figure 2: To better understand the mechanism by which ORF3c suppresses IFN responses it would be useful for the authors to address the following: What is the relevance of the interaction between ORF3c and MAVS? Does ORF3c prevent RLR:MAVS interactions or MAVS oligomerization?

- Since ORF3c was shown to co-immunoprecipitate with MAVS (Figure 2D), it is indeed tempting to speculate that it may also interfere with RLR:MAVS or MAVS:MAVS interaction. To test this hypothesis, we took advantage of a previously described bimolecular fluorophore complementation assay by the García-Sastre lab (PMID: 27807226). We found that ORF3c significantly reduced the interaction of RIG-I with MAVS (new Fig. 2F) indicating that ORF3c competes with RIG-I and potentially other RLRs for MAVS binding.

Is MAVS stability compromised by ORF3c overexpression?

- MAVS is still readily detectable in cells over-expressing ORF3c (e.g., Fig. 2E). However, we observed the emergence of a ~9 kDa fragment of MAVS in the presence of increasing amounts of ORF3c (new Fig. 2G). The antibody used for detection is directed against the C-terminus of MAVS, suggesting that ORF3c induces the removal of the C-terminal transmembrane domain of MAVS. Since the transmembrane domain is essential for the signaling activity of MAVS (PMID: 16125763, 30715798), this proteolytic processing step may result in the inactivation of MAVS and contribute to the immunosuppressive activity of ORF3c.

Is there a decrease in the activation and nuclear translocation of endogenous IRF3 upon overexpression of ORF3c?

- Indeed, Western blot analysis of Sendai virus infected cells revealed decreased IRF3 phosphorylation (Ser 386) in the presence of ORF3c (new Fig. EV3A). Furthermore, nuclear translocation of IRF3 upon Sendai virus infection was reduced in ORF3c expressing cells (new Fig. EV3B). Notably, however, total IRF3 protein levels remained unchanged (new Fig. EV3C).

Do the L2A/L3A and I6A/L7A mutants fail to interact with MAVS?

- In contrast to wild type ORF3c, these two ORF3c mutants showed no detectable co-immunoprecipitation with MAVS (new Fig. EV4). This loss of interaction most likely explains the significant decrease in their ability to suppress IFN-β promoter activation (Fig. 3D).

2) Figure 3: What dictates the capacity of distinct sarbecoviruses tested to inhibit IFN responses. Is the expression of ORF3c from Cov-2, ZXC21, Tor2 and BANAL-20-50 across the doses tested?

- Western blot analyses revealed that ORF3c from Wuhan Hu-1 and ZXC21 are expressed at higher levels than their counterparts of Tor2 and BANAL-20-52 (updated Fig. 3C and new Fig. EV4). Thus, the reduced immunosuppressive activity of Tor2 and BANAL-20-52 ORF3c may simply be the result of

differences in protein levels and/or stability. We now discuss this point in the results section: “*However, ORF3c of SARS-CoV-2 Wuhan Hu-1 and a closely related bat coronavirus (ZXC21) suppressed IFN-β promoter activation more efficiently than ORF3c of SARS-CoV Tor2 and SARS-CoV-2 BANAL-20-50, most likely because of differences in overall ORF3c protein levels (Fig 3C).*“

Do peptides that display differential IFN antagonism interact with MAVS to the same extent?

- To address this question, we analyzed all four ORF3c orthologs for a potential co-immunoprecipitation with MAVS. While we could confirm the interaction of MAVS with SARS-CoV-2 ORF3c, we found no evidence for an interaction with ZXC21, Tor2 or BANAL-20-50 ORF3c (new Fig. EV4). However, the lack of ORF3c detection in the precipitate may simply be the result of lower input signals (see also previous point). We therefore refrain from drawing any conclusions regarding possible differences in ORF3c:MAVS interaction.

3) Figure 4D: Does the localization of ORF3c change upon dsRNA virus challenge? Does it colocalize with mitochondria or other organelles relevant for MAVS activation (peroxisome)?

- In agreement with a direct interaction with MAVS, SARS-CoV-2 ORF3c partially co-localizes with a mitochondrial marker (new Fig. EV2). This is in line with a recent study by Mozzi and colleagues that also observed a localization of ORF3c to mitochondria (PMID: 37361873). We found no evidence for a re-localization of ORF3c upon infection with Sendai virus (new Fig. EV2), although Sendai virus-induced nuclear translocation of IRF3 was inhibited by ORF3c (new Fig. EV3B).

4) Figure 5: Although there were no observed changes in infectivity, are there changes in the induction of RLR-dependent IFN responses (PMID: 33514628) between viruses lacking ORF3c expression?

- To address this point, we performed two complementary experiments: First, we quantified *IFNB1* mRNA levels in CaCo-2 and CaLu-3 cells infected with wild type or ORF3c-deficient SARS-CoV-2 (samples of the experiment shown in Fig. 5A). In CaCo-2 cells, *IFNB1* mRNA levels were generally undetectable or very low (Ct values >35). In Calu-3 cells, we observed an increase of *IFNB1* expression over time (new Fig. EV5A). However, there was no significant difference between wild type and ORF3c-deficient SARS-CoV-2. Second, we took advantage of a membrane-based cytokine array to monitor the release of 40 different cytokines (including IFN-β) from CaLu-3 cells infected with wild type or ORF3c-deficient SARS-CoV-2 (samples of the experiment shown in Fig. 5C). In agreement with the qRT-PCR data, we detected low amounts of IFN-β, but no marked difference between wild type or ORF3c-deficient SARS-CoV-2 (new Fig. EV5B). Together, these findings are in line with the observation that loss of ORF3c does not affect viral replication in infected CaCo-2 or CaLu-3 cells (Fig. 5).

Referee #2:

This study explored a possible redundant protein ORF3c encoded by the SARS-CoV-2. The authors found that in cells ORF3c can be effectively expressed by plasmid transfection and show the activity of inhibition of IFN-β induction by suppression RIG-I- and MDA5-mediated immune activation and interacts with the signaling adaptor MAVS. By analysis and testing the sequences and function of ORF3c in other strains in sarbecoviruses they found the ORF3c is conserved and showed same function in immunosuppressive activity. The author constructed deletion mutation and point mutation strain of ORF3c viruses and found that that the ORF3c is not essential for efficient viral replication in vivo. The results presented in this study are relatively solid and could contribute to a deeper understanding of the redundant protein function of coronaviruses. A basic issue of concern is as follows.

Although the author has verified the immunosuppressive function of this small protein through transfection and expression in cell, and preliminarily revealed a possible pathway for regulating host interferon production. There is still a great deal of controversy over whether and to what extent these reading frames like ORF3c, ORF3d and etc, are expressed during the infection process. Given that this study has constructed ORF3c deletion virus strain, can the authors verify the expression and level of ORF3c at the level of cell infection in vitro, deepening the significance of this study.

- Indeed, previous ribosomal profiling and immunopeptidome studies only provided indirect evidence for the presence of ORF3c in SARS-CoV-2 infected cells (PMID: 32906143, 37398281) Using circular polymerase extension reaction (CPER), we therefore generated a SARS-CoV-2 variant expressing HA-tagged ORF3c. New Fig. 1F demonstrates that ORF3c is readily detectable in SARS-CoV-2 infected CaCo-2 cells, providing first direct evidence for the expression of this viral protein.

Referee #3:

The authors show that a small protein, ORF3c, produced from an overlapping reading frame within the SARS-CoV-2 genome can inhibit IFNb promoter activity. This inhibition occurred when IFNb promoter activity was induced by RIG-I or MDA5 overexpression or by Sendai virus infection, but not when MAVS or IRF3 were overexpressed. They show that ORF3c co-immunoprecipitates with MAVS. Comparison of viruses with and without an intact ORF3c showed that ORF3c does not affect viral replication in human cells. While this is an interesting start to a manuscript, important readouts are missing, some data is misinterpreted, and there is a lack of both mechanism and significance/relevance for ORF3c provided in the manuscript.

1. The only readout of IFN is an IFNb promoter reporter. No actual measurements of IFNb gene transcription or secreted protein were provided. Particularly in viral infection experiments with SARS-CoV-2 where ORF3c was disrupted, it would have been interesting to know if IFNb production was affected.

- To address this point, we performed two complementary experiments: First, we quantified *IFNB1* mRNA levels in CaCo-2 and CaLu-3 cells infected with wild type or ORF3c-deficient SARS-CoV-2 (samples of the experiment shown in Fig. 5A). In CaCo-2 cells, *IFNB1* mRNA levels were generally undetectable or very low (Ct values >35). In Calu-3 cells, we observed an increase of *IFNB1* expression over time (new Fig. EV5A). However, there was no significant difference between wild type and ORF3c-deficient SARS-CoV-2. Second, we took advantage of a membrane-based cytokine array to monitor the release of 40 different cytokines (including IFN-β) from CaLu-3 cells infected with wild type or ORF3c-deficient SARS-CoV-2 (samples of the experiment shown in Fig. 5C). In agreement with the qRT-PCR data, we detected low amounts of IFN-β, but no marked difference between wild type or ORF3c-deficient SARS-CoV-2 (new Fig. EV5B). Together, these findings are in line with the observation that loss of ORF3c does not affect viral replication in infected CaCo-2 or CaLu-3 cells (Fig. 5).

2. Line 133-134: "...demonstrate that ORF3c suppresses IFN-β expression independently of the pattern recognition receptor, i.e. downstream of RIG-I and MDA5." I take issue with the phrase "independently of the pattern recognition receptor." The authors did not test any other pattern recognition receptors (TLRs, cGAS, etc) so they don't know if IFNb is inhibited independently of pattern recognition. Further, they do not demonstrate that inhibition occurs downstream of RIG-I or MDA5 since everything that they overexpressed downstream of RIG-I remained able to induce IFNb promoter activity in the presence of ORF3c.

- We agree that this statement may have been misleading and removed it from the manuscript. More importantly, we now provide several lines of evidence demonstrating that ORF3c targets the signaling molecule MAVS:
(1) ORF3c co-immunoprecipitates with MAVS (Fig. 2D).
(2) ORF3c inhibits the interaction of RIG-I with MAVS (new Fig. 2F).
(3) ORF3c induces C-terminal proteolytic processing of MAVS (new Fig. 2G).

The observation that ORF3c fails to inhibit IFN-β induction upon expression of constitutively active IRF3 (Fig. 2C) suggests that ORF3c inhibits a factor upstream of this transcription factor and is in line with a targeting of MAVS.

3. The authors invoke/imply a mechanism whereby ORF3c inhibits IFN induction via interaction with MAVS. How do they explain that MAVS overexpression is still able to induce IFNb promoter activity in the presence of ORF3c?

- As mentioned in our reply to comment #2, we now demonstrate that ORF3c inhibits the interaction of RIG-I with MAVS. This suggests that ORF3c and RIG-I compete with MAVS binding. We therefore hypothesize that over-expression of MAVS can saturate the inhibitory effect of ORF3c and compensate for the binding and inhibition of endogenous MAVS. We have added a brief section in the discussion to clarify this point: "*A competitive binding of ORF3c and RIG-I to MAVS may also explain why ORF3c fails to suppress IFN-β promoter activation when MAVS over-expression is used as an inducer (Fig. 2C). We hypothesize that exogenous MAVS may saturate the inhibitory activity of ORF3c and compensate for endogenous MAVS that is bound and blocked by ORF3c.*"

Overall, mechanistic insights into how ORF3c inhibits IFNb promoter activity is entirely lacking.

- We have performed several additional experiments providing important insights into the mechanisms underlying the immuno-suppressive activity of ORF3c. Among other things, we now show that
(1) ORF3c inhibits the interaction of RIG-I with MAVS (new Fig. 2F).
(2) ORF3c induces C-terminal proteolytic processing of MAVS (new Fig. 2G).
(3) ORF3c localization is not altered upon infection (new Fig. EV2).
(4) ORF3c reduces the phosphorylation of IRF3 at Ser 386 upon Sendai virus infection (new Fig. EV3A).
(5) ORF3c reduces nuclear translocation of IRF3 upon Sendai virus infection (new Fig. EV3B).
(5) the ORF3c mutants L2A/L3A and I6A/L7A lost their ability to interact with MAVS (new Fig. EV4).

All these experiments are in line with MAVS being the target of ORF3c.

4. If ORF3c is not important for viral replication in human cells, might it be more important in an animal host? Might an effect be seen in bat cells or some other likely reservoir species? Alternatively, have the authors tested their rescued viruses in an animal model to determine if there are in vivo effects of ORF3c that are not readily apparent in cell lines?

- We agree that effects of ORF3c on viral replication and spread may only be apparent *in vivo* or in a natural animal host. To identify a potential selection advantage of ORF3c *in vivo*, we have therefore analyzed the emergence of ORF3c-deficient SARS-CoV-2 variants and potential revertants in humans rather than in a non-natural host such as mice. We found that a substantial fraction of B.1.617 and B.1.630 lineages harbors premature stop codons in ORF3c (Fig. 4G). Intriguingly, however, a subset of B.1.617.2 viruses acquired an additional point mutation at a later time point, reverting the stop codon at position 5 to a tyrosine (*5Y), thereby reconstituting *ORF3c*. Together, these findings

demonstrate that ORF3c is dispensable for efficient viral replication *in vivo*, but may confer a selection advantage to the virus.

To investigate a potential effect of ORF3c in an animal host, we monitored replication of wild type and ORF3c-deficient SARS-CoV-2 in an immortalized lung cell line from *Rhinolophus alcyone* that stably expresses human ACE2 (PMID: 34824253). Although *R. alcyone* does not represent the true animal reservoir of SARS-CoV-2, closely related betacoronaviruses have been found in other members of the *Rhinolophus* genus. As shown in new Fig. 5D, we found no evidence for potential species-specific effects of ORF3c, since wild type and ORF3c-deficient SARS-CoV-2 replicated with similar kinetics in *Rhinolophus alcyone* cells.

Advisor:

A key open question to me is whether ORF3c is expressed in virally infected cells. All the experiments use overexpression. It is possible that ORF3c is not expressed in infected cells, or at much lower levels than in transfected cells. To address this, the authors would need to produce an anti-ORF3c antibody.

- To address this question, we initially immunized chicken with an ORF3c-derived peptide (amino acids 2-20: LQRYRYKPHSLSDGLLLALHF). Unfortunately, however, we were not able to raise any antiserum or purified IgG/IgY against ORF3c (not shown). In an alternative approach, we therefore tagged ORF3c in the viral genome and rescued infectious SARS-CoV-2 encoding HA-tagged ORF3c. Importantly, Western blotting demonstrated that ORF3c is readily detectable in SARS-CoV-2 infected cells (new Fig. 1F, see also reply to reviewer #2), while it was sometimes close to the detection limit in transfected cells (see e.g. Fig. 1F and titration in Fig. 3C).

Another limitation is that the authors do not measure interferon induction in the infection experiments, and that these are limited to cells. A phenotype of the ORF3c deficient virus may well be apparent only in vivo.

- To address the first point, we have used the samples of the infection experiments shown in Fig. 5A and quantified *IFNB1* mRNA levels in CaCo-2 and CaLu-3 cells infected with wild type or ORF3c-deficient SARS-CoV-2. In CaCo-2 cells, *IFNB1* mRNA levels were generally undetectable or very low (Ct values >35). In Calu-3 cells, we observed an increase of *IFNB1* expression over time. However, there was no significant difference between wild type and ORF3c-deficient SARS-CoV-2 (new Fig. EV5A). Furthermore, a cytokine array of the samples shown in Fig. 5C showed no evidence for altered IFN-β protein levels in the presence or absence of ORF3c (new Fig. EV5B). These findings are in line with the observation that loss of ORF3c does not affect viral replication in this experimental setup (Fig. 5A, C).
We also agree that a phenotype of ORF3c may only be apparent in vivo and/or in an animal host of SARS-CoV-2. Unfortunately, already approved experiments in mice had to be cancelled since the BSL3 animal facility of our collaboration partners had to be closed down due to contaminations. We therefore followed two alternative approaches: First, we monitored the emergence of natural ORF3c loss events in the human population (Fig. 4G) and potential revertants thereof. These findings and possible implications for the role of ORF3c *in vivo* are now discussed in more detail. Second, we monitored a potential effect of ORF3c on viral replication in a lung cell line from *Rhinolophus* bats to exclude that a phenotype is only apparent in bat, but not human cells. However, we found no evidence for a species-specific effect of ORF3c in bats vs. humans since wild type and ORF3c-deficient viruses also replicated with similar efficacies in bat cells (new Fig. 5D).

Dear Prof. Sauter,

Thank you for the submission of your revised manuscript to our editorial offices. I have now received the reports from the three referees that have been asked to re-evaluate your paper, you will find below. As you will see, referees #2 and #3 now fully support the publication of the study in EMBO reports. Referee #1 has some remaining concerns and suggestions to improve the study, I ask you to address in a final revised manuscript. We will not require to address the immune evasion roles of ORF3c in animal models, though. Nevertheless address the points by referee #1 in a final p-b-p-response, provide the data for point 1 (if feasible) and provide a detailed discussion on point 2 in the manuscript text.

Moreover, I have these editorial requests:

- Please provide the abstract written in present tense throughout.

- We now use CRediT to specify the contributions of each author in the journal submission system. CRediT replaces the author contribution section. Please use the free text box to provide more detailed descriptions and do NOT provide your final manuscript text file with an author contributions section. See also our guide to authors: https://www.embopress.org/page/journal/14693178/authorguide#authorshipguidelines

- Please remove the section 'RESOURCE AVAILABILITY' from the manuscript. Resources, material sand reagents of EMBO press papers are always available from the corresponding author(s).

- Please add scale bars of similar style and thickness to the microscopic images, using clearly visible black or white bars (depending on the background). Please place these in the lower right corner of the images themselves. Please do not write on or near the bars in the image but define the size in the respective figure legend. Presently, the scale bars are either hardly visible (4D) or absent (EV2, EV3).

- The images in 5C and D below the diagrams are very small and it is hard to see what they show. Could these be displayed differently/larger (maybe as part of one of the EV figures)? We would also need scale bars for these images.

- Regarding data quantification and statistics, please make sure that the number "n" for how many independent experiments were performed, their nature (biological versus technical replicates), the bars and error bars (e.g. SEM, SD) and the test used to calculate p-values is indicated in the respective figure legends (main and EV figures). Please also check that all the p-values are explained in the legend, and that these fit to those shown in the figure. Please provide statistical testing where applicable. Please avoid the phrase 'independent experiment', but clearly state if these were biological or technical replicates. Please also indicate (e.g. with n.s.) if testing was performed, but the differences are not significant. In case n=2 please show the data as separate datapoints or bars without error bars and statistics. See also: http://www.embopress.org/page/journal/14693178/authorguide#statisticalanalysis

If n<5, please show single datapoints for diagrams.

Please note that the error bars are not defined in the legend of figure EV1b.

Please indicate what the red arrows represent in the legends of figures 2d-e; EV4.

- Please note that legend for figure 2F should start on a new line OR it should be labeled as D, E, F.

- Please use our reference format: http://www.embopress.org/page/journal/14693178/authorguide#referencesformat

- Thanks for providing the source data. Please upload the SD for EV figures as one ZIP folder.

- In Figure 4D, ORF3c Green Mock-Cell has no image noise/background. This is basically a blank image (also in the SD). Wouldn't some background be expected? Please check and explain.

In addition, I would need from you:
- a short, two-sentence summary of the manuscript (not more than 35 words).
- two to four short (!) bullet points highlighting the key findings of your study (two lines each).
- a schematic summary figure as separate file that provides a sketch of the major findings (not a data image) in jpeg or tiff format (with the exact width of 550 pixels and a height of not more than 400 pixels) that can be used as a visual synopsis on our website.

I look forward to seeing the final revised version of your manuscript when it is ready. Please let me know if you have questions

regarding the revision.

Best,

Achim Breiling
Senior Editor
EMBO Reports

Referee #1:

1. In the revised manuscript the authors examine the expression of various sarbecovirus ORF3C and show decreased MAVS interactions. While the lower level of expression is appreciable, this result confounds the model suggesting that ORF3C inhibition of RIG-I:MAVS interactions leads to impaired IFN production and the conservation of these phenotypes across sarbecoviruses. Particularly given that the level of ZXC21 inhibition is greater than that achieved by Hu-1 ORF3C which expresses far more robustly. To enhance confidence, theses interaction could be studied using the bimolecular fluorescence complementation (BiFC) assay.
2. The functional consequences of ORF3C still remain to be addressed. The lack of changes in innate immune activation in bat, CaCo-2, and CaLu-3 cells strongly argues against a functional role for ORF3c in the inhibition of IFN responses during infection and/or that the use of these cell models is not appropriate to address this question. As suggested by other reviewers, it is critical to address the immune evasion roles of ORF3c in animal models.

Referee #2:

The author provided a good revision of the relevant concerns in the revised manuscript version, and there are no further questions.

Referee #3:

The authors have satisfactorily addressed my previous critiques. The issue remains that the significance/biological relevance of the newly discovered functions of orf3c remain to be shown in a viral infection. However, I do believe that this work is a valuable contribution to the field that will spark interest, debate, and additional research on orf3c.

Referee #1:

1. In the revised manuscript the authors examine the expression of various sarbecovirus ORF3C and show decreased MAVS interactions. While the lower level of expression is appreciable, this result confounds the model suggesting that ORF3C inhibition of RIG-I:MAVS interactions leads to impaired IFN production and the conservation of these phenotypes across sarbecoviruses. Particularly given that the level of ZXC21 inhibition is greater than that achieved by Hu-1 ORF3C which expresses far more robustly. To enhance confidence, theses interaction could be studied using the bimolecular fluorescence complementation (BiFC) assay.

The differences in ORF3c detection by Western blotting prevent a meaningful direct comparison of different ORF3c orthologs in the MAVS interaction assay (Fig EV4A). However, lack of (efficient) detection by Western blotting does not necessarily imply that the respective ORF3c proteins do not interact with MAVS.
To further address this point, we therefore also monitored the proteins levels of all four ORF3c orthologs by flow cytometry. Wuhan Hu-1, BANAL-20-50 and ZXC21 ORF3c (but not Tor2 ORF3c) were readily detectable via their HA-tag (Fig EV4B), which enabled us to monitor their effect on RIG-I:MAVS interaction using the BiFC assay. As shown in new Fig EV4C, all three ORF3c orthologs reduce the interaction of RIG-I with MAVS to a similar extent, demonstrating that this phenotype is not unique to ORF3c of Wuhan Hu-1.
We also agree that ORF3c of ZXC21 may suppress IFN-β induction slightly more efficiently than its Wuhan Hu-1 counterpart, although the latter is detected more efficiently by Western blotting (Fig 3C, EV4B). This suggests that ORF3c orthologs may not only differ in their expression (or detectability), but also in their relative immunosuppressive activity.

2. The functional consequences of ORF3C still remain to be addressed. The lack of changes in innate immune activation in bat, CaCo-2, and CaLu-3 cells strongly argues against a functional role for ORF3c in the inhibition of IFN responses during infection and/or that the use of these cell models is not appropriate to address this question. As suggested by other reviewers, it is critical to address the immune evasion roles of ORF3c in animal models.

We fully agree that an effect of ORF3c on viral replication may only be apparent *in vivo*. We have therefore expanded our discussion to further emphasize the importance of *in vivo* infections models. Experiments in small animals will help to fully understand the relative contribution of ORF3c to SARS-CoV-2-mediated immune evasion and replicative fitness. Due to potential species-specific effects, we also encourage future analyses in naturally infected humans to identify a potential association of (inactivating) mutations in *ORF3c* with viral loads or disease.

Referee #2:

The author provided a good revision of the relevant concerns in the revised manuscript version, and there are no further questions.

We thank this referee for their helpful comments. The proposed detection of ORF3c in infected cells clearly strengthened the manuscript.

Referee #3:

The authors have satisfactorily addressed my previous critiques. The issue remains that the significance/biological relevance of the newly discovered functions of orf3c remain to be shown in

a viral infection. However, I do believe that this work is a valuable contribution to the field that will spark interest, debate, and additional research on orf3c.

We agree and also encourage future experiments *in vivo* to define the role of ORF3c in SARS-CoV-2 replication and immune evasion. As noted in our response to point 2 of referee #1, we have expanded the discussion in our manuscript accordingly.

2nd Revision - Editorial Decision **6th Oct 2023**

Prof. Daniel Sauter
University of Tubingen
Tubingen
Germany

Dear Prof. Sauter,

I am very pleased to accept your manuscript for publication in the next available issue of EMBO reports. Thank you for your contribution to our journal.

At the end of this email I include important information about how to proceed. Please ensure that you take the time to read the information and complete and return the necessary forms to allow us to publish your manuscript as quickly as possible.

As part of the EMBO publication's Transparent Editorial Process, EMBO reports publishes online a Review Process File to accompany accepted manuscripts. As you are aware, this File will be published in conjunction with your paper and will include the referee reports, your point-by-point response and all pertinent correspondence relating to the manuscript.

If you do NOT want this File to be published, please inform the editorial office within 2 days, if you have not done so already, otherwise the File will be published by default [contact: emboreports@embo.org]. If you do opt out, the Review Process File link will point to the following statement: "No Review Process File is available with this article, as the authors have chosen not to make the review process public in this case."

Thank you again for your contribution to EMBO reports and congratulations on a successful publication. Please consider us again in the future for your most exciting work.

Yours sincerely,

Achim Breiling
Editor
EMBO Reports

****

THINGS TO DO NOW:

Please note that you will be contacted by Wiley Author Services to complete licensing and payment information. The required 'Page Charges Authorization Form' is available here: https://www.embopress.org/pb-assets/embo-site/er_apc.pdf - please download and complete the form and return to embopressproduction@wiley.com

EMBO Press participates in many Publish and Read agreements that allow authors to publish Open Access with reduced/no publication charges. Check your eligibility: https://authorservices.wiley.com/author-resources/Journal-Authors/open-access/affiliation-policies-payments/index.html

You will receive proofs by e-mail approximately 2-3 weeks after all relevant files have been sent to our Production Office; you should return your corrections within 2 days of receiving the proofs.

Please inform us if there is likely to be any difficulty in reaching you at the above address at that time. Failure to meet our deadlines may result in a delay of publication, or publication without your corrections.

All further communications concerning your paper should quote reference number EMBOR-2023-57137V3 and be addressed to emboreports@wiley.com.

Should you be planning a Press Release on your article, please get in contact with emboreports@wiley.com as early as possible, in order to coordinate publication and release dates.

EMBO Press Author Checklist

Corresponding Author Name: Daniel SAUTER	
Journal Submitted to: EMBO Reports	
Manuscript Number: EMBOR-2023-57137V1	

USEFUL LINKS FOR COMPLETING THIS FORM
The EMBO Journal - Author Guidelines
EMBO Reports - Author Guidelines
Molecular Systems Biology - Author Guidelines
EMBO Molecular Medicine - Author Guidelines

Reporting Checklist for Life Science Articles (updated January
This checklist is adapted from Materials Design Analysis Reporting (MDAR) Checklist for Authors. MDAR establishes a minimum set of requirements in transparent reporting in the life sciences (see Statement of Task: 10.31222/osf.io/9sm4x). Please follow the journal's guidelines in preparing your
Please note that a copy of this checklist will be published alongside your article.

Abridged guidelines for figures
1. Data
The data shown in figures should satisfy the following conditions:
- the data were obtained and processed according to the field's best practice and are presented to reflect the results of the experiments in an accurate and unbiased manner.
- ideally, figure panels should include only measurements that are directly comparable to each other and obtained with the same assay.
- plots include clearly labeled error bars for independent experiments and sample sizes. Unless justified, error bars should not be shown for technical
- if n<5, the individual data points from each experiment should be plotted. Any statistical test employed should be justified.
- Source Data should be included to report the data underlying figures according to the guidelines set out in the authorship guidelines on Data

2. Captions
Each figure caption should contain the following information, for each panel where they are relevant:
- a specification of the experimental system investigated (eg cell line, species name).
- the assay(s) and method(s) used to carry out the reported observations and measurements.
- an explicit mention of the biological and chemical entity(ies) that are being measured.
- an explicit mention of the biological and chemical entity(ies) that are altered/varied/perturbed in a controlled manner.
- the exact sample size (n) for each experimental group/condition, given as a number, not a range;
- a description of the sample collection allowing the reader to understand whether the samples represent technical or biological replicates (including how many animals, litters, cultures, etc.).
- a statement of how many times the experiment shown was independently replicated in the laboratory.
- definitions of statistical methods and measures:
  - common tests, such as t-test (please specify whether paired vs. unpaired), simple χ2 tests, Wilcoxon and Mann-Whitney tests, can be unambiguously identified by name only, but more complex techniques should be described in the methods section;
  - are tests one-sided or two-sided?
  - are there adjustments for multiple comparisons?
  - exact statistical test results, e.g., P values = x but not P values < x;
  - definition of 'center values' as median or average;
  - definition of error bars as s.d. or s.e.m.

Please complete ALL of the questions below.
Select "Not Applicable" only when the requested information is not relevant for your study.

Materials

Newly Created Materials	Information included in the manuscript?	In which section is the information available? (Reagents and Tools Table, Materials and Methods, Figures, Data Availability Section)
New materials and reagents need to be available; do any restrictions apply?	Yes	Resource Availability Section

Antibodies	Information included in the manuscript?	In which section is the information available? (Reagents and Tools Table, Materials and Methods, Figures, Data Availability Section)
For **antibodies** provide the following information:<br>- Commercial antibodies: RRID (if possible) or supplier name, catalogue number and or/clone number<br>- Non-commercial: RRID or citation	Yes	Materials and Methods

DNA and RNA sequences	Information included in the manuscript?	In which section is the information available? (Reagents and Tools Table, Materials and Methods, Figures, Data Availability Section)
**Short novel DNA or RNA including primers, probes**: provide the sequences.	Yes	Materials and Methods

Cell materials	Information included in the manuscript?	In which section is the information available? (Reagents and Tools Table, Materials and Methods, Figures, Data Availability Section)
**Cell lines:** Provide species information, strain. Provide accession number in repository **OR** supplier name, catalog number, clone number, and/**OR** RRID.	Yes	Materials and Methods
**Primary cultures:** Provide species, strain, sex of origin, genetic modification status.	Not Applicable	
Report if the cell lines were recently **authenticated** (e.g., by STR profiling) and tested for mycoplasma contamination.	Yes	Materials and Methods

Experimental animals	Information included in the manuscript?	In which section is the information available? (Reagents and Tools Table, Materials and Methods, Figures, Data Availability Section)
**Laboratory animals or Model organisms:** Provide species, strain, sex, age, genetic modification status. Provide accession number in repository **OR** supplier name, catalog number, clone number, **OR** RRID.	Not Applicable	
**Animal observed in or captured from the field:** Provide species, sex, and age where possible.	Not Applicable	
Please detail **housing and husbandry conditions**.	Not Applicable	

Plants and microbes	Information included in the manuscript?	In which section is the information available? (Reagents and Tools Table, Materials and Methods, Figures, Data Availability Section)
**Plants:** provide species and strain, ecotype and cultivar where relevant, unique accession number if available, and source (including location for collected wild specimens).	Not Applicable	
**Microbes:** provide species and strain, unique accession number if available, and source.	Yes	Materials and Methods

Human research participants	Information included in the manuscript?	In which section is the information available? (Reagents and Tools Table, Materials and Methods, Figures, Data Availability Section)
If collected and within the bounds of privacy constraints report on age, sex and gender or ethnicity for all study participants.	Not Applicable	

Core facilities	Information included in the manuscript?	In which section is the information available? (Reagents and Tools Table, Materials and Methods, Figures, Data Availability Section)
If your work benefited from core facilities, was their service mentioned in the acknowledgments section?	Not Applicable	

Design

Study protocol	Information included in the manuscript?	In which section is the information available? (Reagents and Tools Table, Materials and Methods, Figures, Data Availability Section)

If study protocol has been **pre-registered, provide DOI in the manuscript**. For clinical trials, provide the trial registration number **OR** cite DOI.	Not Applicable	
Report the **clinical trial registration number** (at ClinicalTrials.gov or equivalent), where applicable.	Not Applicable	

Laboratory protocol	Information included in the manuscript?	In which section is the information available? (Reagents and Tools Table, Materials and Methods, Figures, Data Availability Section)
Provide DOI OR other citation details if **external detailed step-by-step protocols** are available.	Not Applicable	

Experimental study design and statistics	Information included in the manuscript?	In which section is the information available? (Reagents and Tools Table, Materials and Methods, Figures, Data Availability Section)
Include a statement about **sample size** estimate even if no statistical methods were used.	Not Applicable	
Were any steps taken to minimize the effects of subjective bias when allocating animals/samples to treatment (e.g. **randomization procedure**)? If yes, have they been described?	Not Applicable	
Include a statement about **blinding** even if no blinding was done.	Yes	Materials and Methods
Describe **inclusion/exclusion criteria** if samples or animals were excluded from the analysis. Were the criteria pre-established?<br><br>If sample or data points were omitted from analysis, report if this was due to attrition or intentional exclusion and provide justification.	Not Applicable	
For every figure, are **statistical tests** justified as appropriate? Do the data meet the assumptions of the tests (e.g., normal distribution)? Describe any methods used to assess it. Is there an estimate of variation within each group of data? Is the variance similar between the groups that are being statistically compared?	Yes	Materials and Methods

Sample definition and in-laboratory replication	Information included in the manuscript?	In which section is the information available? (Reagents and Tools Table, Materials and Methods, Figures, Data Availability Section)
In the figure legends: state number of times the experiment was **replicated** in laboratory.	Yes	Figure legends
In the figure legends: define whether data describe **technical or biological replicates**.	Yes	Figure legends

Ethics

Ethics	Information included in the manuscript?	In which section is the information available? (Reagents and Tools Table, Materials and Methods, Figures, Data Availability Section)
Studies involving **human participants**: State details of **authority granting ethics approval** (IRB or equivalent committee(s), provide reference number for approval.	Not Applicable	
Studies involving **human participants**: Include a statement confirming that **informed consent** was obtained from all subjects and that the experiments conformed to the principles set out in the WMA Declaration of Helsinki and the Department of Health and Human Services Belmont Report.	Not Applicable	
Studies involving **human participants:** For publication of **patient photos**, include a statement confirming that consent to publish was obtained.	Not Applicable	
Studies involving experimental **animals**: State details of **authority granting ethics approval** (IRB or equivalent committee(s), provide reference number for approval. Include a statement of compliance with ethical regulations.	Not Applicable	
Studies involving **specimen and field samples:** State if relevant **permits** obtained, provide details of authority approving study; if none were required, explain why.	Not Applicable	

Dual Use Research of Concern (DURC)	Information included in the manuscript?	In which section is the information available? (Reagents and Tools Table, Materials and Methods, Figures, Data Availability Section)
Could your study fall under dual use research restrictions? Please check biosecurity documents and list of **select agents and toxins** (CDC): https://www.selectagents.gov/sat/list.htm	Not Applicable	
If you used a select agent, is the security level of the lab appropriate and reported in the manuscript?	Not Applicable	
If a study is subject to dual use research of concern regulations, is the name of the **authority granting approval and reference number** for the regulatory approval provided in the manuscript?	Not Applicable	

Reporting

The MDAR framework recommends adoption of discipline-specific guidelines, established and endorsed through community initiatives. Journals have their own policy about requiring specific guidelines and recommendations to complement MDAR.

Adherence to community standards	Information included in the manuscript?	In which section is the information available? (Reagents and Tools Table, Materials and Methods, Figures, Data Availability Section)
State if relevant guidelines or checklists (e.g., **ICMJE, MIBBI, ARRIVE, PRISMA**) have been followed or provided.	Not Applicable	
For **tumor marker prognostic studies,** we recommend that you follow the **REMARK** reporting guidelines (see link list at top right). See author guidelines, under 'Reporting Guidelines'. Please confirm you have followed these guidelines.	Not Applicable	
For **phase II and III randomized controlled trials**, please refer to the **CONSORT** flow diagram (see link list at top right) and submit the CONSORT checklist (see link list at top right) with your submission. See author guidelines, under 'Reporting Guidelines'. Please confirm you have submitted this list.	Not Applicable	

Data Availability

Data availability	Information included in the manuscript?	In which section is the information available? (Reagents and Tools Table, Materials and Methods, Figures, Data Availability Section)
Have **primary datasets** been deposited according to the journal's guidelines (see 'Data Deposition' section) and the respective accession numbers provided in the Data Availability Section?	Not Applicable	
Were **human clinical and genomic datasets** deposited in a public access-controlled repository in accordance to ethical obligations to the patients and to the applicable consent agreement?	Not Applicable	
Are **computational models** that are central and integral to a study available without restrictions in a machine-readable form? Were the relevant accession numbers or links provided?	Not Applicable	
If publicly available data were reused, provide the respective **data citations in the reference list**.	Yes	GISAID SARS-CoV-2 sequence repository: Materials and Methods, Results, Reference list
