# Supplementary material for: ORF3c is expressed in SARS‐CoV‐2‐infected cells and inhibits innate sensing by targeting MAVS
Source: EMBO Rep. 2023 Oct 23;24(12):e57137. doi: 10.15252/embr.202357137 (PMC10702836; doi:10.15252/embr.202357137)
Supplement: Supplementary file 3 — Source Data for Expanded View [file EMBR-24-e57137-s002.zip › EMBOR-2023-57137V3_SourceDataForExpandedView/EMBOR-2023-57137V3_SourceDataForExpandedView3.zip!/EV3A/EMBOR-2023-57137V2_SourceDataForExpandedView3A.pdf]

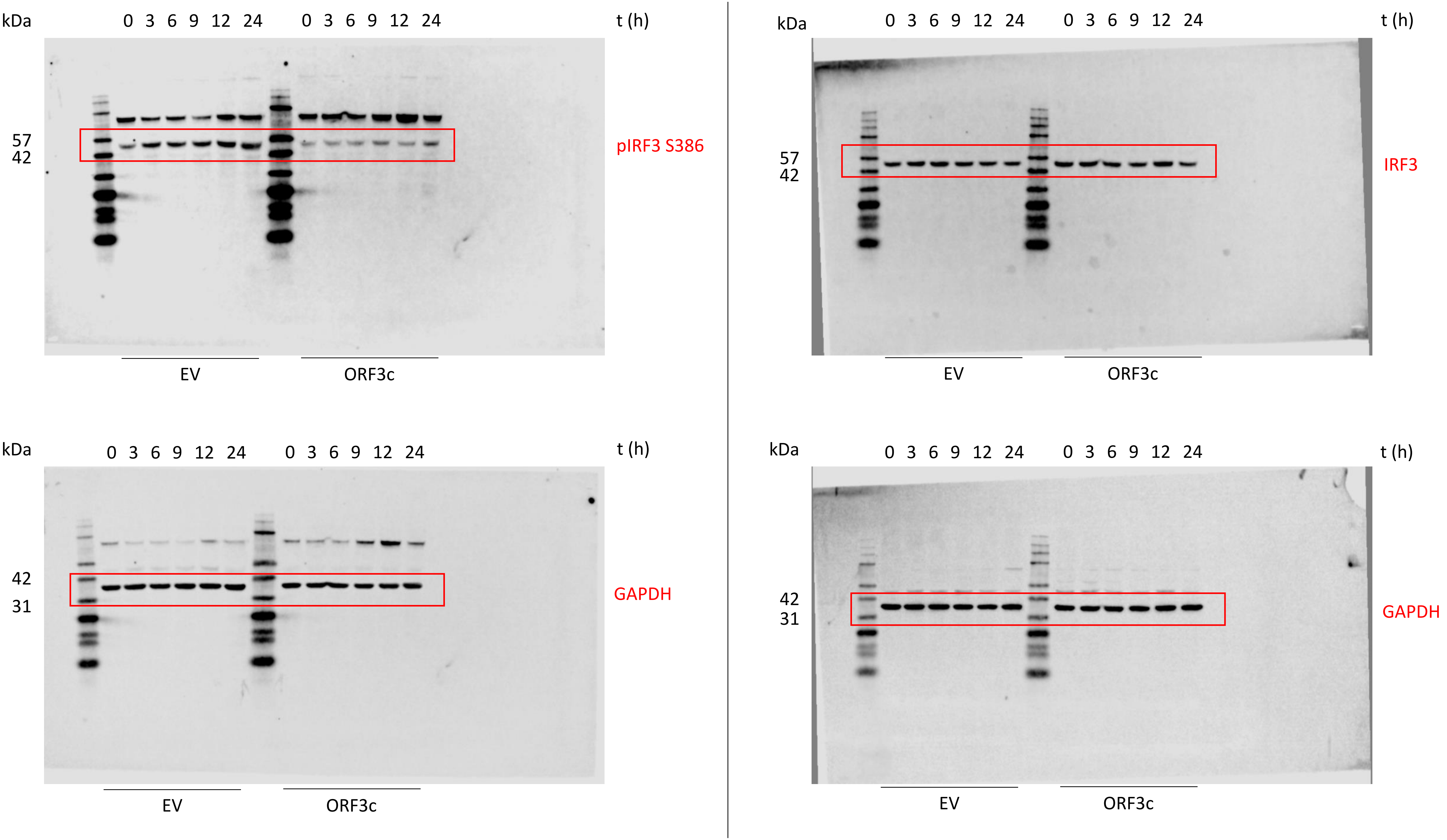
kDa
0 3 6 9 12 24 0 3 6 9 12 24
t (h)
57
42
pIRF3 S386
EV
ORF3c
kDa
0 3 6 9 12 24 0 3 6 9 12 24
t (h)
57
42
IRF3
EV
ORF3c
kDa
0 3 6 9 12 24 0 3 6 9 12 24
t (h)
42
31
GAPDH
EV
ORF3c
kDa
0 3 6 9 12 24 0 3 6 9 12 24
t (h)
42
31
GAPDH
EV
ORF3c
