# Supplementary material for: ORF3c is expressed in SARS‐CoV‐2‐infected cells and inhibits innate sensing by targeting MAVS
Source: EMBO Rep. 2023 Oct 23;24(12):e57137. doi: 10.15252/embr.202357137 (PMC10702836; doi:10.15252/embr.202357137)
Supplement: Supplementary file 3 — Source Data for Expanded View [file EMBR-24-e57137-s002.zip › EMBOR-2023-57137V3_SourceDataForExpandedView/EMBOR-2023-57137V3_SourceDataForExpandedView4.zip!/EV4A/EMBOR-2023-57137V2_SourceDataForExpandedView4.pdf]

Input

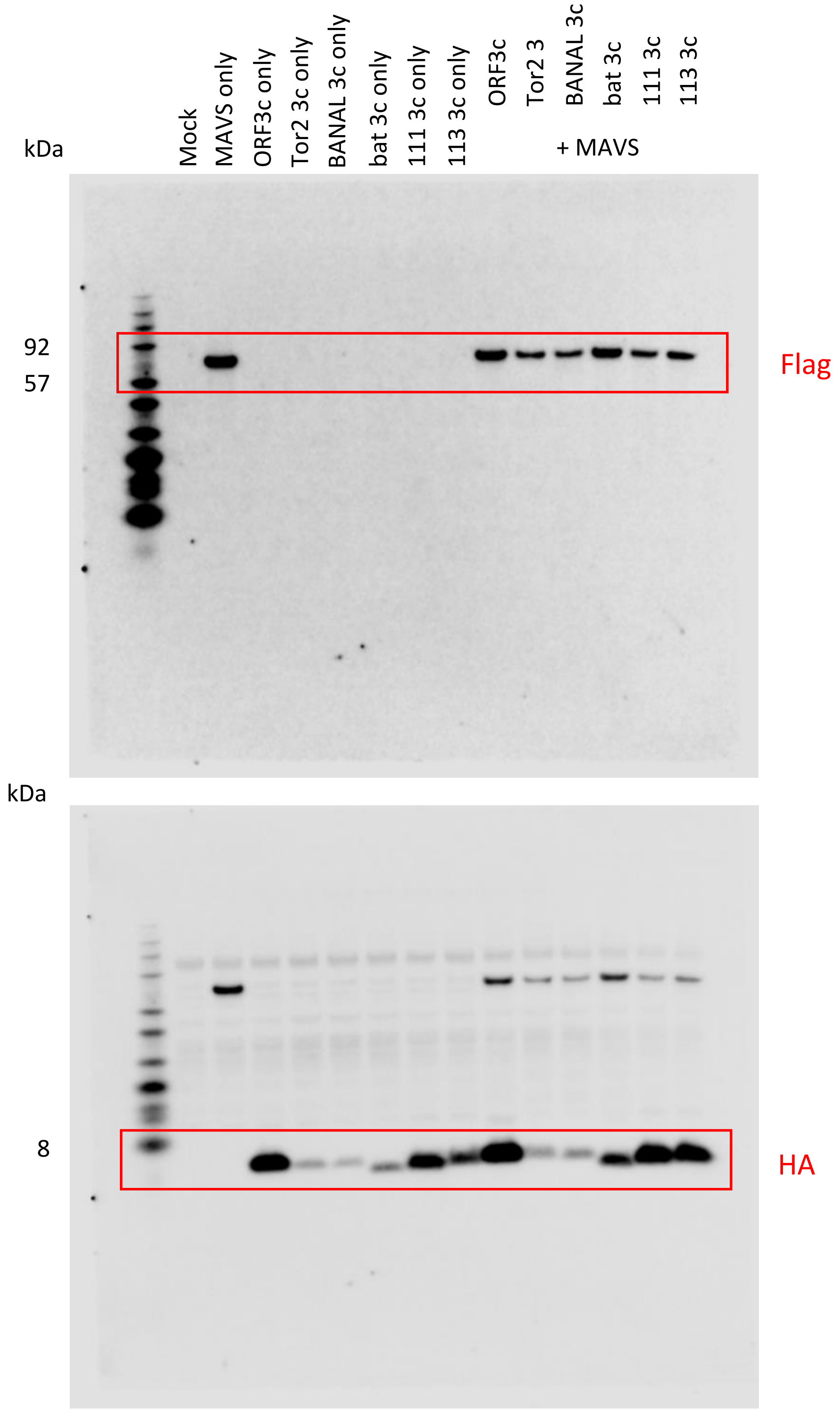
Mock
MAVS only
ORF3c only
Tor2 3c only
BANAL 3c only
bat 3c only
111 3c only
113 3c only
ORF3c
Tor2 3
BANAL 3c
bat 3c
111 3c
113 3c
+ MAVS
kDa
92
57
Flag
kDa
8
HA

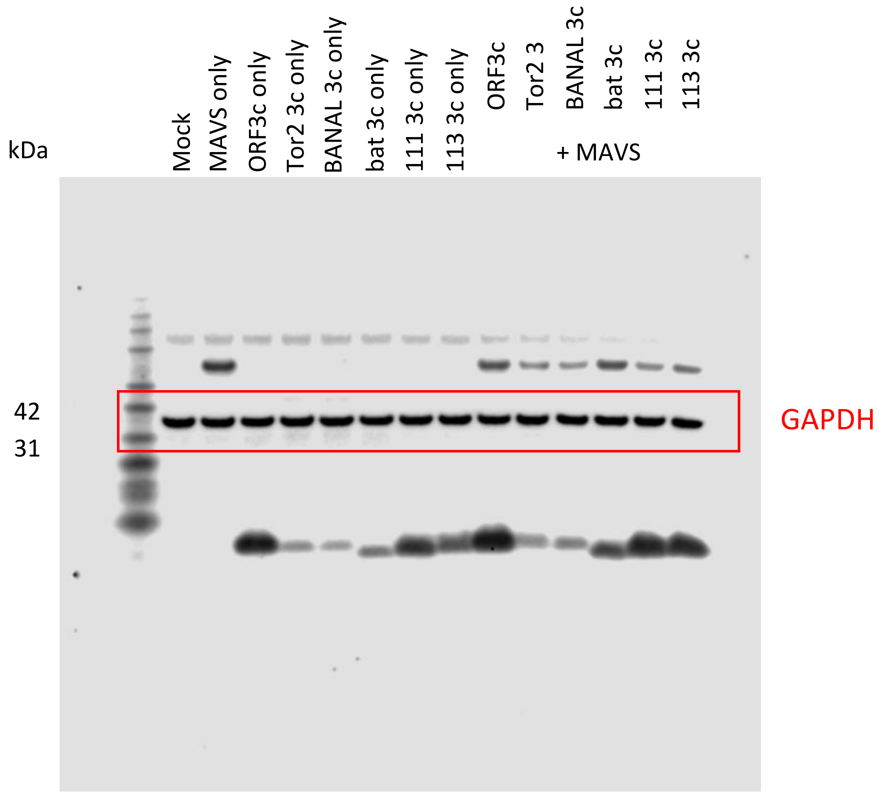
Mock
MAVS only
ORF3c only
Tor2 3c only
BANAL 3c only
bat 3c only
111 3c only
113 3c only
ORF3c
Tor2 3
BANAL 3c
bat 3c
111 3c
113 3c
+ MAVS
kDa
42
31
GAPDH

IP

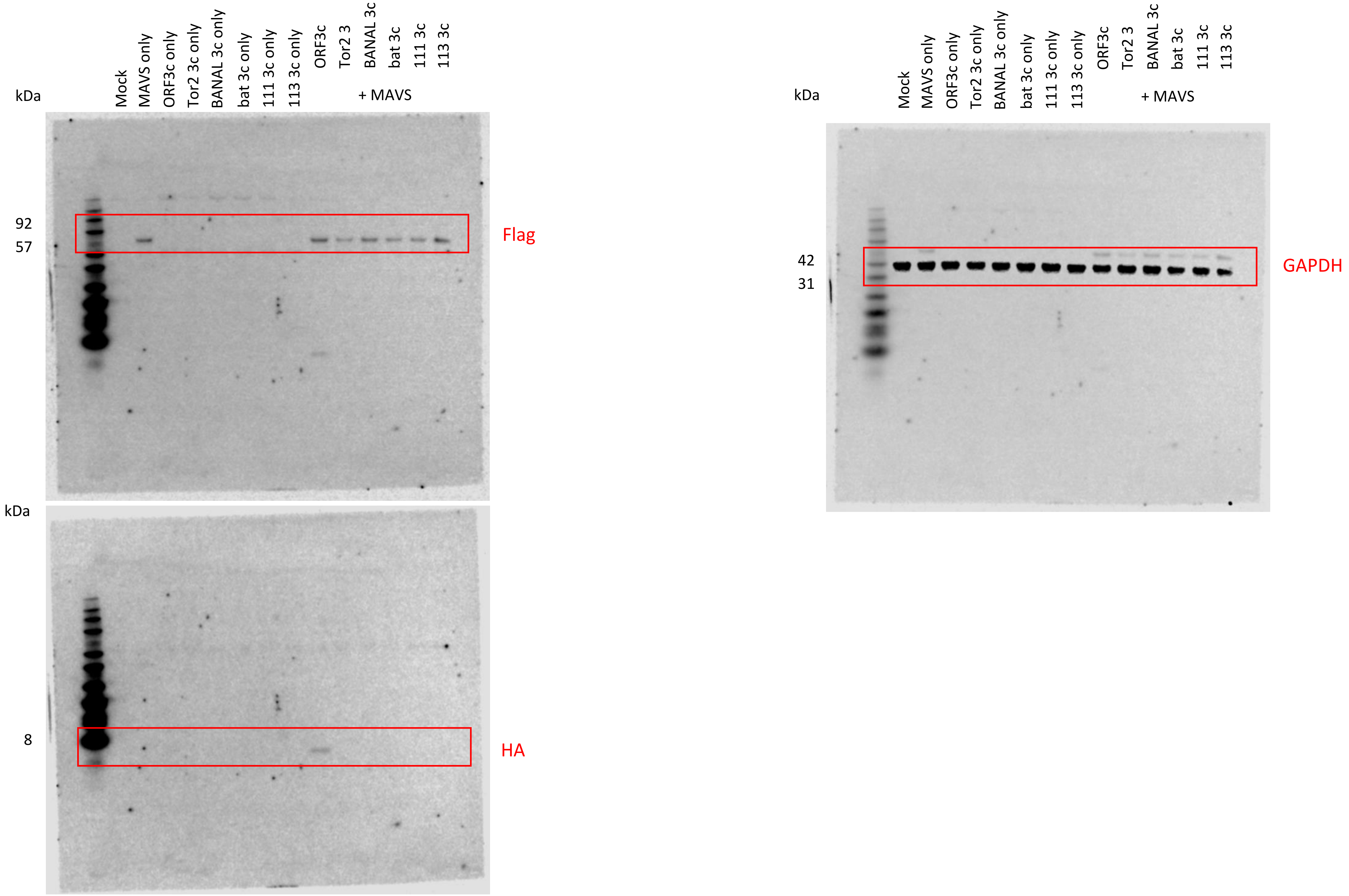
kDa
Mock
MAVS only
ORF3c only
Tor2 3c only
BANAL 3c only
bat 3c only
111 3c only
113 3c only
ORF3c
Tor2 3
BANAL 3c
bat 3c
111 3c
113 3c
+ MAVS
92
57
Flag
kDa
8
HA
kDa
Mock
MAVS only
ORF3c only
Tor2 3c only
BANAL 3c only
bat 3c only
111 3c only
113 3c only
ORF3c
Tor2 3
BANAL 3c
bat 3c
111 3c
113 3c
+ MAVS
42
31
GAPDH
