# Supplementary material for: ORF3c is expressed in SARS‐CoV‐2‐infected cells and inhibits innate sensing by targeting MAVS
Source: EMBO Rep. 2023 Oct 23;24(12):e57137. doi: 10.15252/embr.202357137 (PMC10702836; doi:10.15252/embr.202357137)
Supplement: Supplementary file 3 — Source Data for Expanded View [file EMBR-24-e57137-s002.zip › EMBOR-2023-57137V3_SourceDataForExpandedView/EMBOR-2023-57137V3_SourceDataForExpandedView5.zip!/EV5B/EMBOR-2023-57137V2_SourceDataForExpandedView5B.pdf]

SARS-CoV-2 ΔORF6-YFP

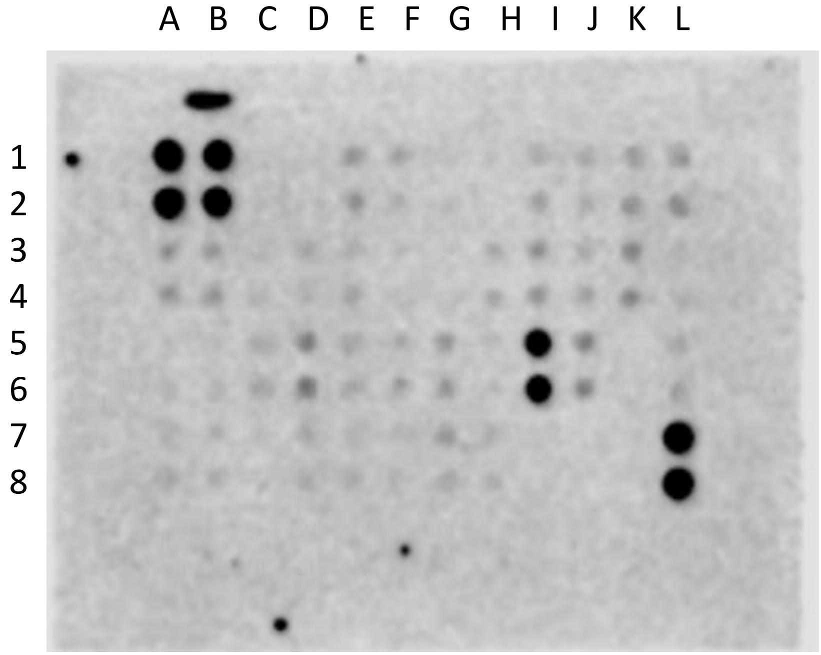

SARS-CoV-2 ΔORF6-YFP ΔORF3c

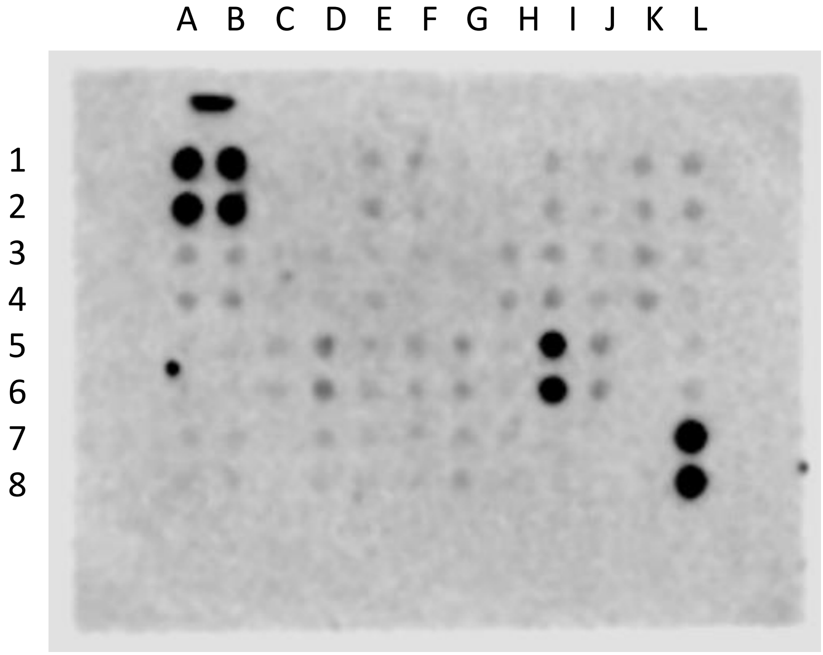

Each antibody is spotted in duplicate vertically

	A	B	C	D	E	F	G	H	I	J	K	L
1<br>2	POS	POS	NEG	NEG	Activin R2A	Biglycan	CA13	CA2	CA72-4	CLEC-2	c-Myc	Cystatin D
3<br>4	Erythropoietin	FCRL5	FGF-16	GATA4	GFR alpha-1	GFR alpha-2	Granzyme B	Granzyme H	HIF-1 alpha	PAPP-A	IFN-beta	IL-17 RC
5<br>6	IL-19	IL-20 R beta	IL-22	ILT-4	LAIR2	LSECtin	Netrin-4	Norrin	NRG1-alpha	PD-L2	PDX-1	Podocalyxin
7<br>8	RGM-C	S100 A1	Semaphorin 6A	SLITRK5	SR-AI	ST6GAL1	Thyroid Peroxidase	Troponin C	Blank	Blank	Blank	POS

RayBio C16 membrane layout
